# A revision of the *Solanum elaeagnifolium* clade (Elaeagnifolium clade; subgenus Leptostemonum, Solanaceae)

**DOI:** 10.3897/phytokeys.84.12695

**Published:** 2017-08-07

**Authors:** Sandra Knapp, Eva Sagona, Anna K.Z. Carbonell, Franco Chiarini

**Affiliations:** 1 Department of Life Sciences, Natural History Museum, Cromwell Road, London SW7 5BD, United Kingdom; 2 Orto Botanico Forestale di Abetone, Associazione Ecomuseo della Montagna Pistoese, Palazzo Achilli, Piazzetta Achilli n. 7 - 51028 Gavinana, Pistoia (PT), Italy; 3 Biological and Environmental Sciences, University of Stirling, Stirling FK9 4LA, United Kingdom; 4 Instituto Multidisciplinario de Biología Vegetal (IMBIV), CONICET-UNC, Universidad Nacional de Córdoba, Córdoba, Argentina

**Keywords:** amphitropical, conservation status, invasive species, lectotypification, Leptostemonum, New World, preliminary conservation status, Solanaceae, spiny solanums, weeds

## Abstract

The *Solanum elaeagnifolium* clade (Elaeagnifolium clade) contains five species of small, often rhizomatous, shrubs from deserts and dry forests in North and South America. Members of the clade were previously classified in sections *Leprophora*, *Nycterium* and *Lathyrocarpum*, and were not thought to be closely related. The group is sister to the species-rich monophyletic Old World clade of spiny solanums. The species of the group have an amphitropical distribution, with three species in Mexico and the southwestern United States and three species in Argentina. *Solanum elaeagnifolium* occurs in both North and South America, and is a noxious invasive weed in dry areas worldwide. Members of the group are highly variable morphologically, and this variability has led to much synonymy, particularly in the widespread *S. elaeagnifolium*. We here review the taxonomic history, morphology, relationships and ecology of these species and provide keys for their identification, descriptions, full synonymy (including designations of lectotypes) and nomenclatural notes. Illustrations, distribution maps and preliminary conservation assessments are provided for all species.

## Abstract (es)

El clado Elaeagnifolium contiene cinco especies arbustivas, usualmente rizomatosas, distribuidas en desiertos y bosques secos de Norte y Sudamérica. Previamente, los miembros del clado estuvieron agrupados en las secciones *Leprophora*, *Nycterium* y *Lathyrocarpum* porque se pensaba que no estaban estrechamente relacionados. El grupo es hermano de los *Solanum* del Viejo Mundo (Old World clade). Las especies del grupo tienen una distribución anfitropical, con tres especies de México y el Suroeste de Estados Unidos y tres especies en Argentina. *Solanum elaeagnifolium* se encuentra en Norte y Sudamérica, y es conocida como una nociva hierba invasora en áreas secas de todo el mundo. Los miembros de este grupo son altamente variables en su morfología, lo cual ha llevado a la existencia de mucha sinonimia, particularmente en *S. elaeagnifolium*. Aquí revisamos la historia taxonómica, morfología, relaciones y ecología de estas especies; así mismo, proveemos claves para su identificación, descripción, sinonimia completa (incluyendo las designaciones de lectotipos) y notas nomenclaturales. Además proveemos ilustraciones, mapas y evaluaciones del estado de conservación de las especies.

## Introduction

*Solanum* L. is one of the ten most species-rich genera of flowering plants (Frodin 2004) and has approximately 1,400 species occurring on all temperate and tropical continents. The highest diversity of both groups and species is in tropical South America, concentrated in a circle around the Amazon Basin (see Knapp 2002b), but significant diversity occurs in various parts of the Old World. *Solanum* was one of Linneaus’s (1753) larger genera, with 23 species mostly described from European or African collections. The last time *Solanum* was monographed in its entirety was in De Candolle’s *Prodromus* (Dunal 1852), which included 901 species (with an additional 19 recorded as incompletely known by him at the time). Until the 21^st^ century, the taxonomy of *Solanum* was largely limited to rearrangements of infrageneric taxa, species-level treatments of smaller groups within the genus, and floristic works.

The large size of *Solanum* and its poorly understood infrageneric structure has meant that *Solanum* taxonomy proceeded in a piecemeal fashion until relatively recently and the genus acquired a reputation of being intractable. A project funded by the United States National Science Foundation’s Planetary Biodiversity Inventory (PBI) program begun in 2004 sought to accelerate species-level taxonomic work across the genus and resulted in a series of monographic and phylogenetic treatments from both Old and New Worlds (e.g., Tepe and Bohs 2011; Stern et al. 2013; Knapp 2013a; Knapp and Vorontsova 2016; Clark et al. 2015; Wahlert et al. 2014, 2015; Aubriot et al. 2016; Vorontsova and Knapp 2016; Spooner et al. 2016). An electronic monographic treatment of the entire genus, with species and species groups added as they are completed, is available online in the web resource Solanaceae Source (http://www.solanaceaesource.org). This treatment is part of that collaborative effort.

## Taxonomy and relationships

*Solanum* has been divided into 13 major clades (Bohs 2005; Särkinen et al. 2013), of which the spiny solanums (subgenus Leptostemonum Bitter, or the Leptostemonum clade) is the largest, with some 550 currently accepted species. The *Solanum elaeagnifolium* species group is part of this large group, and within that, is sister to the monophyletic Old World clade (see Vorontsova et al. 2013).

Plants collected by William Houstoun on the Caribbean coast of Mexico were cultivated by Philip Miller of the Chelsea Physic Garden in London and were described as *S. carolinense* Mill. (= *S. houstonii* Martyn; Miller 1768). *Solanum elaeagnifolium* Cav. was described from material grown in the Real Jardín Botánico de Madrid collected on voyages made by Spanish explorers in the early 19^th^ century (Cavanilles 1800); *S. leprosum* Ortega was probably described from the same living material (see Knapp 2013b). Dunal (1813) treated both species as members of his section Leprophora Dunal, along with *S. sericeum* Ruiz & Pav. (now recognised as a member of the Potato clade, Särkinen et al. 2015), based on their whitish grey pubescence and leaf morphology. A year later, Dunal (1814) described *S. tridynamum* Dunal (=*S. houstonii*) based upon the drawings of José Sessé and Mariano Mociño he had seen either at the herbarium in Geneva or in Montpellier (Knapp 2008b), but did not associate his new species with *S. elaeagnifolium*. Based on the collections made by Alexander von Humboldt and Aimé Bonpland during their brief stay in Mexico, Dunal (1816) described *S. obtusifolium* Dunal (= *S. elaeagnifolium*) as an additional species in his *Leprophora*. Bentham (1844) recognised the similarity of his *S. hindsianum* from Baja California with Mexican and South American populations of *S. elaeagnifolium*, and with populations from Texas (that he suggested represented an additional undescribed taxon).

Exploration of the western United States along with the trade in seeds between European botanic gardens led to the description of many synonyms of the extremely variable species *S. elaeagnifolium* (e.g., *S. flavidum* Torr. described as growing “from Mississippi River to Rocky Mtns”) and *S. houstonii* (e.g., *S. herbertianum* Paxt., described as of unknown origin but with detailed descriptions of methods of cultivation).

In his treatment of the Solanaceae for Candolle’s *Prodromus* Dunal (1852) placed his *S. tridynamum*, plus all of the names we consider synonymous with it, as a member of his group “Nycterium” that was characterized by having unequal anthers (see discussion of *S. houstonii*). Other species placed by him in this group were *S. dubium* Fresen. (= *S. coagulans* Forssk.) from northern Africa, *S. vespertilio* Aiton of the Canary Islands and *S. wightii* Nees of India. Convergent evolution of heterandry has been well-documented in *Solanum*, and has led to the break-up of many of Dunal’s groups based on this character (Lester et al. 1999; Anderson et al. 2006; Bohs et al. 2007). The Old World species with heteromorphic anthers do not form a monophyletic group either (Vorontsova et al. 2013; Aubriot et al. 2016). Dunal placed *S. elaeagnifolium*, *S. hindsianum* and many species of Australian spiny solanums (e.g., *S. pungetium* R.Br.) in a heterogenous un-named group characterized by having plicate corollas.

In floristic works *S. elaeagnifolium* has in general been treated as being native to Mexico (e.g., Morton 1976), based on Cavanilles’ locality of “America calidiore” (Cavanilles 1795), and the name *S. leprosum* had been used for South American material, either as a species or at infraspecific rank (Morton 1976). The material used by Cavanilles (and most likely also by Ortega, see Knapp 2013b) is said to be from “el viaje de los españoles alrededor del Mundo” (the voyage of the Spanish around the world), and could therefore be from either South or North America.

Description of the Argentine endemics *S. mortonii* Hunz. and *S. homalospermum* Chiarini occurred relatively recently (Hunziker 1979; Chiarini 2004). Hunziker (1979) suggested that *S. mortonii* was closely related to *S. juvenale* Thell. and *S. conditum* C.V.Morton, both now recognised as members of the Carolinense clade (section Lathyrocarpum, Wahlert et al. 2015). Chiarini (2004) recognised the similarities of *S. homalospermum* to *S. elaeagnifolium*, but also suggested it was closely related to the *S. multispinum* N.E.Br. group of Whalen (1984).

All of the members of this small species group were only recognised as being related as the result of molecular phylogenetics (Levin et al. 2006; Stern et al. 2011; Wahlert et al. 2014). Whalen (1984) suggested that *S. elaeagnifolium* was related to Australian species and placed it in his *S. ellipticum* group, and placed *S. houstonii* (as *S. tridynamum*) in his *S. vespertilio* group, based on its zygomorphic flowers; he included *S. hindsianum* in his unplaced species. In his treatment of groups of New World solanums Nee (1999) placed *S. elaeagnifolium*, *S. hindsianum* and *S. houstonii* (as *S. tridynamum*) with *S. vespertilio* Aiton and *S. lidii* Sunding (both Canary Island endemics with strongly zygomorphic flowers, see Anderson et al. 2006) in an un-named series (Series 4) of subsection Lathyrocarpum G.Don. He placed *S. mortonii* in “Series 2” along with other species such as *S. comptum* C.V.Morton and *S. pumilum* Dunal, most of which are now currently recognised as members of the Carolinense clade (section Lathyrocarpum; Wahlert et al. 2015), but others are species of uncertain affinity (e.g., *S. euacanthum* Phil. is weakly sister to the Elaeagnifolium clade, see Wahlert et al. 2014; *S. multispinum* N.E.Br. is a species of uncertain affinities; see Stern et al. 2011, Wahlert et al. 2014). In combined analyses using plastid and nuclear markers *S. elaeagnifolium* accessions from North and South America group together as sister taxa (Stern et al. 2011; Wahlert et al. 2014). Stern et al. (2011) recovered *S. mortonii* as sister to *S. elaeagnifolium* (both North and South American accessions). *Solanum homalospermum* was recovered as a member of the Elaeagnifolium clade by Wahlert et al. (2014), and as sister to the grouped *S. elaeagnifolium* accessions used in that study, with *S. mortonii*. Wahlert et al. (2014, see their Fig. 1), in the most recent phylogenetic treatment of these taxa, recovered two groups within the Elaeagnifolium clade – one consisting of the South American species plus all accessions of *S. elaeagnifolium*, and the other with *S. hindsianum* + *S. houstonii*, both from North America.

**Figure 1.**
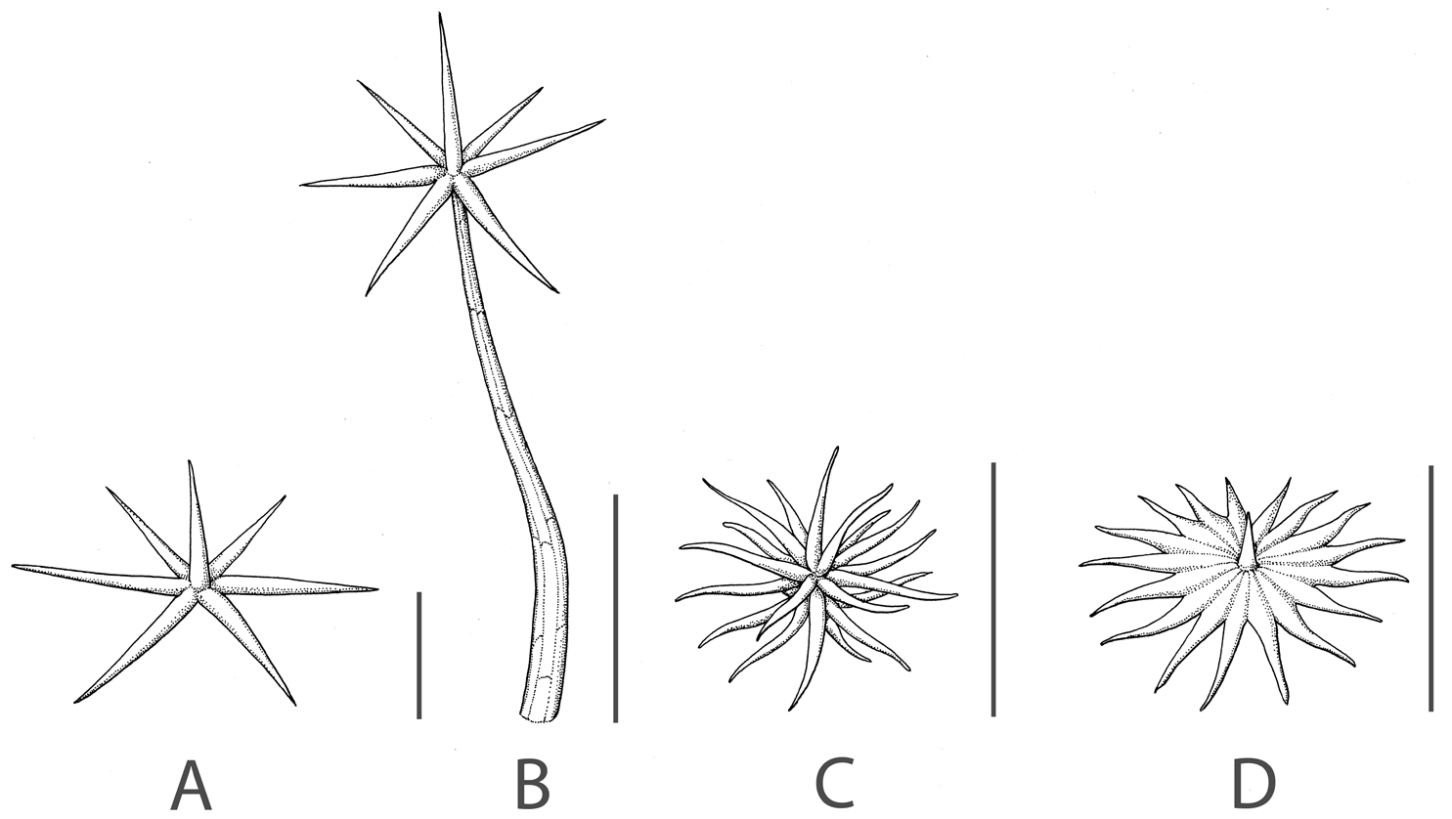
Trichome morphology representative of that found in members of the Elaeagnifolium clade. **A** Sessile porrect-stellate trichome **B** Stalked porrect-stellate trichome **C** Multiangulate trichome **D** Lepidote trichome. Scale bars = 0.5 mm. Reproduced with permission from Systematic Botany Monographs 99 (Vorontsova and Knapp 2016).

### Morphology

Habit and stems. Members of the *S. elaeagnifolium* group are all small to medium-sized shrubs with rhizomatous underground stems. The plants are usually less than 1 m and rarely exceed 2 m tall, although label data indicate *S. hindsianum* can grow to reach 3 m in height. Due to their underground stems, plants are often found in dense colonies, often in disturbed areas (see below). The roots of *S. elaeagnifolium* have been characterised as tuberised (“tuberizada”; Cosa et al. 1998) with up to 20 cortical layers. Only the vertical underground parts are thus thickened, the horizontal spreading stems (rhizomes) are not corky and thickened.

The stems of all members of the group are variably prickly; this variability is most pronounced in *S. elaeagnifolium*, but occurs in all the species of the clade (see discussion of *S. houstonii*). Prickles and their morphology are described in detail below. Prickly and non-prickly morphs of the same species have often been described as different species or forms, leading to much synonymy.

Sympodial growth is characteristic of Solanaceae giving the stems a typical “zig-zag” appearance; details of sympodial structure have proved useful for infrageneric classification within *Solanum* (Child and Lester 1991; Knapp 2002b). Vegetative growth is initially monopodial, but with the onset of flowering, becomes sympodial. The inflorescence is developmentally terminal, and stem continuation is initiated in the axil of the leaf below each inflorescence. Each lateral shoot with alternate leaves arranged in a 1/3 phyllotaxic spiral and a terminal inflorescence is termed a sympodial unit. In some cases, when the axes of sympodial units are fused, the inflorescences appear to originate laterally from the middle of an internode; and when growth of the axes is suppressed, the leaves appear paired (geminate) at a node (Danert 1958). All of the members of the *S. elaeagnifolium* group have difoliate sympodial units with leaves not strongly paired at the nodes, but occasionally sympodial units are tri- or plurifoliate.

Leaves. Leaf morphology in subgenus Leptostemonum is very diverse, not only among groups of species, but also within groups and even within individuals of a single species. The highly variable size, shape, and lobing of leaves are often the first characters to be noticed by herbarium taxonomists, and attributing undue importance to this variability is one of the causes of excessive synonymy. All members of the *S. elaeagnifolium* group have simple leaves with entire or lobed margins. Leaves on pre-reproductive shoots are usually more lobed than those of reproductive shoots; this is common in many spiny solanums (see Vorontsova and Knapp 2016). Some populations of *S. houstonii* from the western coast of Mexico (Sinaloa and Sonora) have deeply lobed leaves (see description of *S. houstonii*).

Leaf size also varies with season and environmental conditions; plants collected in the wet season or from wetter areas always have larger leaves than those from drier microhabitats or that were collected in the dry season.

**Figure 2.**
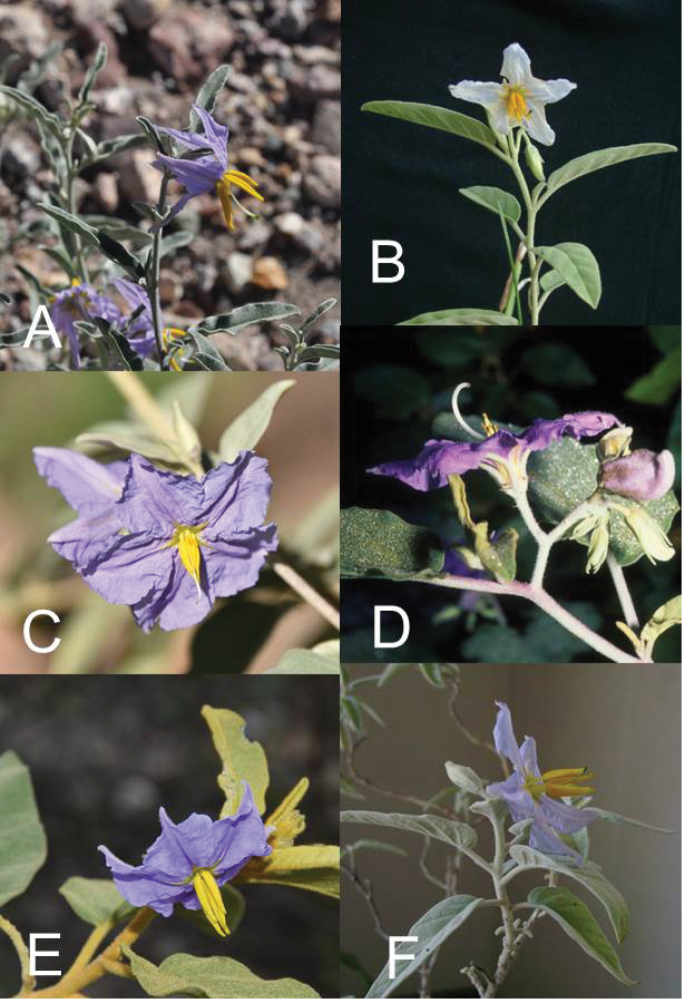
Flowers of members of the Elaeagnifolium clade. **A** *Solanum elaeagnifolium*, with divergent anthers of approximately equal size and shape (Argentina, Mendoza, *Knapp et al. 10470*) **B** *Solanum homalospermum* (Argentina, Catamarca, *Chiarini et al. 505*) **C** *Solanum hindsianum* (cultivated in Arizona) **D** *Solanum houstonii*, hermaphroditic flower and strongly curved buds (Mexico, Yucatán, *Peña-Chocarro et al. 407*) **E** *Solanum houstonii*, staminate flower (Mexico, Querétaro, *Ochoterena et al. 976*) **F** *Solanum mortonii* (cultivated in Córdoba; from *Barboza et al. 644*). Photographs **A, D, E** by S. Knapp; **B, F** by F. Chiarini; **C** by Eugene Sturla.

Pubescence. In common with the rest of the Leptostemonum clade, species in the *S. elaeagnifolium* group have stellate pubescence. Minute simple trichomes (papillae) are also present, usually on the new growth. Trichomes are generally similar throughout the plant in these species, but pubescence density is usually greater on leaf undersides. Stellate trichomes in the group can be characterized as one of three types: 1) porrect, with straight unicellular rays arranged horizontally in a single plane, and a unicellular midpoint perpendicular to the rays (Fig. 1A, B); 2) multangulate, i.e., with the numerous rays arranged in more than one plane, (Fig. 1C); or 3) lepidote, with the acicular rays fused near the centre (Fig. 1D) to form a scutellate or shield-like structure around a midpoint of variable length (Carvalho et al. 1991; D’Arcy 1992; Roe 1971).

*Solanum elaeagnifolium* is characterised by its lepidote pubescence, but some individuals of *S. hindsianum* can have porrect stellate trichomes with some fusion of the ray bases; these trichomes, however, never develop into the typical shield-like structure found in *S. elaeagnifolium*. The basal cells of some of the lepidote trichomes in *S. elaeagnifolium* are inserted into the mesoderm (Cosa et al. 1998; Bruno et al. 1999); Bruno et al. (1999) suggest this is related to water conservation in the xeric environments in which *S. elaeagnifolium* grows. *Solanum homalospermum* has midpoints equal to or longer than the rays, while other taxa usually have reduced midpoints. *Solanum mortonii* has multangulate trichomes on all parts.

**Figure 3.**
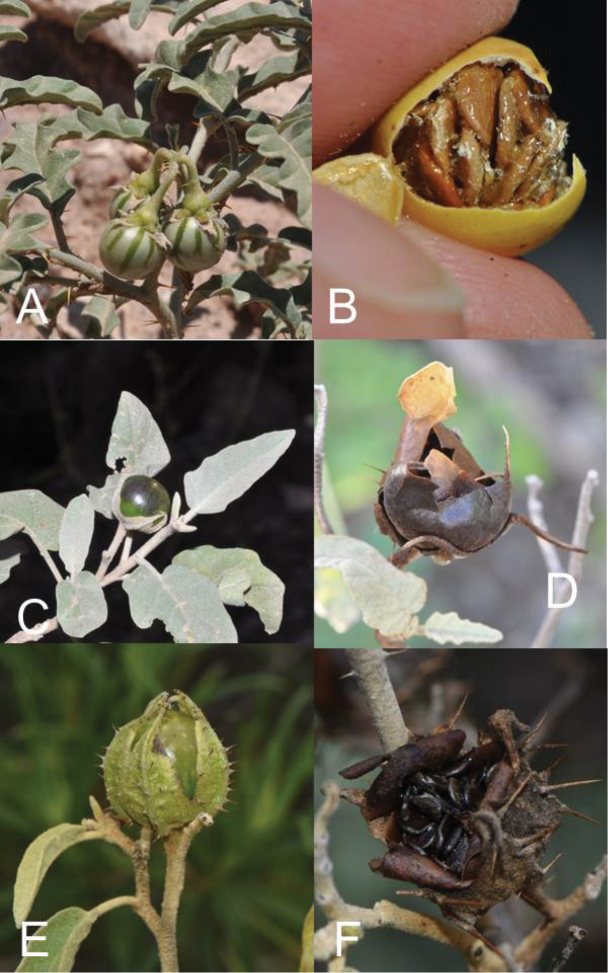
Fruits of members of the Elaeagnifolium clade. **A** *Solanum elaeagnifolium*, immature berries with green mottling (Argentina, Córdoba, *Barboza et al. 3434*) **B** *Solanum elaeagnifolium*, mature fruit with sticky tan seeds (Argentina, Córdoba, *Barboza et al. 3435*) **C** *Solanum mortonii*, immature fruit exerted from the calyx (Argentina, Catamarca, *Barboza et al. 3439*) **D** *Solanum mortonii*, mature fruit exerted from the calyx (Argentina, Catamarca, *Barboza et al. 3438*) **E** *Solanum houstonii*, immature fruit enclosed in accrescent calyx (Mexico, Querétero, *Ochoterena et al. 976*) **F** *Solanum houstonii*, mature fruit enclosed in accrescent calyx with black seeds (Mexico, Querétero, *Ochoterena et al. 976*). Photographs by S. Knapp.

**Figure 4.**
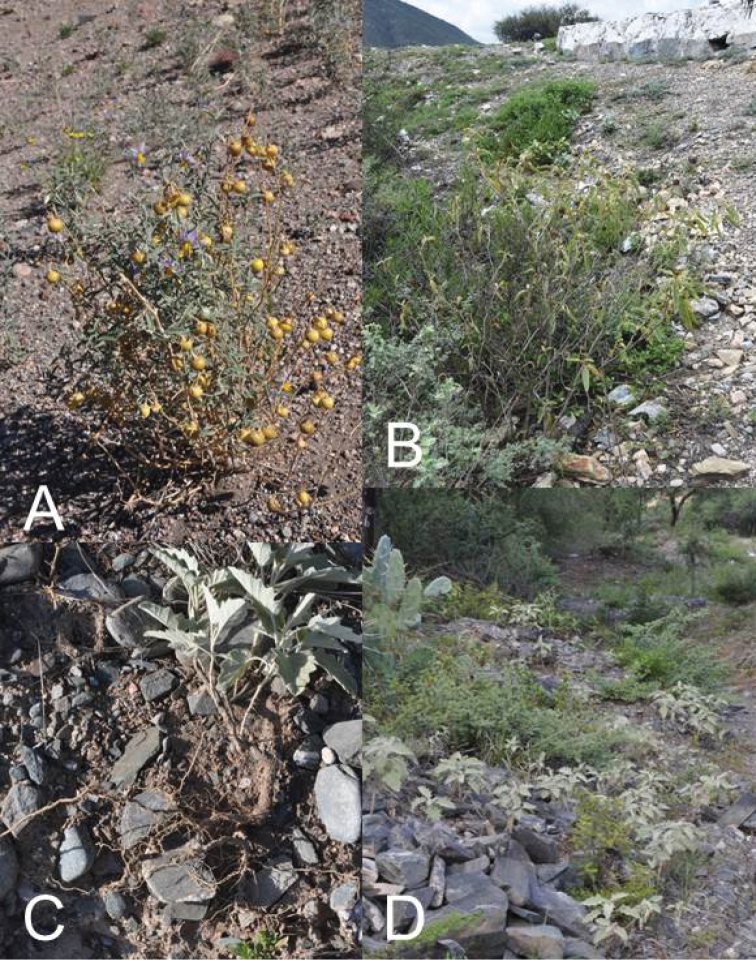
Habitats of members of the Elaeagnifolium clade. **A** *Solanum elaeagnifolium*, old stems with persistent berries and young new stems from underground rhizomes (Argentina, Mendoza, *Knapp et al. 10470*) **B** *Solanum houstonii* on rocky slope (Mexico, Querétaro, *Ochoterena et al. 976*) **C** *Solanum mortonii*, plant dug up showing extensive underground stems (Argentina, Catamarca, *Barboza et al. 3437*) **D** *Solanum mortonii*, large population on rocky slopes (Argentina, Catamarca, *Barboza et al. 3437*). Photographs by S. Knapp

In herbarium specimens, the trichomes of all these species are pale grey, giving the plants a silvery cast. On live plants, the trichomes of *S. elaeagnifolium* are similarly silvery (hence the specific epithet referring to *Elaeagnus* L. (Elaeagnaceae, the Russian olive) but trichomes of *S. houstonii* can be golden or reddish yellow (see Fig. 2E, 3E). *Solanum mortonii* has whitish grey trichomes that are much denser on the leaf undersurfaces, making the leaves strongly discolorous (see Fig. 3C, 4C, D).

Prickles. Prickles in *Solanum* are epidermal in origin and are thought to be modified multicellular stellate trichomes with layers of elongate and lignified cells (Whalen 1984). The common origin of trichomes and prickles can be observed on young stems of where some trichomes develop longer lignified stalks that become prickles with an apical stellate trichome that is later deciduous (see for example Fig. 124 in Vorontsova and Knapp 2016). Often prickles can themselves bear trichomes, reflecting their epidermal nature. The development of prickles in *Solanum* has not been studied in detail in any species.

Prickles can occur on all above-ground parts of a plant except the corolla and the fruit. Density and distribution of prickles vary with the age of the plant and environmental conditions and, thus, are not particularly useful characters; all species of the group can have branches (see Figs 2, 3 and species illustrations) or entire individuals with no prickles. This plasticity has led to much taxonomic confusion (Jaeger 1985). Where prickles are present they are usually straight and acicular, but the stem prickles of *S. hindsianum* and *S. houstonii* are broader at the base and occasionally somewhat curved. On an individual plant prickles are usually uniform throughout the plant. *Solanum hindsianum* and *S. mortonii* usually lack prickles on the leaves.

Inflorescences. As with all species of *Solanum*, the inflorescence in members of the *S. elaeagnifolium* clade is developmentally terminal, and is later overtopped by the leading axillary shoot making it appear lateral. The basic inflorescence, as in all other species of *Solanum*, is a scorpioid cyme that is branched or unbranched. In *Solanum* the inflorescence expands from the tip with each apical meristem producing multiple flowers in a proliferating manner (Lippmann et al. 2008). Inflorescences of members of the *S. elaeagnifolium* clade are usually unbranched (simple), but *S. houstonii* rarely has forked inflorescences (see Fig. 2 and individual species illustrations). In strongly heteromorphic species (*S. homalospermum*, *S. houstonii*) the solitary basal (hermaphroditic) flower is borne very near the base, and the more distal staminate flowers are borne at some distance from it.

Calyces. Members of the Elaeagnifolium clade have 5-merous flowers like most other species of *Solanum*, but occasional tetramerous individuals do occur. The calyx in all members of the group is composed of deltate lobes with prominent keels on the abaxial surface, and with narrow, elongate acumens usually as long as or longer than the deltate portion of the lobes (Figs 2 and 3). Staminate flowers of *S. houstonii* have non-prickly, shorter calyces than those of hermaphroditic flowers which are ca. 1.5 times larger and usually densely prickly (see Fig. 2D, 3E). The degree to which the calyx is accrescent in fruit varies in the species of the group, and can be a useful identification character (see Fig. 3). The calyx is not markedly accrescent in *S. elaeagnifolium*, where the berry is clearly visible, partly accrescent and covering about half of the berry in *S. hindsianum*, and markedly accrescent in *S. houstonii*, *S. homalospermum* and *S. mortonii*, where it completely encloses the mature berry. We have seen some specimens of *S. elaeagnifolium*, however, with accrescent calyces (e.g., *Barkley 14-A539* from Cochise County, Arizona USA) and some collections of *S. houstonii* with somewhat exposed berries (e.g., *Dorantes et al. 1033* from Veracruz, Mexico), suggesting this character might be quite variable in individual species or that in *S. houstonii* the calyx splits with fruit maturity and drying.

Corollas. Like the calyces, corollas of members of the Elaeagnifolium clade are most often 5-merous. Corollas are stellate, and usually divided about halfway to the base, the lobes are deltate to triangular with copious to sparse interpetalar tissue, and are usually spreading at anthesis (see Fig. 2). The corollas of *S. elaeagnifolium*, *S. hindsianum*, *S. homalospermum* and *S. mortonii* are actinomorphic, with all lobes the same size and shape. *Solanum houstonii*, however, has markedly zygomorphic corollas, with the two lower lobes enlarged relative to the upper ones (see Fig. 2D, E; Fig. 10), and with corolla shape differing between hermaphroditic and staminate flowers. Staminate flowers (S) normally have slightly larger corollas than hermaphrodites (H)(length: H: 37.8 ± 1.3, S: 40.0 ± 1.2; width: H: 38.8 ± 1.4, S: 41.8 ± 1.2, A.Z.K. Carbonell, measurements from field individuals), and are more zygomorphic.

The abaxial surfaces of the corolla lobes are densely pubescent with stellate trichomes where they are exposed in the bud; the interpetalar tissue in the sinuses is glabrous on both surfaces in all species. In exceptionally prickly individuals of *S. houstonii* a few minute prickles are borne on the abaxial midvein of each corolla lobe.

Androecium. The anthers of all members of the Eleagnifolium clade, like those of most of the spiny solanums, are long-tapering, with distally directed pores that do not lengthen to slits with age. In all five species the anthers are heteromorphic, with three of the five slightly longer than the rest, and are usually slightly curved (see Fig. 2 and individual species illustrations). The anthers of *S. elaeagnifolium* are the least heteromorphic, and those of *S. houstonii* the most. In *S. houstonii* staminate flowers have three long and curved and two short and straight anthers, while those hermaphroditic flowers are similar but are not as unequal or curved as the anthers of staminate flowers. These anthers in hermaphroditic flowers of *S. houstonii* are very similar to those of *S. hindsianum*.

**Figure 5.**
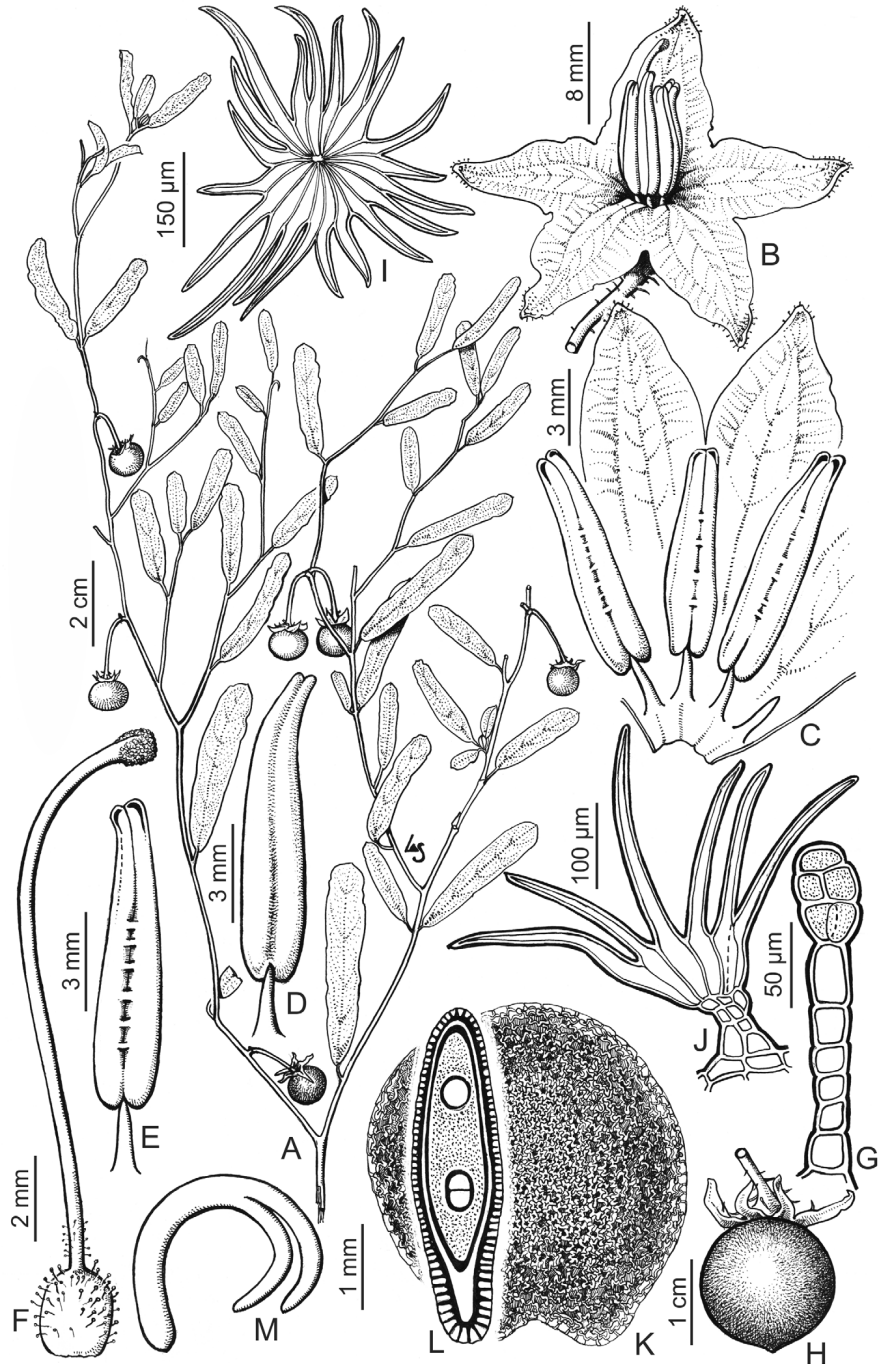
*Solanum elaeagnifolium* Cav. **A** Fruiting branch **B** Flower **C** Spread corolla **D** Dorsal view of stamen **E** Ventral view of stamen **F** Gynoecium **G** Glandular trichome from gynoecium **H** Fruit **I** Lepidote trichome from calyx, seen from above **J** Lepidote trichome from calyx, seen from the side **K** Seed **L** Transverse section of seed **M** Embryo. Drawn by Leonor Sánchez. Reproduced with permission from Flora Argentina (Chiarini 2013).

Petanatti and Del Vitto (1991) record structures they called “bridas” (flanges) on the anthers of *S. elaeagnifolium* (see Fig. 5E). These small folds in the anther surface are similar to the papillate (papillose) anther surfaces of *S. mortonii* and some species of non-spiny solanum from Madagascar (Chiarini 2007; Knapp and Vorontsova 2016). These papillate anther surfaces occur in some, but not all, herbarium specimens of all members of the Elaeagnifolium clade (see species descriptions). Structures similar to the papillae seen on the abaxial anther surfaces in this group are also found in the Tomato clade (Carrizo-García 2003; Peralta et al. 2008), where the anthers are held tightly together with elongate and hair-like papillae. In tomato, the development of these structures is controlled by a transcription factor similar to that which controls the conical cells of petals (see Glover et al. 2004); ongoing investigations across *Solanum* are being undertaken to assess its presence and function in other taxa (G. Davies and B.J. Glover, pers. comm.).

The filaments in all members of the group are composed of a very short filament tube, and a glabrous free portion that is usually ca. 1/5 the length of the anthers themselves (see Fig. 2). The free portion of the filaments is often somewhat curved in live plants.

Gynoecium. All of the species of the Elaeagnifolium clade except *S. elaeagnifolium* exhibit heterostyly, with long- and short-styled flowers borne in the same inflorescence. The ovary in short-styled flowers in strongly andromonoecious species such as *S. houstonii* is vestigial and the style is very short. The long style in hermaphroditic flowers (and in all flowers of *S. elaeagnifolium*) is slightly curved and usually white (see Fig. 2); styles are usually pubescent in the basal part with porrect-stellate trichomes. The style in *S. houstonii* curves in a similar way to the largest anthers in the staminate flowers and the flowers are somewhat enantiostylous. The stigma is bright green and clavate.

Fruit. As with all species of *Solanum*, the fruit is a bicarpellate berry. All of the species in the *S. elaeagnifolium* group have globose berries in which the pericarp dries at maturity. Unripe berries of all species are mottled green (see Fig. 3A), but at maturity they become either yellow or orange (*S. elaeagnifolium*) or dark brownish black (e.g., *S. houstonii*) and the fruit wall becomes brittle and breaks (see Fig. 3D, F). In *S. elaeagnifolium* the berries remain on the plant for months, eventually falling as a unit – the seeds are often fused together with a sticky glue-like substance (Fig. 3B). Other species, especially those in which the calyx is accrescent in fruit and completely covers the berry (*S. houstonii*, *S. mortonii*) break near the apex of the berry and the seeds are released a few at a time over a long period (Lester and Symon 1989). This type of “censer” or spray-cup mechanism is also found in species of Solanum section Androceras (Nutt.) Marzell (e.g., *S. rostratum* Dunal; Whalen 1979) and in some Australian species (e.g., *S. tununduggae* Symon, *S. vansittartensis* C.A.Gardner; Symon 1981). The berry wall in *S. elaeagnifolium*, *S. homalospermum* and *S. mortonii* includes fibres and sclerids of calcium oxalate in an unusual arrangement with the fibres enclosing a crystalline sclerid or group of sclerids (see Fig. 3.7 in Chiarini 2007; Chiarini and Barboza 2007a). These inclusions are not known elsewhere in spiny solanums, except in taxa whose berries are dry and dehiscent (e.g., *S. euacanthum*), and may be related to dehiscence and the irregular rupture of the berry wall mediated by changes in temperature and humidity (Chiarini 2007; Chiarini and Barboza 2007a) like that occurring during anther dehiscence (D’Arcy et al. 1996). Berries of *S. hindsianum* and *S. houstonii* have not yet been examined anatomically.

Protein extracts from ripe berries of *S. elaeagnifolium* contain compounds similar to the aspartic proteinases such as rennin and chrymosin used in cheese manufacture (Gutiérrez-Méndez et al. 2012). Tests with the berries suggested that these fruits could be useful in the production of soft cheeses such as cream cheese due to their lower activity than traditional coagulants (Gutiérrez-Méndez et al. 2012); berries are used in local cheese manufacture in indigenous communities in both Mexico and the United States (see description of *S. elaeagnifolium*).

Seeds. Seed morphology has been suggested to be a useful character for species-level taxonomy in *Solanum* (Souèges 1907; Lester and Durands 1984) and has been used to define morphological groups in other clades (e.g., Geminata, see Knapp 2002b). Seeds of members of the Elaeagnifolium clade are quite large relative to most spiny solanums, flattened reniform and have pitted surfaces. Seeds of *S. elaeagnifolium* are pale tan colored (Fig. 3B); all other species have dark brown or black seeds (Fig. 3F). In all species the seeds are often “glued” together in mature fruits with a shiny, sticky substance. Seed characteristics are not useful in distinguishing species in the group, although the thickened margin and warty surface of *S. homalospermum* are distinctive (Chiarini and Barboza 2007b).

Chromosomes. Of the five species in the Elaeagnifolium clade, only *S. mortonii* lacks data for chromosome number (see Table 1). The other four species of the group have chromosome numbers based on 2n = 12 or multiples of 12, like most other *Solanum* species (Chiarini et al. in press). Only a few metaphasic preparations of *S. homalospermum* have been obtained, all with ca. 48 chromosomes, but none of them with exactly the same chromosome number. Karyotypes are available for *S. houstonii* (Chiarini et al. in press) and *S. elaeagnifolium* (Acosta et al. 2012); chromosomes are symmetric, small to medium-sized (1.15 to 2.64 mm), and are similar to those found elsewhere in *Solanum*. Further details of chromosome morphology, such as fluorescent banding patterns and *in situ* hybridization, are only available for *S. elaeagnifolium* (Acosta et al. 2012; Chiarini 2014).

**Table 1.** Chromosome numbers in the Elaeagnifolium clade.

Species	Haploid chromosome number (n)	Reference or voucher
*Solanum elaeagnifolium*	n=12, 24, 36 (Argentina); n=12 (North America)	Moscone 1992; Acosta et al. 2005; Powell and Weedin 2005; Scaldaferro et al. 2012
*Solanum hindsianum*	n=12	Averett and Powell 1972
*Solanum homalospermum*	n= ca. 24	Chiarini et al. in press; *Chiarini 505* (CORD)
*Solanum houstonii*	n=12	Averett and Powell 1972; Chiarini et al. in press; *Bauk s.n.* (CORD)
*Solanum mortonii*	not known	–

In the southern part of its range *S. elaeagnifolium* has three ploidy races; diploid, tetraploid and hexaploid (Scaldaferro et al. 2012). *Solanum homalospermum* also appears to be polyploid (Chiarini et al. in press). A relationship between polyploidy and clonality has been recorded in other groups of angiosperms (Baldwin and Husband 2013; James and McDougall 2014; Wahlert et al. 2015), but in this group diploid populations (*S. elaeagnifolium* in both North and South America) and species (possibly *S. mortonii*, but ploidy status is as yet not known) also appear to reproduce vegetatively. In Argentina, polyploid populations of *S. elaeagnifolium* are generally found in wetter parts of the species range (Scaldaferro et al. 2012). Chiarini et al. (2016) have shown that tetraploid populations have arisen repeatedly from both diploid and hexaploid progenitors, but also show that using plastid markers, all populations at particular ploidy levels cluster together (see discussion of *S. elaeagnifolium*).

### Biology and natural history

Habitats and distribution. Members of the *S. elaeagnifolium* group are generally rather weedy, and grow in open habitats in dry forests and scrublands (Fig. 4). This habit has resulted in *S. elaeagnifolium* becoming an invasive weed on several continents (see discussion of *S. elaeagnifolium*); other species in the group with rhizomatous underground stems (e.g., *S. homalospermum*, *S. mortonii*) also have this potential. *Solanum elaeagnifolium* grows in a wide variety of habitats, from deserts to montane Chaco habitats in Argentina. Scaldaferro et al. (2012) found that polyploid populations were more likely to be growing in wetter habitats in Argentina. *Solanum houstonii* similarly grows across a wide variety of habitats and elevations; it occurs in most of the arid habitats of Mexico (Rzedowski 1978) from the Sonoran Desert zones in western Mexico, across the volcanic belt up to 2000 m to the limestone pans of the Caribbean coast.

The members of the group (excluding the invasive populations of *S. elaeagnifolium*) exhibit a typical New World amphitropical distribution, with species occurring in the arid regions in both hemispheres; this pattern has long been of interest to botanists (see Gray and Hooker 1880; Bray 1900; Raven 1963). Various hypotheses for these patterns have been extinction of intervening tropical ancestral populations (Johnston 1940), island hopping via stepping stones of arid habitat (Raven 1963) or long-distance dispersal (Raven 1963; Simpson and Neff 1985). Analysis of the phylogenetic relationships in groups with amphitropical distributions suggests that, as might be expected, these patterns result from a mixture of causes, even within a given group (Simpson et al. 2005). Raven (1963) hypothesized that desert (arid zone) disjuncts were primarily the result of migration or dispersal from south to north; this is the case in some groups (e.g., Simpson et al. 2005, 2006, *Hoffmannseggia* Cav. and *Pomaria* Cav., Leguminosae) while in others long-distance dispersal appears to have occurred from north to south (e.g., Moore et al. 2006a, 2006b, *Tiquilia* Pers. Boraginaceae). Simpson et al. (2005) showed that the current distribution of *Hoffmannseggia* species was the result of numerous dispersal events from South to North America, rather than a single event followed by diversification. In all studies to date the dispersal events leading to these amphitropical disjunctions are relatively recent. Biogeographic history in the Solanaceae as a whole has involved multiple dispersal events from South to North America (Dupin et al. 2017) and vice versa.

Unravelling dispersal history of members of the Elaeagnifolium clade will require more detailed phylogenetic sampling in the spiny solanums; the amphitropical pattern is found in other small groups in the larger Leptostemonum clade, and so is likely to have multiple origins. Wahlert et al. (2015) suggested that because the North American taxa were nested among a much larger clade of Neotropical species, mostly South American, that North American taxa originated from a South American progenitor. In the Elaeagnifolium clade (see Fig. 1 in Wahlert et al. 2014) it is clear that two distinct dispersal events have taken place, one to account for the two species groups (*S. hindsianum*+*S. houstonii* and *S. mortonii* + [*S. elaeagnifolium*+*S. homalospermum*]) and the other for the disjunct distribution of *S. elaeagnifolium* itself. This latter event may be more recent, such as has been observed for some species of *Tiquilia* (Moore et al. 2006b). Studies of chloroplast haplotypes suggest *S. elaeagnifolium* has a long history in southern South America (Chiarini et al. 2016) and is not a recent introduction.

Pollination and breeding systems. Like all *Solanum* species with poricidal anthers, pollen of members of the Elaeagnifolium clade can only be extracted by insects that can sonicate the anthers (Michener 1962; Linsley and Cazier 1963; Buchmann et al. 1977). In the majority of cases these are bees from a variety of families; they use their indirect flight muscles to set up vibrations in the anther cone that causes pollen to squirt out and be deposited on the ventral surface of the bee (Buchmann et al. 1977). Bee visitation to *S. elaeagnifolium* has been studied in its native North American range (Linsley and Cazier 1963) where a number of species of solitary bees (see Table 2) visit and buzz the flowers. In Arizona (Linsley 1962; Linsley and Cazier 1963, 1970; Buchmann and Cane 1989) bees of the genera *Ptiloglossa* and *Psaenythia* are *Solanum* specialists, and *Ptiloglossa* did most of its foraging in the hours just prior to sunrise (matinal behaviour), and forced flowers open to access the anthers (Linsley and Cazier 1963); visits to *S. elaeagnifolium* stopped when the sun reached flowers. Bumblebees of the genus *Bombus*, more generalist pollinators, visited throughout the day (Linsley and Cazier 1963). Jensen-Haarup (1908) and Jörgensen (1909) recorded a large number of bee species (see Table 2) visiting flowers of *S. elaeagnifolium*, but did not record times of day. The most specialist (“apparently preferring *Solanum elaeagnifolium*”) of the taxa observed were *Augochloropsis argentina*, *Psaenythia philanthoides* and females of the carpenter bee *Xylocopa brasilianorum* (Jensen-Haarup 1908). In Greece, where it is not native, *S. elaeagnifolium* competes for bee visits with native plants, significantly impacting their reproduction (Tscheulin and Petanidou 2013); here flowers are visited throughout the day. Carpenter (*Xylocopa* spp.) and bumble bees (*Bombus* spp.) have been reported as visiting *S. hindsianum* (https://www.desertmuseum.org/visit/sheets/Solhin.pdf).

**Table 2.** Bee visitors recorded on members of the Elaeagnifolium clade. Scientific names of bees are as recorded in the original publications, with more recent taxonomic placements (taken from Ascher and Pickering 2017) indicated in square brackets.

Species	Country	Bee Species	Reference
*Solanum elaeagnifolium*	Argentina (Prov. Mendoza, environs of Mendoza)	*Augochlora argentina* Friese (Halictidae) [=*Augochloropsis argentina* (Friese)] *Centris muralis* Burmeister (Centridae) *Psaenythia laticeps* Friese (Andrenidae) *Psaenythia philanthoides* Gerstäcker (Andrenidae) *Psaenythia picta* Gerstäcker (Andrenidae) *Xylocopa brasilianorum* (L.) (Apidae) *Xylocopa splendidula* Lepeletier (Apidae)	Jensen-Haarup 1908
*Solanum elaeagnifolium*	Argentina (Prov. Mendoza, environs of Mendoza)	*Augochlora argentina* Friese (Halictidae) [=*Augochloropsis argentina* (Friese)] *Centris brethesi* Schrottky (Centridae) *Psaenythia philanthoides* Gerstäcker (Andrenidae) *Psaenythia picta* Gerstäcker (Andrenidae) *Xylocopa brasilianorum* (L.) (Apidae) *Xylocopa splendidula* Lepeletier (Apidae)*Ancyloscelis nigriceps* Friese (Apidae) [=*Alepidosceles filitarsis* (Vachal)] *Anthophora saltensis* Holmberg (Apidae) [=*Anthophora paranensis* Holmberg] *Biglossa armata* Friese (Colletidae) [= *Lonchopria chalybaea* (Friese)] *Biglossa thoracica* Friese (Colletidae) [=*Lonchopria thoracica* (Friese)] *Colletes bicolor* Smith (Colletidae) *Colletes furfuraceus* Holmberg (Colletidae) *Tetralonia flavitarsis* Spinola (Apidae) [=*Svastra flavitarsis* (Spinola)] *Tetralonia jenseni* Friese (Apidae) [= *Alloscirtetica gilva* (Friese)]	Jörgensen 1909
*Solanum elaeagnifolium*	Argentina (Prov. San Luis ?)	*Exomalopsis jenseni* Friese (Apidae)	Petenatti and Del Vitto 1991
*Solanum elaeagnifolium*	Australia (greenhouse study, Adelaide)	*Amegilla chlorocyanea* Cockerell (Apidae)	Mark 2014
*Solanum elaeagnifolium*	Greece	*Bombus terrestris* L. (Apidae) *Xylocopa violacea* L. (Apidae) *Xylocopa iris* Christ (Apidae) *Pseudapis* sp. (Apidae) *Amegilla* spp. (Apidae)	Tscheulin and Petanidou 2013
*Solanum elaeagnifolium*	United States (Arizona, Cochise Co., near Portal, Chiricahua Mountains)	*Ptiloglossa arizonensis* Timberlake (Colletidae) *Bombus morrisoni* Cresson (Apidae) *Bombus sonorus* Say (Apidae)	Linsley 1962
*Solanum elaeagnifolium*	United States (Arizona, Cochise Co., near Douglas)	*Ptiloglossa jonesi* Timberlake (Colletidae) *Caupolicana yarrowi* (Cresson) (Colletidae) *Protoxaea gloriosa* (Fox) (Andrenidae) *Psaenythia mexicanorum* (Cockerell) (Andrenidae) [=*Protandrena mexicanorum* (Cockerell)] *Nomia mesillensis* Cockerell (Halictidae) [=*Nomia foxii* Dalla Torre)	Linsley and Cazier 1963
*Solanum elaeagnifolium*	United States (Arizona, Cochise Co., near Portal, Chiricahua Mountains)	*Ptiloglossa arizonensis* Timberlake (most important pollinator) (Colletidae) *Ptiloglossa jonesi* Timberlake (Colletidae)	Linsley and Cazier 1970
*Solanum elaeagnifolium*	United States (Arizona, Cochise Co., near Portal, Chiricahua Mountains)	*Ptiloglossa arizonensis* Timberlake (Colletidae) *Bombus sonorus* Say (Apidae)	Buchmann and Cane 1989
*Solanum hindsianum*	United States (Arizona, cultivated plants)	“great attractors of carpenter [*Xylocopa* spp.] and bumble bees [*Bombus* spp.], two of their pollinators”	Arizona Sonora Desert Museum Fact Sheet (https://www.desertmuseum.org/visit/sheets/Solhin.pdf)

The anthers of members of the Elaeagnifolium clade are not tightly connivent (pepper pot configuration of Glover et al. 2004), and so only large bees who can contact both style and anthers are likely to effect pollination; smaller bees such as halictids who visit single anthers do not contact stigmas and so are more correctly viewed as pollen thieves, as are honeybees, who glean pollen from petals and anthers (Buchmann and Cane 1989).

Andromonoecy, possession of hermaphroditic and functionally staminate flowers in a single inflorescence, has evolved multiple times in *Solanum* (Symon 1970; Symon 1979; Whalen and Costich 1989; Vorontsova et al. 2013; Aubriot et al. 2016). Members of the Elaeagnifolium clade range from weakly (e.g., *S. elaeagnifolium*) to strongly (e.g., *S. houstonii*) andromonoecious. In *S. elaeagnifolium*, most flowers are perfect with a few short-styled flowers distally in some plants, while *S. homalospermum*, *S. houstonii*, and *S. mortonii* have a single hermaphroditic basal flower with all the rest of the flowers in an inflorescence short-styled and functionally staminate. *Solanum hindsianum* appears to be somewhat intermediate between these states, with a few hermaphroditic flowers in each inflorescence. To date, the strength and plasticity of andromonoecy in the species of the group has not been assessed, but ongoing studies by one of us (A.K.Z. Carbonell) are aimed at elucidating breeding systems in the species of the Elaeagnifolium clade.

Conservation status. With the exception of *S. homalospermum* and *S. mortonii*, the members of the *S. elaeagnifolium* group are relatively widespread and not of immediate conservation concern. Their propensity to be common where they occur through vegetative reproduction, however, may mean that some taxa (e.g., *S. mortonii*) have limited genetic diversity. *Solanum houstonii*, while widespread, has considerable morphological diversity within its populations, suggesting a more populational rather species level approach to conservation would be beneficial. For preliminary conservation assessments of each species, see Table 3 and the individual species treatments below.

**Table 3.** Preliminary conservation status for members of the Elaeagnifolium clade. See Methods for details of threat status calculation, and species discussions for further discussion.

Species	IUCN threat status	Criteria (IUCN 2016)
*Solanum elaeagnifolium*	LC (Least Concern)	Widespread invasive
*Solanum hindsianum*	LC (Least Concern)	Widespread, but relatively local
*Solanum homalospermum*	EN (Endangered)/CR (Critically Endangered)	B1 (EOO <5,000 km^2^), B2 (AOO <100 km^2^), a (number of locations <5). See discussion under *S. homalospermum*.
*Solanum houstonii*	LC (Least Concern)	Widespread
*Solanum mortonii*	EN (Endangered)	B1 (EOO<5,000 km^2^), B2 (AOO <100 km^2^), a (number of locations <5).

### Species concepts

Our goal for the treatment of the species of this small clade has been to provide circumscriptions for the members of this morphologically variable group of species, while clearly highlighting those taxa and populations where further in-depth research would be useful. Delimitation of species here basically follows what is known as the “morphological cluster” species concept (Mallet 1995): i.e., “assemblages of individuals with morphological features in common and separate from other such assemblages by correlated morphological discontinuities in a number of features” (Davis and Heywood 1963). Biological (Mayr 1982), phylogenetic (Cracraft 1989) and the host of other finely defined species concepts (see Mallet 1995) are almost impossible to apply in practice and are therefore of little utility in a practical sense. It is important, however, to clearly state the criteria for the delimitation of species, rather than dogmatically follow particular ideological lines (see Luckow 1995; Davis 1997). Our decisions relied on clear morphological discontinuities to define the easily distinguished species. Specific characters used for recognition are detailed with each species description and in the key. Some potential reasons for variability and intergradation are recent divergence, hybridization and environmental influences on morphology. In this revision we have tried to emphasise similarities between populations instead of differences, which so often reflect incomplete collecting or local variation. We have not recognised subspecies or varieties, but have rather described and documented variation where present, rather than formalised such variability with a name which then encumbers the literature. We have been conservative in our approach, recognising as distinct entities those population systems (sets of specimens) that differ in several morphological characteristics. All of the species in the clade are extremely widespread and variable; variation exists in certain characters, but the pattern of variation is such that no reliable units can be consistently extracted, nor is geography a completely reliable predictor of character states. Here variability within and between populations seems more important than the variations of the extremes other taxonomists have recognised as distinct. We describe this variation realising that others may wish to interpret it differently.

## Materials and methods

This monograph is based on examination of herbarium specimens supplemented with field observations in North and South America. We examined approximately 2,100 collections (ca. 2,700 specimens) from the following herbaria (herbarium acronyms follow *Index Herbariorum*, found on-line at http://sweetgum.nybg.org/science/ih/): A, AD, ARIZ, AS, B, BA, BAB, BACP, BH, BM, BRY, C, CAS, CICY, CIQR, CORD, CTES, DS, DUKE, E, EA, EIU, ENCB, ENLC, ESA, F, FCQ, G, G-DC, GH, GOET, K, LE, LIL, MA, MERL, MESA, MEXU, MICH, MISS, MMNS, MO, NA, NU, NY, P, RENO, RM, RSA, S, SF, SI, UC, UNM, US, USM, UT, UTC, VT, W, XAL, Z. We cited specimens from online sources only when we could see an image and confirm the identification.

Measurements were made from dried herbarium material supplemented by measurements from living material. Colours of corollas, fruits, etc., are described from living material or from herbarium label data. Specimens with latitude and longitude data on the labels were mapped directly. Some species had few or no georeferenced collections; in these cases, we retrospectively georeferenced the collections using available locality data. The extremely widespread *S. elaeagnifolium* was mapped based on GBIF records (http://www.gbif.org/species/2929892; 3,032 georeferenced observations on 6 November 2016) and specimens we have seen. Maps were constructed with the points in the centres of degree squares in a 1° square grid. Conservation threat status was assessed following the IUCN Red List Categories and Criteria (IUCN 2014) using the GIS-based method of Moat (2007) as implemented in the online assessment tools in GeoCat (http://geocat.kew.org). The Extent of Occurrence (EOO) measures the range of the species, and the Area of Occupancy (AOO) represents the number of occupied points within that range based on the default grid size of 2 km^2^.

Where specific herbaria have not been cited in protologues we have followed McNeill (2014) and designated lectotypes rather than assuming holotypes exist. We cite page numbers for all previous lectotypifications.

Type specimens with sheet numbers are cited with the herbarium acronym followed by a dash and the sheet number (i.e., MO–1781232); barcodes are written as a continuous string (i.e., G00104280). We have cited geographically representative specimens for taxa where more than 100 collections are known. Full specimen details are available on the Solanaceae Source website (www.solanaceaesource.org) and in the dataset for this study deposited in the Natural History Museum Data Portal (http://dx.doi.org/10.5519/0007624). Specimens cited in the text are listed by country alphabetically, rather than geographically; within any country major political divisions are also listed in alphabetical order.

Citation of literature follows BPH-2 (Bridson 2004) with alterations implemented in IPNI (International Plant Names Index, http://www.ipni.org) and Harvard University Index of Botanical Publications (http://kiki.huh.harvard.edu/databases/publication_index.html). Following Knapp (2013a) we have used the square bracket convention for publications in which a species is described by one author in a publication edited or compiled by another. These citations are the traditional “in” attributions such as Dunal in DC. for those taxa described by Dunal in Candolle’s *Prodromus Systematis Naturalis Regni Vegetabilis*. This work is cited here as Prodr. [A.P. de Candolle] and the names are thus attributed only to Dunal. For “ex” attributions we cite only the publishing author, as suggested in the *Code* (McNeill et al. 2012). Standard forms of author names are according to IPNI (International Plant Names Index, http://www.ipni.org).

## Taxonomic treatment

### The Elaeagnifolium clade of Solanum

Taxon classificationPlantaeSolanalesSolanaceae

, sensu Stern et al. (2011) and Wahlert et al. (2014)

Solanum section Leprophora Dunal, Hist. Solanum 125, 181. 1813. Type species. S. elaeagnifolium Cav.
Solanum ellipticum species group sensu Whalen (1989), S. elaeagnifolium only, excl. type.
Solanum vespertilio group sensu Whalen (1989), S. houstonii only [as S. tridynamum], excl. type.
Solanum section Lathyrocarpum G.Don, Series 2 Nee (1999), S. mortonii Hunz. only, excl. type.
Solanum section Lathyrocarpum G.Don, Series 4 Nee (1999). Type species: S. elaeagnifolium Cav. (incl. also S. hindsianum, S. houstonii [as S. tridynamum]).

#### Description.

Shrubs, sometimes rhizomatous, armed or unarmed. Stems terete, pubescent with multangulate, porrect-stellate or lepidote trichomes, sometimes glabrescent. Sympodial units difoliate or occasionally trifoliate or plurifoliate, not geminate. Leaves simple to shallowly lobed to occasionally somewhat pinnatifid, concolorous or discolorous, densely pubescent with multangulate, stellate or lepidote trichomes; petioles well developed, sometimes channelled above. Inflorescences terminal to lateral, usually unbranched, occasionally furcate, not bracteate, with up to 10 flowers (exceptionally to 26 flowers in *S. houstonii*), in andromonoecious plants with a single (or two) hermaphroditic long-styled flowers at the base and all distal flowers short-styled and functionally staminate; peduncle robust, clearly distinct armed or unarmed; pedicels articulated at the base, armed or unarmed. Flowers 5-merous, actinomorphic to zygomorphic, perfect or strongly heteromorphic with long- and short-styled morphs and the plants andromonoecious. Calyx armed or unarmed, the lobes deltate and usually strongly keeled with an elongate acumen. Corolla stellate or rotate stellate, purple or occasionally white, usually with a green star at the base, the lobes spreading or slightly reflexed at anthesis. Stamens unequal, sometimes markedly so (*S. houstonii*), the filaments equal, the anthers strongly tapering with distally directed pores, usually somewhat spreading and not connivent. Ovary conical, vestigial in strongly andromonoecious species, glabrous or sparsely stellate-pubescent; style in long-styled flowers straight or curved, glabrous or sparsely stellate-pubescent near the base; stigma clavate in long-styled flowers, vestigial in short-styled flowers. Fruit a globose berry, indehiscent or dehiscent, often enclosed in the accrescent calyx; pericarp brittle at fruit maturity, glabrous. Seeds flattened reniform, yellowish tan or blackish brown, often shiny with what appears to be a sticky substance. Chromosome number: n=12, 24, 36 (Moscone 1992; Acosta et al. 2005; Scaldaferro et al. 2012; Chiarini et al. in press).

#### Distribution.

An exclusively New World group occurring in North America (western United States and Mexico) and southern South America (Argentina, Paraguay, Brazil, Uruguay, Chile). One species, *S. elaeagnifolium*, is an invasive weed in dry areas worldwide.

#### Discussion.

As discussed above under Phylogeny and as can be seen by the synonymy of the clade, members of this group were previously not thought to be closely related. Their resolution as sister to the diverse and diversifying Old World clade of spiny solanums makes them of particular interest in terms of character evolution. Nee (1999) placed all of the species treated here in his section Lathyrocarpum (whose type species is *S. carolinense* L., see Wahlert et al. 2015) in two different series; in this he followed the views of Hunziker (1979).

The clade, as is common with the groups of spiny solanums, has few unambiguous and unique synapomorphies. The andromonoecious habit (very weak in *S. elaeagnifolium*), unusual dry and often dehiscent berries, dark sticky seeds, silvery pubescence and propensity to grow in arid zones are all characters that are shared by the species in this group. None of these are unique to the *S. elaeagnifolium* group, however, either in *Solanum* or in Solanaceae more widely, although the dehiscent berries of all these species (save *S. elaeagnifolium*) are only rarely found in *Solanum* (e.g., *S. tununduggae*, *S. vansittartensis* of Australia, see Symon 1981; Knapp 2002a).

#### Artificial key to the species of the *S. elaeagnifolium* group

**Table:** 

1	Pubescence of leaves lepidote, the trichomes with the rays fused at the base; prickles, if present, often orange; flowers mostly all perfect; stamens equal or nearly so; fruits more than 1 per infructescence	**1. *Solanum elaeagnifolium***
–	Pubescence of leaves stellate or multangulate, the trichomes with the rays not fused or if very slightly fused near the base; prickles, if present, brown or yellow; flowers heteromorphic, not all perfect; stamens unequal; fruits usually 1 per infructescence.	**2**
2	Trichomes of stems and leaves multangulate, the rays in multiple planes; Argentina	**5. *Solanum mortonii***
–	Trichomes of stems and leaves stellate, the rays in a single spreading plane (porrect-stellate)	**3**
3	Pedicels strongly deflexed in fruit; trichome rays always fewer than 10; midpoint equal in length to the lateral rays; flowers white; Argentina	**3. *Solanum homalospermum***
–	Pedicels erect in fruit; trichome rays usually more than 10; midpoint shorter than the lateral rays; flowers purple or lavender (occasionally white); Mexico	**4**
4	Flowers weakly heteromorphic, the anthers in hermaphroditic and staminate flowers not different, straight or only slightly curved; fruiting calyx not or only sparsely prickly, usually not covering the berry by more than 2/3 of its length; Sonoran Desert	**2. *Solanum hindsianum***
–	Flowers strongly heteromorphic, the hermaphroditic flower with the anthers more or less equal in size, the staminate flowers with 3 anthers much longer than the rest and strongly curved; fruiting calyx prickly, accrescent and almost completely enclosing the berry; widespread in Mexico	**4. *Solanum houstonii***

### Species descriptions

#### 1. Solanum elaeagnifolium

Taxon classificationPlantaeSolanalesSolanaceae

Cav., Icon. 3: 22, tab. 243. 1795.

Figures 2A , 3A, B , 4A , 5

Solanum leprosum Ortega, Nov. Rar. Pl. Hort. Matrit. Dec. 9: 115. 1800. Type. Cultivated in Real Jardin Botánico de Madrid, seeds sent by L. Née, *Without collector s.n.* (neotype, designated by Knapp 2013b, pg. 59: MA [MA-334600]; possible isoneotypes: MA [MA-334600/2, MA-334600/3]).
Solanum obtusifolium Dunal, Solan. Syn. 26. 1816. Type. Mexico. Hidalgo: “prope Regla et Totonilco [Atotonilco] el Grande, 1200 hex”, *A. Humboldt & A. Bonpland s.n.* (holotype: P-Bonpl. [P001136347]).
Solanum saponaceum Hook., Bot. Mag. 53: tab. 2697. 1826, nom. illeg., non S. saponaceum Dunal, 1813. Type. Argentina. Mendoza: near Río Saladillo, 16 Nov 1820, J. Gillies Solanum-2 (lectotype, designated by Vorontsova and Knapp 2016, pg. 150: K [K000546062]).
Solanum flavidum Torr., Ann. Lyceum Nat. Hist. New York 2: 227. 1828 Type. United States of America. “from Mississippi River to Rocky Mtns.”, summer 1820, *E. James s.n.* (lectotype, designated by Vorontsova and Knapp 2016, pg. 150: NY [NY00759814]).
Solanum dealbatum Lindl., Trans. Hortic. Soc. 7: 52. 1830. Type. Chile. Cumbre “Andium Claustrum”, 1826, *J. McRae s.n.* (lectotype, designated by Vorontsova and Knapp 2016, pg. 151: K [K000546063]).
Solanum texense Engelm. & A.Gray, Boston J. Nat. Hist. 5: 227. 1845. Type. United States of America. Texas: Brazos [River], Jun 1843, *F. Lindheimer [66] 135 [fasc. I 1843*] (lectotype, designated by Vorontsova and Knapp 2016, p. 151: MO [sheet number MO-3847597]; isolectotypes: BM [BM000514925], GH [GH00217439], LE [2 sheets], MO [sheet number MO-360819]).
Solanum roemerianum Scheele, Linnaea 21: 767. 1848. Type. United States of America. Texas: Travis County, Austin, Apr, *F. Roemer s.n.* (no herbarium cited, HAL?; possible type material: BM [BM000942973]).
Solanum elaeagnifolium Cav. var. leprosum (Ortega) Dunal, Prodr. [A.P. de Candolle] 13(1): 291. 1852. Type. Based on Solanum leprosum Ortega.
Solanum elaeagnifolium Cav. var. obtusifolium (Dunal) Dunal, Prodr. [A.P. de Candolle] 13(1): 291. 1852. Type. Based on Solanum obtusifolium Dunal.
Solanum elaeagnifolium Cav. var. grandiflorum Griseb., Abh. Königl. Ges. Wiss. Göttingen 24: 255. 1879. Type. Argentina. Catamarca: Sin. loc., Nov 1872, *P. Lorentz & G. Hieronymus 1243* (holotype: GOET [GOET003503]; isotypes: CORD [CORD00006119], GOET [GOET003504]).
Solanum elaeagnifolium Cav. var. argyrocroton Griseb., Abh. Königl. Ges. Wiss. Göttingen 24: 255. 1879. Type. Argentina. Tucumán: between La Oiada and border of provincias Tucumán and Salta, Feb 1873, *P. Lorentz & G. Hieronymus 533* (holotype: GOET [GOET003502]; isotype: CORD [CORD00006120]).
Solanum elaeagnifolium Cav. var. albiflorum Cockerell, Bull. Torrey Bot. Club 20: 410. 1893. Type. U.S.A. Texas: El Paso County, El Paso, 1893, *T. Cockerell s.n.* (lectotype, designated here: NY [NY00759810]).
Solanum elaeagnifolium Cav. var. angustifolium Kuntze, Revis. Gen. Pl. 3(2): 225. 1898. Type. Argentina. Santiago del Estero: San Rafael, *O. Kuntze s.n.* (lectotype, designated by Vorontsova and Knapp 2016, pg. 151: NY [NY00139141]).
Solanum elaeagnifolium Cav. var. benkei Standl., Rhodora 34: 176. 1932. Type. United States of America. Texas: Cameron County, Brownsville, near mouth of Rio Grande, 20 Mar 1930, *H. Benke 5209* (holotype: F [F-642429, F neg. 49341]).

##### Type.

Cultivated in Madrid from “America calidiore” [“del viaje de los españoles alrededor del mundo, Cult. en el R. J. Bot. 1793”], *Anon. s.n.* (lectotype, designated by Knapp 2007, pg. 198: MA [MA-476348-2]; isolectotype: MA [MA-476348-1).

##### Description.

Herbaceous, woody at base, 20 to 50 cm tall. Stems erect, with running underground stems, sparsely or densely armed, sometimes unarmed; young stems densely pubescent with stellate, sessile to subsessile, porrect lepidote trichomes, the stalk 0.1–0.15 mm long, the rays 10–14(16), 0.1–0.3 mm long, fused at the base, the midpoints 0.1–0.15 mm long, usually absent, unarmed or prickly with the prickles, if present, usually uniform throughout the plant, 2–6 mm long if dense, 0.3–2 mm long if sparse, 0.5–2 mm wide at base, usually straight, orange to brick-red; bark of older stems smooth, brown to grey, the leaf scars small brown stumps. Sympodial units difoliate, not geminate. Leaves simple or shallowly lobed, (1.5)3–6(12) cm long, (0.3)1–2(3) cm wide, (2)3–4(6) times longer than wide, elliptic, sometimes lanceolate or ovate, mostly concolorous, drying yellow-green or silvery green, densely pubescent on both surfaces with sessile to subsessile lepidote trichomes, the rays (9)12–14, 0.1–0.3 mm long, porrect, fused at the base, the midpoint up to 0.15 mm long, sometimes reduced; principal veins 4–7 pairs, raised adaxially, spreading at ca. 30–45° from the midvein, the finer venation mostly not visible to the naked eye; base obtuse to rounded or cuneate, often oblique; margins entire to shallowly lobed, the lobes 4 to 6 on each side, the sinus extending up to ½ of the distance to the midvein, to 2(4) cm long, rounded to deltate; apex obtuse to rounded, rarely acute; petiole 0.2–2(4) cm, 1/2–1/6 of the leaf length, densely stellate-pubescent like the young stems. Inflorescences terminal or lateral, (2)3–5(6) cm long, with (3)4–6(7) flowers, unbranched (simple), densely pubescent with lepidote trichomes like the stems; peduncle 0.8–2.5 cm long; rachis 0.5–3 cm long; pedicels 1–3 cm long, ca. 1 mm in diameter, filiform or apically dilated, densely stellate-lepidote-pubescent like the young stems, usually armed, articulated very near the base; pedicel scars brown, spaced 0.5–1 mm apart, sometimes obscured by the dense pubescence. Buds ellipsoid, the calyx ca. 1/3 of the corolla length prior to anthesis. Flowers 5-merous, usually all perfect, but some distal ones short-styled, functionally staminate and the plants weakly andromonoecious. Calyx tube 2–3 mm long, conical or cup-shaped, the lobes 2- 4 mm long, 1–2 mm wide at base, cuspidate, venation not visible, densely lepidote-pubescent like the leaf blades, sparsely prickly or unarmed, if present the prickles to 2 mm long. Corolla 2.5–3(4) cm in diameter, purple to pale violet or white, stellate, lobed ca. 1/2 of the way to the base, the lobes 0.6–1(1.2) cm, 0.6–1 cm wide, deltate, reflexed to spreading at anthesis, often with a yellow midvein, stellate-pubescent abaxially, mostly glabrous adaxially, the trichomes variously irregular and reduced-multangulate. Stamens equal or slightly unequal in size, with the 2 adaxial anthers very slightly shorter than the 3 abaxial anthers; filament tube 0.8–1.5 mm; free portion of the filaments 1–2 mm; anthers 6–10(12) mm long, 1.5–2 mm wide, tapering, spreading or coherent, the surfaces often papillate, poricidal at the tips, the pores not lengthening with age. Ovary globose to ovoid, usually densely stellate-pubescent like the young stems, sometimes glabrous; style 7–15 mm long, glabrous, slightly dilated toward the apex, in rare short-styled flowers 2–4 mm long; stigma clavate, papillose. Fruit a globose berry, 1–5 per infructescence, 0.6–1.2 cm in diameter, the pericarp thin, smooth, glabrous, yellow to orange at maturity, drying orange to brown and cracking while still on the plant and the seeds released as a mass; fruiting calyx not markedly accrescent, the lobes reflexed or very occasionally covering up to 2/3 of the mature fruit, with prickles ca. 2 mm long; fruiting pedicels 1–2.5 cm long, 0.5–1(2) mm in diameter at the base, 1–2(3)mm in diameter at the apex, somewhat woody, strongly deflexed, usually armed. Seeds 20–60 per berry, 2–3 mm long, 2–3 mm wide, flattened reniform, orange-brown, the surfaces shiny, usually borne in a mass in the berry, the testal cells sinuate (not differing in size between ploidy levels, Nilda Dottori, pers. comm.). Chromosome number: n=12, 24, 36 (Moscone 1992; Powell and Weedin 2005; Scaldaferro et al. 2012); 2n=24 (Acosta et al. 2005).

##### Distribution

**Figure 6.**
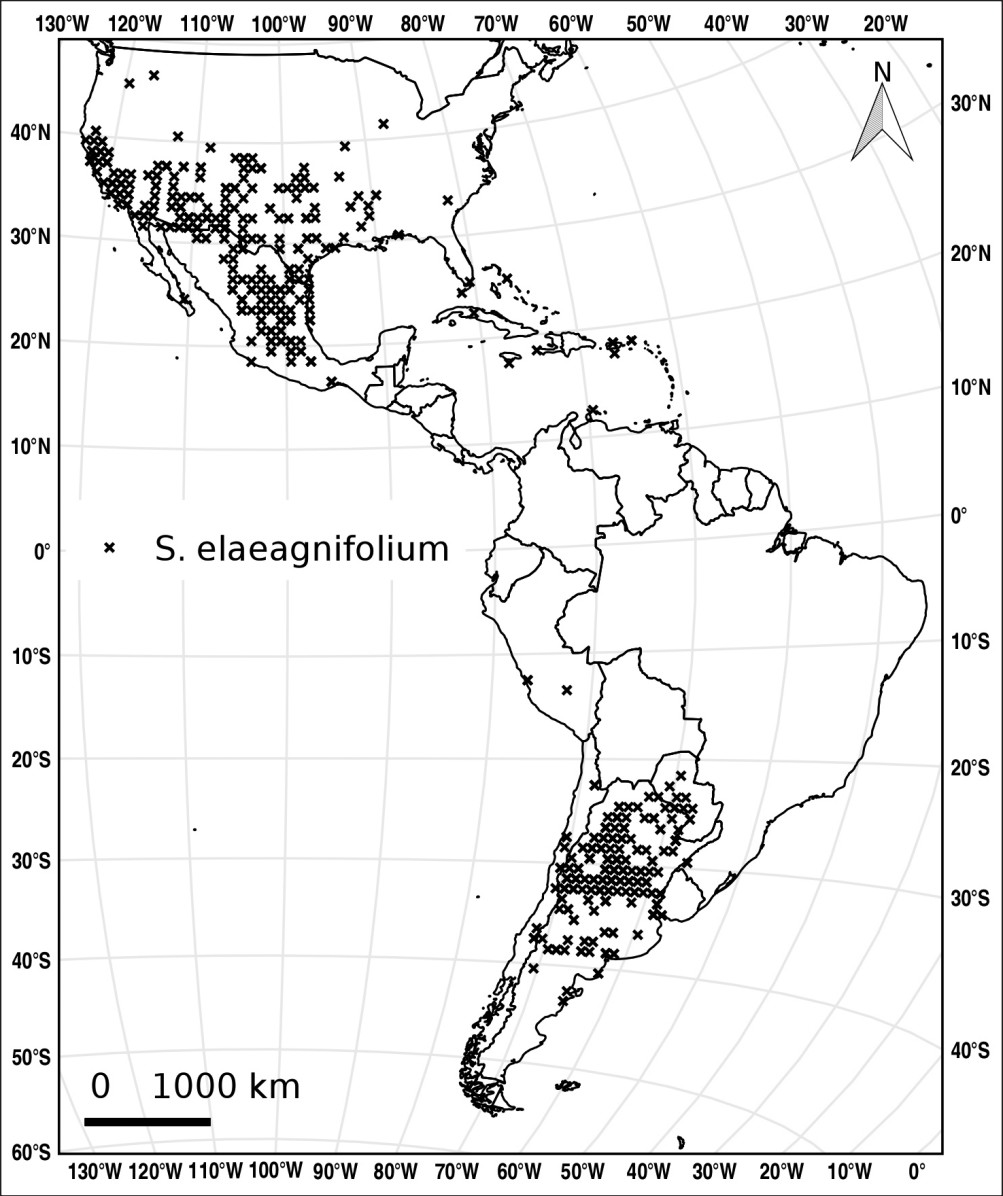
Distribution of *S. elaeagnifolium*.

(Figure 6, showing American distribution only). *Solanum elaeagnifolium* has an amphitropical native distribution, occurring in the deserts and dry zones of the northern hemisphere in the southwestern United States of America and Mexico and in the southern hemisphere in Argentina, Paraguay, Uruguay and Chile, but this species is widespread and invasive in tropical and subtropical regions worldwide (see below). We have seen (non-cultivated) specimens of invasive *S. elaeagnifolium* from outside its native range from Australia, Cyprus, Egypt, Greece, India, Iraq, Jordan, Kuwait, Malta, Morocco, Pakistan, Saudi Arabia, and South Africa.

##### Ecology and habitat.

*Solanum elaeagnifolium* is a plant of disturbed and open places, and often grows in full sun. It occurs at a very wide range of elevations from (-35 m below sea level, Salton Sink, California; *Goeden & Ricker s.n.*) sea level to 3200m. *Solanum elaeagnifolium* spreads quickly from underground stems and has become a pest of grazed land in Australia and in the Mediterranean. It is considered a noxious weed in 21 states in the United States (http://www.invasive.org) and in many other countries such as Australia, Canada, Egypt, Greece, India, Syria, Israel, Italy, South Africa and Zimbabwe (Feuerherdt 2010; Uludag et al. 2016; Invasive Species South Africa 2017). It is toxic to livestock and very hard to control, as root stocks less than 1 cm long can regenerate into plants (Cuthertson 1976; Fernández and Brevedan 1972; Stanton et al. 2011).

##### Common names and uses.

Argentina: capiquí, meloncillo de campo, meloncillo de olor, quillo, suriñado, tutiá chico, granadillo, tomatillo, pocotillo, revienta caballos (Chiarini 2013). Mexico: trompillo, buena mujer, pera, tomatito de buena mujer (http://www.conabio.gob.mx/malezasdemexico). United States of America: silverleaf nightshade, tomato weed, white horsenettle, trompillo, white nightshade.

In areas where *S. elaeagnifolium* is invasive it is usually referred to as silverleaf nightshade (Boyd et al. 1984 Hoffmann et al. 1998; Wu et al. 2016), but in South Africa it is also known as silverleaf bitter apple (English), satansbos, bloubos and silwerblaarbitterappel (Afrikaans) (Henderson 2011; Invasive Species South Africa 2017).

The berries of *S. elaeagnifolium* were used in the southwestern United States by Pima, Navajo and Hopi people for making cheese and for tanning leather (Boyd et al. 1984; Foxx and Hoard 1984) and in local communities in the state of Chihuahua (Mexico) for making filata type asadero cheeses; more recently berries been investigated chemically for use in the food industry due to their milk clotting properties (Gutiérrez-Méndez et al. 2012). In Argentina, the berries are used as soap due to their high saponin content (Chiarini 2013).

##### Preliminary conservation status

(IUCN 2016). LC (Least Concern). EOO = 298,812,730 km^2^ (LC - Least Concern); AOO = 2,420 km^2^ (NT – Near Threatened). *Solanum elaeagnifolium* is an invasive weed (see above) and is actively being eradicated in areas outside its native distribution. The low value for AOO is likely due to the low number of georeferenced collections.

##### Discussion.

*Solanum elaeagnifolium* is the most widespread and variable of the species of this group. It can be easily distinguished from the rest of the species by its dense silvery lepidote pubescence, usually orange prickles (if present), mostly perfect flowers that are only slightly zygomorphic in the androecium and multiple berries per infructescence that are exposed at maturity. It is sympatric with *S. hindsianum* and *S. houstonii* in Mexico, and with *S. homalospermum* and *S. mortonii* in Argentina. Fruit type and pubescence are the most reliable characters for separating the taxa in both North and South America, but in addition, the leaf bases of *S. elaeagnifolium* are usually more decurrent onto the petiole than any of the other four species.

*Solanum elaeagnifolium* is extremely variable in its degree of prickliness; this extreme polymorphism also occurs in *S. bahamense* L. of the Caribbean (Strickland-Constable et al. 2000). Plants are densely prickly (type of *S. elaeagnifolium*) to completely unarmed (type of *S. leprosum*), and sometimes individual branches on a single plant differ in degree of prickliness (e.g., *Bartholomew et al. 2430*, MEXU).

Populations of *S. elaeagnifolium* in North America are diploid, with n=12 (Powell and Weedin 2005; Chiarini et al. 2016), while those in Argentina are diploid, tetraploid or hexaploid (Scaldaferro et al. 2012). Morphological differentiation between ploidy levels has not been found (Nilda Dottori, pers. comm.), but hexaploid populations appear to possess a number of differences in plastid haplotypes. Chiarini et al. (2016) suggest this might indicate presence of a cryptic species, but data from the entire genome is needed to test this hypothesis. Tetraploids appear to have arisen multiple times from either diploid or hexaploid populations (Chiarini et al. 2016). All plants tested outside of the native range (e.g., South Africa, Australia, the Mediterranean) are diploid, and in Australia, genetic variation as measured using AFLP markers suggests that populations in southern Australia are the result of several introductions (Zhu et al. 2013a).

As discussed above in the section on Habitats and distribution it was long assumed that *S. elaeagnifolium* was native to North America and was introduced, perhaps by the Spanish or Portuguese, to southern South America (Goeden 1971; Boyd et al. 1984; Wapshere 1988; OEPP/EPPO 2007). The clustering of all accessions of *S. elaeagnifolium*, regardless of origin, as sister to the South American *S. mortonii*+*S. homalospermum* (Wahlert et al. 2014) shows this is not the case. Haplotypes based on plastid sequences cluster all diploids in a group with tetraploids in a single cluster and hexaploids in another (Chiarini et al. 2016).

Introduction of *S. elaeagnifolium* outside of its native range has resulted in its becoming significant weed of cultivation and pasture (see summaries in Feuerhdert 2010; Uludag et al. 2016). In Australia it was introduced as a contaminant of grain and fodder in the early part of the 20^th^ century (Parsons and Cuthbertson 2001), but did not become a problem until the 1960s (Stanton et al. 2012). The species became a problem weed in South Africa in the 1970s (Olckers et al. 1999); our earliest record of it from that country is from 1919 (*Carnegie 40*). In the Mediterranean it has gone from a few accidental introductions, for example with cotton in Morocco (Bouhache 2010), to a weed necessitating control in many countries (Uludag et al. 2016). Plants from botanic gardens such as those used to describe *S. elaeagnifolium* and *S. leprosum* appear not to have escaped, and the weedy populations are all thought to be derived from accidental introductions associated with agriculture and subsequent mechanical disturbance of the soil. The Mediterranean populations have been shown to be most genetically similar to those from Texas (Suskiuw 2010). Our records from herbaria indicate that *S. elaeagnifolium* occurs in India, Iraq, Jordan, Kuwait and Saudi Arabia (see http://dx.doi.org/10.5519/0007624 for complete specimen citations) in addition to in those areas such as Australia, South Africa and the Mediterranean where it is already a listed pest. A worldwide genetic similarity study will be necessary to document and track introduction and invasion of *S. elaeagnifolium*; studies on diversity of insect pests as indicators of origins (e.g., Goedin 1971) will need to be tested using new genetic tools. It will also be important to take into account new phylogenetic results (e.g., Wahlert et al. 2014) in determining where invasive populations have originated.

Measures for the control of *S. elaeagnifolium* have involved both chemical and biological agents. Herbicide control has been used in Australia and in the Mediterranean (Feuerherdt 2010; Uludag et al. 2016; Wu et al. 2016), while biological control using herbivorous insects from the native range in North America (Goeden 1971) has been successful in South Africa (Hoffmann et al. 1998; Olckers et al. 1999). In South Africa, the chrysomelid beetle *Leptinotarsa texana* Schaeffer, a relative of the Colorado potato beetle (*Leptinotarsa decemlineata* Say), strips *S. elaeagnifolium* plants and seriously compromises reproductive potential (Hoffmann et al. 1998); this species is being considered as a biological control agent in Australia (Wu et al. 2016). Other insects released to help control *S. elaeagnifolium* remained at low densities, with no impact; one of these biological control agents has been recorded to have impacted native *Solanum* species in South Africa (Olckers et al. 1998). Because *S. elaeagnifolium* reproduces both from seed and vegetatively, the importance of control of both seed and root stocks has been emphasized (Wu et al. 2016). The great morphological (Zhu et al. 2013a) and genetic (Zhu et al. 2013b, c) variability of *S. elaeagnifolium* surely contributes to its success as an invasive weed. Combining results from phylogenetics and biogeography with those from control measures will surely be important for future control and management of this species as it is introduced elsewhere.

Lectotypes for most of the synonyms of *S. elaeagnifolium* were designated by Vorontsova and Knapp (2016). At that time, we had not found any original material relating to S. elaeagnifolium forma albiflorum, but a tiny scrap of plant in NY (NY00759810) is annotated as “f. albiflorum” and bears the correct locality. We designate this as the lectotype here.

##### Selected specimens examined

**(American range only). Argentina**. **Buenos Aires**: Rivadavia, F.C.C.A, 3 Mar 1945, *Álvarez 573* (W); Hurlingham, F.C.S, 16 Mar 1945, *Álvarez 631* (NY, SI); Tornquist, Sierra de La Ventana, desvío por camino de tierra rumbo a La Hoya (unos 70 m del Camping de Sierra de la Ventana, bordeando el río, 22 Jan 2010, *Barboza et al. 2308* (CORD, CTES, SI); Bahía Blanca, Bahía Blanca, en los alrededores de la ciudad, en baldíos sobre Avda. Colón al 2000, 23 Jan 2010, *Barboza et al. 2319* (CORD, CTES, SI); La Plata, Dec 1944, *Boffa s.n.* (NY, SI); San Miguel, Bella Vista, Feb 1946, *Castellanos 806* (CORD, LIL); Campana, 27 Nov 1938, *Eyerdam* & *Beetle 23093* (BH, K); Coronel Dorrego, Sauce Chico Río, 120 km east of Bahía Blanca on road to Tres Arroyos, 13 Dec 1938, *Eyerdam et al. 23735* (K, SI); Buenos Aires en la calle Nazca, 9 Feb 1943, *Hunziker 3252* (CORD); Tigre, cerca del Río las Conchas, 25 Jan 1945, *Hunziker 280* (CORD); Patagones, Carmen de Patagones, 13 Apr 1945, *Hunziker 347* (CORD); Tigre, 24 Dec 1943, *Lanfranchi 35* (SI); Martín Coronado, 12 Jan 1939, *Nicora, E.G. 2101* (SI); Guaminí, Isla Chica, Laguna del Monte, 19 Jan 1946, *Nicora 4162* (SI); Rincón Viedma, Dec 1933, *Ringuelet, E.J. 255* (SI); Pergamino, 4 Dec 1944, *Schulz 5590* (W); Bahía Blanca, camino a Cuatreros (km 700), San Fernando, Jan 1902, *Stuckert 11590* (CORD); barrancas al sur de Buenos Aires, 12 Mar 1902, *Venturi 63* (CORD, SI). **Catamarca**: Santa Maria, Santa María, 23 Nov 1949, *Araque M.* & *Barkley 19Ar*-*341* (K); La Paz, La Guardia, 10 Feb 2012, *Barboza et al. 3435* (BM, CORD); La Paz, km 981 F. C, 4 Apr 1950, *Brizuela 1159* (CORD, LIL); Andalgalá, 4 km N of Andalgalá, on roadside, 15 Dec 1972, *Cantino 505* (CORD, SI); Capayán, Ruta 60 (km 1044), entre El Médano y San Martín, 11 Feb 1988, *Hunziker 25228* (CORD); Tinogasta, El Cordoncito, 26 Feb 1950, *Hunziker* & *Caso 4097* (BAB, CORD); Santa María, El Medanito, 19 Feb 1948, *Reales 952* (CORD, LIL); Santa María, El Rasguñado, 3 Feb 1949, *Reales 1615* (CORD, LIL); Santa María, Famatanca, 15 Feb 1949, *Reales, A. 1678* (CORD, LIL); Andalgalá, Fuerte de Andalgalá, Oct 1875, *Schickendantz 82* (CORD); Poman, Dec 1909, *Spegazzini 28174* (SI); La Paz, Casa de Piedra: km 1103, 28 Feb 1986, *Subils 3881* (CORD); Del Alto, Balcogna, 18 Jan 1928, *Venturi 7132* (CAS); Santa Maria, La Puntilla, 12 Apr 1947, *Villafañe 1265* (W); Santa María, Fuerte Quemado, 28 Apr 1947, *Villafañe 1271* (E). **Chaco**: Resistencia, Margarita Belén, 13 Oct 1946, *Aguilar 912* (W); General Güemes, 5 km N Castelli (Colonia Fortuny), 24 Aug 1973, *Bordón s.n.* (CTES); 1 de Mayo, al costado de la RN 11, entre Resistencia y Colonia Benítez, 4 Mar 2012, *Chiarini* & *Wahlert 879* (CORD); Independencia, Baldecito, 15 Jan 1907, *Kurtz 14206* (CORD); General Güemes, Fortín Lavalle, 24 Jan 2006, *Martínez, G.J. 486* (CORD); General Güemes, Río Bermejo, 27 Jan 2006, *Martínez 524* (CORD); Vientecinco de Mayo, Machagay, 8 May 1945, *Meyer 9863* (K). **Chubut**: Biedma, Puerto Madryn, saliendo de la ciudad rumbo a Trelew, a 4 km de P. Madryn sobre Ruta 3, 24 Jan 2010, *Barboza et al. 2334* (CORD, CTES, SI); Rawson, km 54, en las proximidades de Trelew, 24 Jan 2010, *Barboza et al. 2340* (CORD, CTES, SI). **Córdoba**: Colón, Estancia Santo Domingo, por el camino de tierra paralelo al camino de la entrada a la estancia, antes de llegar a los maizales, 14 Mar 2005, *Ariza Espinar et al. 3587* (CORD); San Alberto, desde San Carlos Minas, rumbo a Córdoba, por RP 15, 1 Feb 2008, *Barboza et al. 2010* (CORD); Tulumba, Salinas Grandes, ca. 50 km al norte de Quilino, antes de llegar al límite con Catamarca, 10 Feb 2012, *Barboza et al. 3434* (BM, CORD); Minas, La Playa, 26 Feb 2014, *Barboza* & *Palchetti 4120* (CORD); San Justo, Laguna del Plata, 16 Feb 2015, *Barboza et al. 4337* (CORD); Gutenberg, 24 Mar 1943, *Bartlett 19871* (SI); Tulumba, Cerro Colorado, 4 Mar 1984, *Bernardello 449* (CORD); Sobremonte, San Francisco del Chañar, unos 6 km al oeste de la plaza del pueblo, yendo por el camino hacia L.V. Mansilla, 13 Jan 1986, *Bernardello 534* (CORD); Colón, Caroya, 15 Jan 1950, *Borsini 1201* (CORD); Altautina, 29 Dec 2009, *Cantero* & *Núñez 6183* (CORD); Calamuchita, Valle de Los Reartes, 8 Feb 1919, *Castellanos 90* (SI); Colón, La Calera, 28 Jan 1999, *Chiarini 40* (CORD); Capital, Ciudad de Córdoba, calle Ovidio Lagos esquina Libertad, 13 May 2001, *Chiarini 462* (CORD); Tulumba, sobre Ruta 60 rumbo a Totoralejos, 28 Feb 2002, *Chiarini et al. 559* (CORD); Pocho, Los Crestones, cerca de la estancia Las Gramillas, por el camino que va desde Taninga a Chancaní, a ca. 5 km de Taninga, 15 Mar 2012, *Chiarini et al. 928* (CORD); San Justo, La Francia, RN 19 y calle Cagnolo, 16 Nov 2013, *Chiarini 1033* (CORD); San Javier, Yacanto, 12 Mar 1994, *Cosa 162* (CORD); Cruz del Eje, Serrezuela, Punta de Sierra, 7 Jun 1945, *Cuezzo 898* (BM, K); San Justo, entre Marull y La Para, frente a la Laguna Mar Chiquita, 18 Apr 1987, *Di Fulvio 831* (CORD); Pocho, camino de Taninga a Las Palmas, Feb 2007, *Giorgis 693* (CORD); Salinas Grandes, entre el límite con Catamarca y Totoralejos, unos 5 km antes de Totoralejos (al E de las vías férreas), 26 Apr 1964, *Hunziker 17347* (COL); Cruz del Eje, saliendo de Soto, rumbo al oeste, hacia Paso Viejo, 12 Mar 1990, *Hunziker et al. 25385* (CORD); Próximo a Mar Chiquita, 22 Mar 1988, *Juliani 34* (CORD); Río Primero, Arroyo de La Parra y Los Molles, 25 Feb 1887, *Kurtz 4744* (CORD); Capital, Quebrada de las Rosas, 20 Mar 1956, *Lanfranchi 1315* (SI); Capital, Estancia Germania pr[ope] Cordoba, Jun 1874, *Lorentz 9* (BM, G, W); Cruz del Eje, Cruz del Eje a 10 km de la ciudad, 29 Jan 2001, *Matesevach s.n.* (CORD); Calamuchita, Falda del Sauce (Prop. Marcellino), 19 Apr 1985, *Moscone 111* (CORD); San Justo, Ruta nacional n° 19, inmediaciones de Jean Marie, hacia La Francia, 8 Feb 2001, *Moscone* & *Hunziker 245* (CORD); San Marcos, 23 Jan 1941, *Nicora s.n.* (SI); La Oyada, 2 Feb 1908, *Puyssegur s.n.* (SI); Tulumba, entre Mansilla y km 907, 25 Jan 1947, *Ragonese* & *Piccinini 6390* (BAB, CORD); Punilla, Huerta Grande, 16 Feb 1897, *Stuckert 1720* (CORD); Punilla, La Falda, Jan 1898, *Stuckert 4300* (CORD); Ischilín, Quilino, 20 Feb 1899, *Stuckert 6517* (CORD); Río Cuarto, Río Cuarto, 20 Feb 1900, *Stuckert 8592* (CORD); San Alberto, Mina Clavero, 10 Dec 1901, *Stuckert 10520* (CORD); Río Primero, Estancia San Teodoro, Mar 1916, *Stuckert 23189* (CORD); Río Segundo, Pilar, 18 Feb 1990, *Subils s.n.* (CORD); Marcos Juárez, Río Carcarañá, a la altura de Los Surgentes, 1 May 1985, *Torriglia 327* (CORD); Ischilín, Deán Funes, RN 60, estacion de Servicio YPF, 27 Mar 2012, *Urdampilleta et al. 583* (CORD); San Javier, Cumbre de Achala (Falda O), Las Tapias, al ESE de Villa Dolores, 22 Jan 1990, *Zygadlo 4* (CORD). **Corrientes**: Goya, en los alrededores de la ciudad, 26 Nov 1945, *Boelcke 1412* (SI); Capital, Ciudad, 26 Mar 1944, *Ibarrola 151* (BM); San Roque, San Roque, 2 leguas al sud, 18 Apr 1945, *Ibarrola 2955* (W); Capital, a 2 km del acceso a paraje Perichón, 2 Nov 2010, *Medina et al. 57* (CTES); San Cosme, near Santa Ana, 13 Feb 1961, *Pedersen 5789* (C, CORD, K, NY); San Cosme, near Santa Ana [de los Guácaras], 12 Feb 1961, *Pedersen 5790* (K); Corrientes, Quinta La Eloisa, 6 May 1969, *Plowman 2711* (K); Saladas, San Lorenzo, 18 Feb 1950, *Schwarz 9758* (K). **Entre Ríos**: Uruguay, Colonia Caseros, cerca del Palacio San José, a ca. 5 km al oeste de Concepción del Uruguay, 9 Feb 1991, *Bernardello* & *Galetto 746* (CORD); La Soledad, 1904, *Britton s.n.* (BM); Islas del Ibicuy, Desvío a Médanos, *Burkart 23491* (SI); Paraná, Ruta Nacional 18 entre Paraná y Viale, 16 Nov 2013, *Chiarini 1034* (CORD); Nogoyá, Sobre ruta provincial 26 en la entrada a Nogoyá, 20 Nov 2013, *Chiarini 1039* (CORD); Paraná, Parque Urquiza, 25 Feb 1990, *Cosa 115* (CORD); Diamante, Salto, 26 Nov 1946, *Huidobro 3514* (W); Paraná, Paraná: entrada al campig Toma Vieja, muy cerca del Túnel Subfluvial, 19 Dec 1985, *Hunziker 24860* (CORD); Concepción del Uruguay, Concepcion del Uruguay, Feb 1877, *Lorentz 905* (BM, CORD, GOET, K); Nogoyá, Estancia Las Aguadas, 3 Apr 1967, *Pedersen 8235* (K); Paraná, Paraná, 27 Dec 1928, *Serrano 128* (SI). **Formosa**: Matacos, Ingeniero G.N. Juárez. Barrio Obrero, orillas del pueblo, 27 Feb 1983, *Arenas 2360* (BACP, CORD); Patiño, 5 km antes de Las Lomitas, cerca del aeropuerto, 18 Dec 1984, *Bernardello 503* (CORD); Laishí, RP 5, a un par de km del empalme con la RN 11, 5 Mar 2012, *Chiarini* & *Wahlert 890* (CORD); Patiño, RN 81, entre Ibarreta y Las Lomitas, 5 Mar 2012, *Chiarini* & *Wahlert 892* (CORD); Patiño, RN 81, a ca. 6 km de Pozo del Tigre viniendo desde Las Lomitas, 6 Mar 2012, *Chiarini* & *Wahlert 899* (CORD). **La Pampa**: Utracán, General Acha, 18 Feb 1948, *Burkart 15989* (SI); Utracán, Ataliva Roca, 16 Jan 1990, *Cocucci 439* (CORD); Ruta 154, km 100-101. ca. 40 km al norte de Río Colorado, 17 Jan 1990, *Cocucci 440* (CORD); cruce de ruta prov 26 con RP 19, 31 Oct 2012, *Cocucci* & *Sérsic 5041* (CORD); Caleu-Caleu, desde Hucal, 41 km antes de La Aldea por Ruta Nacional 154 (km 100), 1 Apr 2003, *Di Fulvio 1126* (CORD); Catriló, La Gloria F.C.O, 2 Apr 1944, *Fortuna 22* (BM, K, SI); entre Chacharamardi y La Reforma, 16 km antes de La Reforma, sobre la izquierda de la ruta, 28 Apr 1990, *Galetto 237* (CORD); Jacinto Arauz, 13 Dec 1951, *Solbrig 140* (SI); Lihuel Calel, Sierras de Lihuel Calel, 6 Mar 1976, *Steibel* & *Troiani 4092* (CORD). **La Rioja**: General Belgrano, Las Chacritas, a ca. 35 km al oeste de Jagüe hacia la Laguna Brava, Alrededores del campamento de la Empresa Benito Roggio, 7 Feb 2001, *Barboza et al. 220* (CORD); Vinchina, desde Laguna Brava rumbo Jagüé, en el Campamento de B. Roggio, 16 Dec 2011, *Barboza et al. 3131* (CORD); Chilecito, Cuesta de Miranda, 17 Dec 2011, *Barboza et al. 3159* (CORD); Famatina, alrededores de la Hostería de Famatina, 17 Dec 2011, *Barboza et al. 3160* (CORD); Capital, desde Chumbicha rumbo a Mazán por RN 60, km 148-149, 11 Feb 2012, *Barboza et al. 3449* (BM, CORD); Chamical, Ruta nacional 79, entre Chamical y Olta a 2-3 km de la primera, 7 Mar 1988, *Biurrun* & *Pagliari 2103* (CORD); General San Martín, E of Chepes, c. 4 km W of crossroad RN 79 and RN 141, 13 Jan 2009, *Chiapella* & *Vitek 2175* (CORD, W); Castro Barros, Anillaco, 10 Nov 2011, *Chiarini 781* (CORD); Famatina, Chilecito Cueva de las Brujas, 17 Nov 2015, *Cocucci 5760* (CORD); Independencia, Ruta 74, entre Cueva del Chacho y Patquía, 1 May 1997, *Di Fulvio 949* (CORD); General San Martín, Por Ruta prov. 32, 5 km antes del empalme con Ruta nac. 141 desde Tello a Chepes (36 km antes de Chepes), 12 Feb 2008, *Filippa et al. 79* (CORD); Arauco, Villa Mazán, 20 Dec 1989, *Galetto et al. 232* (CORD); Vinchina, Vinchina, 21 Feb 1879, *Hieronymus* & *Niederlein 288* (CORD, E); General Belgrano, Ruta Nacional 38 (km 217-218), entre el límite con Prov. Córdoba y Castro Barros, 12 Mar 1990, *Hunziker, A.T.et al. 25395* (CORD); Ruta nacional 38 (km 230/232), entre Castro Barros y Chañar, 12 Mar 1990, *Hunziker 25936* (COL); Vinchina, Jagüé, 10 Feb 1949, *Krapovickas* & *Hunziker 5950* (BAB, CORD); Famatina, Las Gredas, 21 Feb 1907, *Kurtz 14406* (CORD); Aracico, Aimogasta, 12 Dec 1948, *Marín 150* (BH, NY, W); Rosario Vera Peñaloza, Ciudad de Chepes, 29 Jan 2001, *Matesevach s.n.* (CORD); General Ortíz de Ocampo, El Milagro, 29 Jan 2001, *Matesevach s.n.* (CORD); Chilecito, 10 Jan 1942, *Meyer 3506* (SI); General Lamadrid, Villa Castelli, 19 Jan 1942, *Meyer 4067* (SI); General Belgrano, por Ruta prov. 79, entre Chañar y Olta a 700 m del primero, en campo Experimental del Instituto Castro Barros, 24 Oct 1996, *Riedel 54* (CORD); La Diana, Apr 1900, *Stuckert 9337* (CORD). **Mendoza**: Las Heras, Uspallata, Ruta Nac. 7, 1 km al W de la villa de Uspallata, 28 Feb 1985, *Ambrosetti* & *Moscone 1480* (CORD, MERL); San Rafael, Salina del Diamante, en los alrededores más cercanos a la propia salina, 26 Nov 2015, *Barboza et al. 4450* (CORD); San Felipe de Aconcagua, Mar 1899, *Bridges s.n.* (W); Luján de Cuyo, El Carrizal, entre Ugarteche y El Carrizal de Arriba, 27 Feb 1985, *Del Vitto* & *Moscone 850* (CORD, MERL); Las Heras, Reserva Pcial. Villa Vicencio, 38, 8 km de Las Heras, camino a Villa Vicencio por ruta 52, 29 Jan 2010, *Ferrucci et al. 2953* (CORD, CTES); Tupungato, Ruta 89, trayecto Tupungato-Potrerillos, 6, 5 km de San José, sentido NW, río Las Carreras, 31 Jan 2010, *Ferrucci et al. 2979* (CORD, CTES); Luján, Lunlunta, 8 Jan 1948, *Garcia 413* (W); Capital, Parque General San Martín, CRICYT, 21 Nov 1991, *Illanes* & *Pacheco 104* (CORD); Las Heras, Quebrada del Camino, above Uspallata on road to Las Cuevas and Chilean frontier, 5 Feb 2013, *Knapp et al. 10470* (BM, CORD, MERL); Las Catitas, 5 Dec 1939, *la Barrera 20* (SI); Junín, Finca San José, Calle Cervalaán, 4 Feb 1945, *Lourteig 1019* (W); General Alvear, Ruta 188, 8 Feb 1987, *Naranjo et al. 937* (SI); Capital, Mendoza (Parque), 19 Jan 1944, *O’Donell 224* (CORD); Las Heras, Laguna La Seca, Ruta Nac. 40, 23 Feb 2008, *Pensiero et al. 7374* (SF); San Rafael, Cuadro Benega, 12 Nov 1949, *Reales 2015* (CORD); Luján, Luján de Cuyo, 4 Mar 1947, *Villafañe 937* (W); Luján, Chacras de Coria, 3 Mar 1947, *Villafañe 903* (CAS); Maipú, 7 Sep 1947, *Villafañe 993* (DS); San Rafael, Valle del Río Atuel, Jan 1897, *Wilczek 239* (CAS). **Neuquén**: Confluencia, Plaza Huincul, 13 Jan 1960, *Articó 244* (CORD); Collón Curá, Bajada colorada: entre Piedra del Águila y Neuquén, 2 Feb 1960, *Articó 250* (CORD); Confluencia, en los alrededores del aeropuerto de Cutral Có, 29 Jan 1990, *Barboza 76* (CORD); Chos Malal, Chos Malal, 20 Jan 1964, *Boelcke et al. 11691* (BAA, BAB, SI); Zapala, San Antonio, a 20 km de Zapala rumbo a Neuquén por RN º22, 14 Feb 2007, *Chiapella et al. 1804* (CORD, CTES, SI); inter Zapala et Cutral Co, prope diversorium ruta 22 cum ruta 34, 11 Dec 1988, *Fernández Casas 11162* (MA); Confluencia, sobre un baldío cerca de la Universidad, 2 May 1990, *Galetto 239* (CORD). **Río Negro**: vicinity of General Roca, Sep 1914, *Fischer 238* (BM, K, NY, SI); General Roca, Allen, Feb 1939, *Hunziker 20* (CORD); Pichi Mahuida, Pichi-Mahuida, 23 Nov 1944, *O’Donell 1712* (W); Avellaneda, Choele-Choel, 27 Nov 1944, *O’Donell 1815* (W); General Roca, Cipolleti, 4 Dec 1944, *O’Donell 1910* (W); Pichi Mahuida, Río Colorado, barrancas del Río, en cruce ruta 3, 18 Feb 2008, *Pensiero et al. 7209* (SF). **Salta**: Cachi, San José de Cachi, 28 Mar 1979, *Cabrera et al. 30771* (SI); General Güemes, por Ruta 34, entre General Güemes y Metán, 5 km antes del Río Juramento, 17 Dec 1989, *Galetto et al. 214* (CORD); Rosario de la Frontera, Rosario de la Frontera, 19 Feb 1947, *de la Sota 132* (W); Capital, Cerro San Bernardo, 4 Feb 1949, *Legname 221* (CORD, LIL); Anta, El Dorado, 5 May 1948, *Luna 1021* (DS); Coronel Moldes, 2 Jan 1945, *Mintzer s.n.* (SI); Coronel Moldes, 2 Jan 1945, *Mintzer s.n.* (SI); Candelaria, El Datil, 7 Feb 1949, *Montenegro 380* (W); Capital, Ciudad de Salta, vías del FFCC, playa de maniobras entre la estación y molino Batule, 5 Dec 1988, *Novara 8248* (CORD); La Viña, Coronel Moldes, entrada da cidade, 25 Jan 2007, *Paula-Souza et al. 7856* (ESA, K, SI); Rivadavia, Ruta 81, km 1517, 16 Nov 2009, *Schinini* & *Flaschland 37059* (CTES); Rosario de la Frontera, Pasando Rosario de la Frontera, 13 Nov 1984, *Subils 3589b* (CORD); Guachipas, Alemania, 16 Nov 1929, *Venturi 9882* (BM, NY); Chicoana, Ruta Prov. 33, de Pulares a San Fernando de Escoipe, 15 Feb 2007, *Zuloaga et al. 9373* (SI). **San Juan**: Calingasta, Barreal, cerca de la Gendarmería, 1 Nov 1969, *Ariza Espinar 2393* (CORD); Caucete, Rumbo a la Difunta Correa, desvío de 2 km hacia la derecha, en el km 195 de la RN 141, 21 Dec 2007, *Barboza 1944* (CORD); Sarmiento, Media Agua, 28 Jan 1950, *Borsini 1333* (CORD, LIL); Ullum, Hualilán, 8 Dec 1979, *Cabrera et al. 31065* (SI); Ullum, road RN 40 between San Juan city and Talacasto, 14 Jan 2009, *Chiapella* & *Vitek 2186* (CORD, NY, W); Ullum, entre ciudad de San Juan y Ullún, cerca de la empresa Valle de la Luna S.A, 4 Apr 1998, *Cocucci et al. 11* (CORD); Trinidad, 20 Nov 1945, *Cuezzo 1302* (BM, K); Jáchal, Entre El Balde y Tucunuca, unos 7 km después de Ing. Matías G. Sanchez (ruta 40), 10 Feb 2002, *Di Fulvio 1112* (CORD); Iglesia, R 149, alrededores de Iglesia, 7 Mar 2011, *Fortunato et al. 9942* (BAB, CORD); Sarmiento, Puesto Bachongo, 25 Jan 1986, *Guaglianone et al. 1555* (SI); Rawson, Dique Bello, 26 Jan 1986, *Guaglianone et al. 1571* (SI); Caucete, Marayes, 8 Nov 1986, *Haene 61* (SI); Valle Fértil, Puesto de las Chaves, cerca de Mareyes Viejo, 16 Feb 1921, *Hosseus 2638* (CORD); Caucete, Ruta 20, km 1158, entre Caucete y Vallecito (=Difunta Correa), 13 Jan 1979, *Hunziker et al. 23318* (CORD); Jáchal, Camino a Mogna, 9 Nov 1980, *Kiesling 3006* (K, SI); Ullum, Talacasto a Alto de Colorado, 17 Nov 1982, *Kiesling* & *Sáenz 4241* (SI); Angaco, Sierra de Pie de Palo, ruta cerca de Angaco, 23 Nov 1984, *Kiesling et al. 4827* (SI); Calingasta, 5 km N of Villa Nueva (measured from the bridge over the Río de Castaño Viejo), on Ruta 412 towards Iglesia, 11 Jan 1995, *Leuenberger et al. 4459* (B, CORD, Z); Chimbas, Finca de la familia Matesevach, calle Oro 4730, 1 Jan 2001, *Matesevach s.n.* (CORD); Iglesia, Reserva de San Guillermo, Baños Termales de San Crispín, 10 Jan 1984, *Meglioli 33* (SI); km 24, camino de Mendoza a San Juan, 31 Jan 1946, *Nicora 4282* (SI); Jáchal, Entre Jáchal Estación Adan Quiroga, 19 Jan 1950, *Palacios et al. 1695* (CORD, LIL); Reserva de San Guillermo. Baños de San Crispín camino de deyección sobre las vegas termales, 24 Mar 1983, *Pujalte 210* (SI); Iglesia, curso superior del Río Valle del Cura, Llanos de Conconta, Agua de Alutiaca, 25 Jan 1981, *Subils 2909* (CORD); Capital, Estación Ferrocarril, 22 Jan 1972, *Volponi 241* (SI). **San Luis**: Ayacucho, a orillas del Río Luján, 19 Dec 2007, *Barboza et al. 1923* (CORD); Capital, Chosmes, 25 Jan 1950, *Borsini 1013* (W); Chacabuco, Concarán, alrededores del Establecimiento El Sauce, 4 Feb 2012, *Chiarini et al. 824* (CORD); Junín, Ruta nacional 146: entre Quines y Villa Dolores, cerca del cruce hacia La Unión, Jan 1988, *Cocucci 220* (CORD); La Capital, entre el Chorrillo y Cruz de Piedra, por la ruta 20, 2 Dec 1988, *Del Vitto* & *Petenatti 3558* (CORD); Ayacucho, Ruta 20, km 390, La Chañarienta, 9 Feb 2002, *Di Fulvio 1110* (CORD); Chacabuco, Estanzuela, 4 Mar 1882, *Galander s.n.* (CORD); La Capital, a la salida de San Luis, sobre la rotonda que separa las rutas que van a Potrero de Funes y Trapiche, 14 Nov 1988, *Galetto 24* (CORD); Buena Esperanza, 24 Nov 1962, *Hunziker 16035* (COL); Las Pampas cerca de San Luis, 6 Mar 1863, *Isern 8096* (SI); Junín, Merlo, frente al aeródromo, 10 Apr 1988, *Juliani 37* (CORD); San Luis del Palmar, in Bahnhofsgelande von Mercedes, ca. 90 km ESE San Luis, 9 Feb 1993, *Starlinger 22 -93* (W); Desaguadero (límite entre San Luis y Mendoza), 26 Jan 1939, *Troncoso 6030* (SI); Ayacucho, Luján, 2 Dec 1944, *Varela 713* (BM, K). **Santa Fé**: San Justo, Soledad, 3 Dec 1946, *Álvarez 989* (W); General López, Southern District, Venado Tuerto, Dec 1975, *Ardiar s.n.* (K); General Obligado, Cerca de Berna, Ruta 11, km 759, 25 Jan 1992, *Bernardello* & *Galetto 788* (CORD); 9 de Julio, RN 95, 8 Mar 2012, *Chiarini* & *Wahlert 917* (CORD); San Cristóbal, Saliendo de La Rubia, 17 Dec 1981, *D´Angelo 285* (SF); San Cristóbal, entre San Cristóbal y Gobernador Crespo, Ruta 39, 4 Jan 1985, *Di Fulvio 799* (CORD); Cuty Lai, 6 Apr 1917, *Hosseus 65* (CORD); San Lorenzo, Carcarañá, *Kurtz 5117* (CORD); Castellanos, Cañada Las Calaveras. Proximo a Lehmann, 24 Apr 1989, *Luchetti 5* (SF); San Cristóbal, Ceres, 17 Jan 1989, *Luchetti et al. 6* (SF); Las Colonias, Reserva UNL, 21 Nov 1991, *Marino 201* (SF); Vera, 10 km al N de Fortín Olmos, campo de Sr. Sanchez, 11 Nov 2003, *Marino 1914* (SF); Vera, Campo del Sr. Sanchez, 10 km al norte de Fortin Olmos, 11 Nov 2003, *Marino 1942* (SF); Vera, Escuela EFA, km 50, Ruta 98 S, 13 Nov 2003, *Marino 2047* (SF); San Justo, 5 km al O de Naré, Cañada Naré, 28 Dec 1985, *Pensiero* & *Faurie 2424* (SF); Las Colonias, Esperanza, Reserva de la Escuela Granja, 25 Jan 2000, *Pensiero* & *Exner 6063* (SF). **Santiago del Estero**: Santa Rosa, 5 Jan 1938, *Argañaras 51* (SI); entre Brea Pozo y Río Huayco Hondo, 23 Mar 1943, *Bartlett 19760* (SI); Río Hondo, Termas de Río Hondo. Ruta 9, 26 Jun 1991, *Cosa 124* (CORD); General Taboada, Averias, 23 Jan 1985, *Fernandez 156* (CORD); Fortin Tostado, Jun 1906, *Goubat s.n.* (SI); General Taboada, Tacanitas km 443, 17 Apr 1917, *Hosseus 233* (CORD); Atamisqui, Isla Verde, Por ruta 9, Isla Verde, entre El Guanaco y San Gregorio, 11 Feb 1986, *Hunziker et al. 24868* (BM); Ojo de Agua, Entre el límite con Córdoba y Ojo de Agua, unos 10 km al sur de Ojo de Agua, 13 Sep 1987, *Hunziker 25089* (CORD); Copo, Ruta 16, 15 km NW de Los Tigres, 28 Jan 2007, *Paula-Souza et al. 8046* (ESA, SI); Robles, Colonia Jaime, 9 Nov 1948, *Ruiz 53398* (BA, CORD); Moreno, Quimili, 24 Feb 1947, *Schulz 1363* (W); Añatuya, 27 Jan 1944, *Soriano 548* (BAB, SI); Choya, Ruta Nacional 158: alrededores de Frías, camino a San Antonio, 1 Jun 1990, *Subils* & *Guzmán 4414* (CORD); Quebracho, Sumampa, 6 Jan 1948, *Terribile 808* (W). **Tucumán**: alrededores de Tucumán, 8 Dec 1907, *Dinelli 654* (BA, SI, SI); Graneros, La Madrid, 26 Jan 1886, *Kurtz 4222* (CORD); Graneros, La Madrid, 26 Jan 1886, *Kurtz 4224* (CORD); Trancas, Tapia, 28 Nov 1920, *Venturi 1091* (SI); Burruyacú, El Puestito, 2 Dec 1928, *Venturi 7678* (BM, CAS, K, SI).

**Bahamas**. **Eleuthera**: Hatchet Bay Farm, 24 Jul 1969, *Proctor 30960* (BM); edge of cultivated field 2 miles south of Gregory Town, 2 Jun 1979, *Sauleda 2649* (MO, NY).

**British Virgin Islands**. **Anegada**: around The Settlement, 1 Aug 1970, *D’Arcy 4861* (MO); **Tortola**: East End, vacant lot, 15 Apr 1965, *D’Arcy 44A* (BM).

**Chile**. **Región II (Antofagasta)**: El Loa, Patio del Hostal Puri, San Pedro de Atacama, 16 Feb 2001, *Ackermann 218* (BM); El Loa, Río Grande, San Pedro de Atacama, 1 Dec 2008, *Baines et al. 255* (BM, E); El Loa, Calama, beside Ensueno land development area, part of the Stadium in Calama, 12 Mar 1988, *Dunnett 13* (K); Hostería San Pedro de Atacama, 5 Feb 1967, *Martin 272* (SI). **Región III (Atacama)**: Caldera, prov. Atacama, 21 Feb 1939, *Beetle 26132* (BH, K); Paipote, 25 Jan 1989, *Cocucci 369* (CORD); Copiapó, 30 km S of Copiapo on road to Los Loros, 17 Sep 1987, *Hannington 23* (K); Huasco, Yerba Buena Chica, 25 miles east of Carrizal Bajo, Vallenar, 25 Dec 1871, *King s.n.* (E); Copiapó, Dorf Tierra Amarilla am Weg zur Mine Las Bateas, 12 Nov 1987, *Rechinger* & *Rechinger 63645* (W); Vallenar, Mine Algarrobo, Nov 1925, *Werdermann 142* (BM, CAS, E, SI); Huasco, all along road from Vallenar to San Felix, 24 Oct 1938, *Worth* & *Morrison 16215* (K). **Región IV (Coquimbo)**: Elqui, Vicuña, 6 Oct 1927, *Elliot 62* (E, K); Elqui, Paiguano, 7 Dec 1997, *Gardner* & *Matthews 58* (E); Vicuña, a 62 km al este de La Serena, en las inmediaciones de la bodega pisquera Capel, 22 Feb 1996, *Hunziker 25554* (CORD); Elqui, NE of Monte Grande, Paiguano, 2 Mar 1996, *Instituto de Investigaciónes Ecológicas Chiloé 502* (E). **Región IX (Araucanía)**: El Loa, San Pedro de Atacama, 28 Feb 1988, *Wickens* & *Dunnett 828* (K). **Región V (Valparaíso)**: San Felipe de Aconcagua, 1868, *King s.n.* (E); Los Andes, 18 Jan 1904, *Scott-Elliot 442* (BM, E). **Región VIII (Bío-Bío)**: Ñuble, Chillán, *Gillies 669* (K).

**Colombia**. **Cundinamarca**: prés Bogotá, Chaquiero, Jul 1908, *Apollinaire s.n.* (E) [possibly cultivated?].

**Cuba**. **Cienfuegos**: Cienfuegos, 5 Jun 1923, *Ekman 13942* (NY). **La Habana**: Carmelo Hill, Vedado, 13 Jun 1919, *Fr. León 8782* (NY).

**Ecuador**. “Andes”, *Jameson s.n.* (E) [possibly Peru, from Lima?].

**Haiti**. **Sud**: Massif de la Hotte, aprox. 4 km al E de Les Anglais en el camino costero a Port-a-Piment y Les Cayes, 25 Jan 1985, *Zanoni et al. 33168* (JBSD).

**Jamaica**. **Saint Catherine**: Boldes Research Station, ca. 2 miles W of Old Harbour, beside the piggery, 5 Aug 1987, *Collins s.n.* (MO, NY).

**Mexico**. **Aguascalientes**: 35 miles N of Aguascalientes, 24 Aug 1953, *Manning* & *Manning 531244* (MEXU); Rincón de Romos, Colonia 16 de Septiembre, 17 Sep 1989, *Marmolejo P. s.n.* (MEXU). **Baja California**: Mexicali, 26 May 1933, *Harvey 585* (US); La Hechicera, 2 km S of La Hechicera on highway, Sierra Juarez, 7 Jul 1979, *Moran 27703* (CAS); Mexicali, Mexicali, about 14 miles W along road to Tecate, 30 Jun 1962, *Wiggins* & *Thomas 444* (DS, MEXU). **Baja California Sur**: La Paz, Carretera entronque al aeropuerto, km 3 carretera a Ciudad Constitución, 15 Jun 1994, *Domínguez C. 1237* (MEXU). **Chihuahua**: Villa Ahumada, ca. 10 miles S of Villa Ahumada along Highway 45 between Ciudad Juárez and Chihuahua, 1 Aug 1975, *Davidse* & *Davidse 9252* (MEXU); 9.4 miles W of Hwy 45 along Hwy 10, ca. 15 miles SW of Ciudad Juárez, 18 Aug 1971, *Henrickson 5708* (MEXU); Parral, 10 km al este de Parral, 31 Jul 1982, *Hernández Magaña 8352* (CAS, MEXU); Ciudad Juárez, 35 km al S de Ciudad Juárez, 5 Aug 1982, *Hernández Magaña et al. 8415* (MEXU); Juárez, Samalayuca, south of Juárez, 7 Jul 1957, *Knobloch 70* (BM); along Hwy 45, km 1615-1616, S of Chihuahua, 20 Jun 1965, *Mertz 15* (MEXU); Aldama, km 68 carretera Chihuahua-Ojinaga, 25 Aug 1978, *Molinar et al. 47* (MEXU); Ahumada, Laguna Santa María, near Lake Santa Maria, 7 Sep 1899, *Nelson 6429* (K); Chihuahua, plains and mesas near Chihuahua, 16 Aug 1887, *Pringle 1566* (BM, K, MEXU); between Cuidad Juárez and Cuidad Chihuahua on Hwy 45, 33 miles from US border, 7 Aug 1984, *Randolph 173* (MEXU); entronque a Santa Clara, km 91 Chihuahua-Ciudad Juárez, 12 Jun 1980, *Siqueiros* & *Baray 515* (MEXU); Saucillo, Naica, 14 Sep 2011, *Tejero Diez* & *Canek 6517* (MEXU); Villa Ahumada, 15 km al N de Villa Ahumada, km 102 carretera Chihuahua-Ciudad Juárez, 13 Sep 1978, *Valdés et al. 15 -1978* (MEXU); Jiménez, Escalón, 26 Jul 1939, *White 2052* (CAS); Janos, border of Chihuahua and Sonora, 26 Aug 1939, *White 2526* (MEXU). **Coahuila**: Saltillo, 12 miles E on Mexican Highway 40, 4 Apr 1964, *Canedo et al. 9075* (DUKE); Torreón, Valle Verde, 21 Sep 1996, *Cano R.* & *López C. 21* (MEXU); along road to E of Hwy 57, 18 mile SE of Saltillo, 15 Jul 1979, *Dunn et al. 23177* (MEXU); General Cepeda, ca.de 0.5 km del entroque a Hipolito, 24 Mar 2009, *Guzman 3013* (K); ca. 444 (air) miles W of Cuatro Cienagas, 3 miles NE of Est. Zacatosa in Bolson de Mapimi region, 21 Sep 1972, *Henrickson 7917* (MEXU); Parras, 0.5 km al sur de San Rafael de los Milagros, poblado que esta a mitad de camino de la cettera Saltillo-Torreón, 16 Oct 2000, *Montero Castro* & *Torres C. 136* (MEXU); San Pedro, along hwy. Mex. 30, 108 km by road NE of San Pedro de Las Colonias, 27 Jul 1982, *Nee* & *Diggs 25319* (CORD, F, XAL); Ramos Arizpe, Paredón, 1 Oct 1924, *Orcutt 1270* (DS); San Lorenzo de Laguna, Feb 1880, *Palmer 935* (K); Saltillo, Fraile, 59 km S of Saltillo, 10 Jul 1941, *Stanford et al. 273* (DS, MEXU); 18 m W of Concepción de Oro, on mountain, 24 Jul 1941, *Stanford et al. 586* (DS, NY); Torreón, Ejido El Tajito, 10 km al E de Torreón, 21 May 1965, *Vázquez s.n.* (MEXU); along Mex. 40 30 miles W of Monterrey, ca. 25 miles NW of Saltillo, 29 Jun 1966, *Ward 5742* (DUKE); Monclova, 5 Jul 1939, *White 1712* (DS); Ramos Arizpe, between Hipolito and Sacramento, in El Desierto de Payla, 15 Jun 1936, *Wynd* & *Mueller 76* (K, NY); Torreón, near Horizonte, 24 Aug 1937, *Wynd 780* (K, NY). **Colima**: Tecomán, 2 km de Calera rumbo a Madrid, 28 May 1990, *Román Miranda 1337* (MEXU). **Distrito Federal**: City of Mexico, Nov 1884, *Carruthers s.n.* (BM); Ticomán Cuauhtepec, Jul 1952, *Gallegos Harking 81* (MEXU); Rosario, 1 Sep 1936, *MacDaniels 703* (BH); Azcapozalco, 1 Apr 1950, *Matuda 19545* (MEXU); Xacateno, 1 km al N de la Unidad Profesional de Zacateno, 17 Aug 1969, *Pérez H. 86* (DS); E. de Agricultura, Valle de México, 14 Apr 1882, *Urbina s.n.* (MEXU). **Durango**: Ceballos, 1 Aug 1979, *Aguilar C. et al. 164* (MEXU); Durango, La Ferreria, Río El Tunal, Jul 1992, *Aguirre T. 45* (MEXU); Durango, P.P. Morelos, Oct 1994, *Aguirre T. 151* (MEXU); Mapimí, 45 km al E de la Ciudad de Ceballos [26.29-52 DMS N/103.32-58 DMS W], 26 Jun 1981, *Alba 45* (MEXU); Santiago Papasquiaro, km 3 de la carretera Santiago Papasquiaro-Los Altares, 30 Jul 1990, *Benítez P. 1814* (MEXU); Durango, km 27 carretera Durango-Torreón, Jul 1994, *Domingo* & *Aceval A. 524* (MEXU); Durango, 7 km al NE de Durango, 7 Jul 1982, *Hernández Magaña* & *Tenorio L. 7714* (MEXU); Mezquital, km 20 carretera Durango-Mezquital, Sep 1992, *Martínez Marín 416* (MEXU); Durango, camino a Malaga, km 1, Jun 1993, *Martínez Marín 464* (MEXU); Mapimí, 49 km al NE de Ceballos, Rancho San Ignacio (Ojo de Agua), 25 Oct 1975, *Martínez O. et al. 413* (K); Poanas, Rancho 4 Hermanos, Jun 2000, *Martínez 683* (MEXU); Durango, city of Durango and vicinity, Apr 1896, *Palmer 233* (BM, E, G, MEXU, NY); Pénuco de Coronado, km 38 carretera Durango-Torreón, May 1994, *Rangel Ramírez 130* (MEXU); San Juan de Guadelupe, Ejido Acacio, 12 Jul 1990, *Rendón s.n.* (MEXU); Mezquital, Durango, 50 miles S of Durango, 10 Aug 1953, *Selander 4-53* (DS); Cuencamé, Velardeña, 23 km al NNE del centro Cuencamé, 22 Jul 2010, *Tejero-Díaz* & *Torres 6256* (MEXU); Tepehuanes, Presidios, 12 km al S de Tepehuanes, 19 Jul 1982, *Tenorio L.* & *Romero de T. 1122* (MEXU); Mapimí, Puente de Hojuelas, 8 km SE de Mpio de Mapimí, 8 Sep 1983, *Torrecillas 208* (MEXU); Conejos, 25 miles S of Ceballos, ca. 50 miles N of Gomez Palacio, 3 Jul 1966, *Ward 5771* (DUKE). **Guanajuato**: carretera Irapuato a Mungía, 20 May 1973, *Boege 2793* (MEXU); Apaseo el Grande, Apaseo, 1938, *Figueroa s.n.* (MEXU); San Felipe, 10 km al SW de San Felipe, 25 Aug 1990, *Galván* & *Galván 3614* (MEXU); San Miguel de Allende, highway 51, 21 Jun 1971, *Genelle* & *Fleming 819* (DUKE, MEXU); Guanajuato City to San Luis de la Paz, 14 Sep 1946, *Hernández X. et al. X-2362* (MEXU); San Miguel Allende, el sendero que continua al S de Calle Recreo, 19 Sep 1977, *Kishler, J. 145* (MEXU); Irapuato, 1.66 km al OSO de Lo de Juárez, 7 Sep 2007, *Martínez S.* & *Ramos 39643* (MEXU); Predio El Cortijo, a 16 km al NE de la ciudad Dolores Hidalgo sobre la carretera a San Luis de la Paz, 4 May 1996, *Ocampo 39* (MEXU); León, Lagunillas, 28 Jun 1996, *Pérez C.* & *Zamudio 3359* (MEXU); Irapuato, 10 May 1901, *Pringle 9370* (BH, K); Dolores Hidalgo, Las Yerbas, 17 May 1996, *Rojas Villegas s.n.* (MEXU); Estación Río Laja, 19 Jul 1973, *Valdés et al. 55- 2* (MEXU); San Luis de la Paz, Rancho La Misión, 8 km al NE de San Luis de la Paz, 11 Oct 1988, *Ventura* & *López 6112* (MEXU); San Luis de la Paz, Potosino Dos, 24 May 1990, *Ventura* & *López 8022* (MEXU); San Luis de la Paz, Cerro Prieto, 19 Jul 1990, *Ventura* & *López 8329* (MEXU). **Hidalgo**: Actopan, El Arenal, Mar 1936, *Bravo H. s.n.* (MEXU); Zimapán, Zimapán, *Coulter 1246* (K); Ajacuba, Cerro El Crestón, ca. 2 km antes de llegar a la desviación al centro de Emiliano Zapata, rumbo a Ajacaba, Ejido Tecomatlán (de E a W), 24 Aug 1988, *Díaz Vilchis 124* (MEXU); Ajacuba, Rincón del Gato, barranca al N del poblado Emiliano Zapata, Sierra de Chicavasco, ejido Emiliano Zapata, 28 May 1989, *Díaz Vilchis 438* (MEXU); Tecozautla, 4 km al oeste en el desvío a Pañhé, 11 Jul 1980, *Hernández Magaña 4642* (CAS, MEXU); Pachuca, San Bartolo, 2 km al W de Pachuca, 22 Jun 1977, *Medina C. 2036* (MEXU); El Arenal, valley of Actopan between El Arenal and Jiadi, 26 Oct 1946, *Moore 1640* (BH); Cerro Gordo, 5 km al W de Pachuca, 18 Aug 1972, *Rzedowski 29179* (CAS, NY); Santiago de Anaya, 500 m al O de Santiago de Anaya, 5 Jun 1992, *Soriano M. 144* (MEXU); Tepeapulco, 2 Jun 1976, *Ventura A. 1509* (MEXU). **Jalisco**: 21.1–24.1 km de la salida de San Juan de los Lagos por la carretera a Lagos de Moreno, 28 Jul 1978, *Guzmán et al. 999* (MEXU); Ojuelos, 3 km N, 17 Sep 1946, *Hernández X. et al. X- 2493* (MEXU); Villa Hidalgo, Villa Hidalgo, May 1977, *la Torre 47* (MEXU); Amacueca, cerca de Cofradía del Rosario, 1 Aug 1993, *Novoa L.* & *Villa M. 919* (MEXU); 5 km antes de llegar a Lagos de Moreno en la desv. hacia la Mesa, 31 Jul 1987, *Román Miranda, C-742* (MEXU); Ojuelos, 3 km al E de Ojuelos, 25 Sep 1962, *Rzedowski 16116* (MEXU). **México**: Cerro de San Cristobal, 2 Jul 1950, *Matuda 18953* (MEXU); Icatepec, [Ecatepec] a Peñon, 28 Jun 1953, *Matuda 28754* (MEXU); Huehuetoca, Tajo de Nochistongo, 22 Jun 1981, *Romero* & *Rojas 1405* (MEXU); Huehuetoca, Huehuetoca, vía de ferrocarril, 22 Jun 1981, *Romero* & *Rojas 1406* (MEXU); Venta de Carpio, 16 Jul 1930, *St Pierre 2601* (K); Otumba, Ahuatepec, 27 Jun 1976, *Ventura A. 1361* (MEXU); Otumba, Ahuatepec, 15 Jul 1976, *Ventura A. 1595* (CORD, ENC, MEXU). **Michoacán**: Pátzcuaro, a 300 m de la salida a Uruapan, sobre la vía del FF.CC, 29 May 1986, *Escobedo 928* (MEXU); Morelia, La Huerta, Estación del Tren, 22 Aug 1988, *Escobedo 1594* (MEXU); Coahuayana, Boca de Apiza, 16 Jul 1985, *Soto N. 9508* (MEXU). **Morelos**: Zacatepec, [Zacatepec de Hidalgo], 5 Apr 1969, *Vázquez 2164* (MEXU). **Nuevo León**: Monterrey, ½ mile West of ITESM Campus, 29 Jul 1970, *Burge 25* (EIU); 7.8 km E of turnoff to Los Herreras on Mex 40 near Presa of San Juan River drainage, 18 Sep 2001, *Bye et al. 28334* (MEXU); Dr. Arroyo, Tanquecillos, Aug 1842, *Karwinski 583* (LE); near Montemorelos, 19 Apr 1947, *Kelley 21* (BM, MEXU, NY); Los Ramones, 2 kilo. west of Ramones, 70 kilo. east of Monterrey, 10 Jul 1971, *Parker 379* (EIU); La Escondida, N of Monterrey, 1946, *Roybal 904* (MEXU); Montemorelos, Ojo de Agua, 24 Apr 1988, *Tirado 51* (MEXU); Montemorelos, 4.1 miles S on Highway 85, 4 Jul 1969, *Weaver 2044* (DUKE, MEXU); Monterrey, Cerro de la Silla, foot of La Silla, 4 Sep 1937, *White* & *Chatters 183* (MEXU). **Oaxaca**: Salina Cruz, Salina Cruz, dry dock area, 18 Sep 1978, *D’Arcy 12027* (MEXU); Salina Cruz, La Ventosa, al S de Salina Cruz, 31 Aug 1988, *Martínez 1796* (MEXU); Playa Abierta at Salina Cruz about 15 miles S of Tehuantepec, 1 Aug 1984, *Wilbur 36096* (BM). **Puebla**: Zinacatepec, 1 km from Zinacatepec, on the federal Tehuacán-Teotitlan de Flores Magón-Oaxaca road, 17 Jul 2004, *Calzada 24391* (K); Coxcatlán, San Rafael, campos de cultivo al oriente de San Rafael, 24 May 2009, *Cervantes M. 58* (K); Chietla, carretera Axochiapan-Izucár de Matamoros, entre Ahuehuetzingo y Atencingo, 19 Jun 1998, *Guízar N.* & *Herrera 4031* (MEXU); Matamoros, 22 Mar 1941, *Miranda 1418* (MEXU); San José Miahuatlán, Axusco, 4-6 km al S, 18 Oct 1992, *Salinas T. 7104* (CAS). **Querétaro**: cerca acueducto, 4 May 1975, *Argüelles 23* (MEXU); calle cerca de Constituyentes, 31 May 1986, *Argüelles 2468* (MEXU); Ciudad de Querétaro, Barrio Carretas, 19 Jun 2016, *Chiarini 1270* (CORD); Peñon de Bernal, 22 Jul 1984, *Espejo 1023* (MEXU); Cadereyta, Jardín Botánico Regional de Cadereyta Ing. Manuel González de Cosío, 8 Apr 1992, *Hernández Magaña et al. 9752* (MEXU); El Marqués, La Cañada, 12 Jun 2007, *Martínez 6827* (MEXU); Querétaro cerca de las paredes, 20 Apr 1886, *Urbino s.n.* (MEXU); Peñamiller, ladera norte y noreste de Cerro Picacho, 29 Jul 1977, *Zamudio 2305* (MEXU). **San Luis Potosí**: 4 miles NE of San Luis Potosi, 29 Aug 1947, *Barkley et al. 783* (MEXU); Villa de Arriaga, a 22 km de Villa de Arriaga sobre la autopista, San Luis Potosi a Villa de Arriaga, a Lagos de Moreno por el km 54-55, 21 Aug 2009, *Calzada 25305* (K); km 124 carretera Querétaro-San Luis Potosí, 24 Sep 1979, *Equipo 5* (MEXU); km 30 carretera San Luis Potosí-Zacatecas, 7 Oct 1972, *García M. et al. 322* (MEXU); Matehuala, Matehuala, frente del Motel de las Palmas, orilla de la carretera que va hacia Saltillo, entre el motel y la carretera, 24 Sep 1978, *García P. 670* (CAS, MEXU); San Luis Potosí, Colonia Del Valle, 14 Jun 1973, *Gómez Lorence 3* (MEXU); Vanegas, 35.8km de Vanegas a El Salado, 8 Nov 2008, *Guzman 2947* (K); Charcas, Charcas, Jul 1934, *Lundell 5145* (DS, K, MEXU); San Luis Potosí, carretera a la presa de San Jose, 20 Oct 1973, *Moncada 4741* (MEXU); Central Mexico, chiefly in the region of San Luis Potosi, 1878, *Parry* & *Palmer 636* (BM, E); San Luis Potosí, Aug 1876, *Schaffner 695* (K, MEXU); 2 km del camino Rioverde-Tablas, 7 Aug 1960, *Takaki 326* (MEXU); Guadalcázar, 1 km N del crucero Guadalcázar sobre la autopista a Matehuala, 27 Jun 2000, *Torres C. 15684* (MEXU); Guadalcázar, Charco Blanco, 1 km del crucero San Luis Potosí-Matehuala, hacia Guadalcázar (bajos y lomerios entrando por un arroyo), 22 Sep 2012, *Torres C. et al. 17481* (MEXU); Charcas, Jul 1934, *Whiting905* (MEXU). **Sonora**: Plutarco Elios Calles, Sonoyta, NW side of town ca. 0.5 km S of river, 12 Oct 1986, *Felger* & *Joseph 86-400* (MEXU); Puerto Peñasco, Puerto Peñasco, along railroad tracks, middle of town, 25 Jun 1985, *Felger 85-795* (MEXU); Empalme, end of causeway at Empalme, along Mexican Hwy 15, 14 May 1973, *Hansen et al. 1392* (MEXU); Bocum, Ejido Javier Mina, Block 409, Lote 2, 9 Apr 2009, *López Valencia s.n.* (MEXU); Naco, 10 km al SE de Naco, 24 May 1987, *Tenorio L.* & *Romero de T. 13676* (MEXU); Fronteras, Bacoachic, 23.5 miles NE on road to Esqueda, 10 May 1948, *Wiggins 11750* (DS). **Tamaulipas**: Matamoros, Mezquital, 61 km al E del carretera a Matamoros, 3 Oct 1994, *Baro et al. 495* (MEXU); Burgos, El Mulato, vicinity, 16 Aug 1930, *Bartlett 10991* (DS); east of Interamerican Hwy 85 23.7 miles S of Nuevo Laredo, 1 Jul 1969, *Broome 313* (DUKE); Nuevo León, Nuevo Laredo, 14 km (9 miles) S of Nuevo Laredo on road to Monterrey, 17 Apr 1939, *Frye* & *Frye 2383* (DS); Salinas Victoria, Transito Pesados Naciones Unidas, 1 Jun 1995, *Galván 618* (MEXU); Nuevo Laredo, 50 miles southeast of Nuevo Laredo, 28 Mar 1964, *García* & *García 40* (CAS, DUKE); Rancho el Mezquite, 35 km al SE de Santa Teresa, Mar 1963, *González-Medrano 115* (MEXU); Jaumave, Ejido San Antonio, 17 Nov 1985, *Jiménez 426* (MEXU); Nuevo Laredo, 20 km al oeste de [Nueva] Ciudad Guerrero, Oct 1973, *López F.* & *Hernández Magaña 6335* (MEXU); Soto de la Marina, Poblado La Pesca, 24 Jul 2008, *Martínez S.* & *Ibarra 40416* (MEXU); Miquihauana, 8 Aug 1941, *Stanford et al. 802* (DS); Soto la Marina, due E of Cuidad Victoria about 100 miles along hwy 180 that parallels the coast, across from gas station in town, 8 Jul 1983, *Taylor 1898* (DUKE); 30 miles S of Matamoros towards San Fernando, 14 Jul 1984, *Wilbur 35155* (DUKE). **Veracruz**: Tampico Alto, Río Panuco, side road across from Tampico, ca. 2 mi. E of Pueblo Viejo, 11 Jun 1973, *Hansen et al. 1791* (BH, MEXU); Villa Cuauhtemoc, Pueblo Viejo, at ferry landing across from Tampico, 11 Jul 1980, *Nee* & *Hansen 18972* (MEXU); Puerto Viejo, vicinity of Pueblo Viejo, 2 km south of Tampico, 10 Feb 1910, *Palmer 388* (BM, K). **Zacatecas**: Villa de Cos, El Capirote, 3 Jul 1997, *Arteaga Saucedo 840* (MEXU); Pánuco, Valle Hermoso, 21 Jul 1986, *Brigada Zacatecas 86* (MEXU); Calera, Calera, junction of Hwys 45 & 49, 16 Aug 1975, *Gillett* & *Delgado 17013* (MEXU); Río Grande, Rancho El Carrizal, potrero Las Remudas, 7 km al E carretera Zacatecas-Torreó, entrando por km 41 desde el entroque Torreón-Durango, 31 Aug 1979, *González E.* & *Cano s.n.* (CAS); Campo experimental Noris de Guadelupe CNIZA, 35 km S de Concepción de Oro, 12 Jul 1975, *González Medrano et al. 8003* (MEXU); 50 miles from Aguas Calientes on road to Zacatecas, ca. km 695, 7 km S of Ojo Caliente, 9 Sep 1958, *Hawkes et al. 1463* (K); 70 (air) miles NE of Zacatecas, 66 (rd) miles NE of Hwy 41 along Hwy 54, 13 Sep 1971, *Henrickson 6673 a* (MEXU); Francisco Villa, 1 km S (on map Estación Symon), 28 Mar 1973, *Johnston et al. 10443* (CAS, MEXU); Guadelupe, Lo de Vega, 13 Oct 1987, *Medina 241* (MEXU); Fresnillo, km 97–98 carretera Mex. 45 entre Fresnillo y Sombrerete, justo en el crucero a Río La Medina, 5 Sep 2000, *Rodríguez C. et al. 6100* (MEXU); Teul de González Ortega, La Haciendita, 16 Sep 1985, *Saucedo Q.* & *Aceves s.n.* (MEXU); Ojocaliente, Predio La Vibora, 28 Sep 1987, *Silva R. 184* (MEXU); Ojocaliente, Predio La Lomas, 2 Oct 1987, *Silva R, L. 187* (MEXU).

**NETHERLANDS ANTILLES**. **Curaçao**: sin. loc., Oct 1968, *Arnoldo-Broeders 3621* (DUKE).

**PARAGUAY**. **Boquerón**: Filadelfia, 8 Jun 1983, *Hahn 1420* (BH); Río Verde, 26 May 1993, *Mereles 5109* (CTES, FCQ); Fuerte Olimpo, 30 Oct 1946, *Rojas 13678* (W); Colonia Fernheim, Estancia Laguna Porá, 1 Mar 1991, *Vanni et al. 2620* (CTES); **Central**: Estero del Ypoá: Puerto Guyratí on Paraguay River, 22 Jun 1993, *Zardini 36391* (AS, MO, SI). **Presidente Hayes**: Ruta Transchaco km 82, 8 Mar 1991, *Mereles 4010* (CAS, NY); km 65, Estancia Santa Maria del Doce Ret. San Juan, 1 Dec 2003, *Mereles et al. 9095* (FCQ); Estancia Santa Asunción, 12 Oct 2003, *Peña-Chocarro et al. 1483* (BM);

**Peru**. **Cusco**: Urubamba, Machu Picchu, 25 Jan 1975, *Schwabe s.n.* (B); **Lima**: Lima, *Cuming 1090* (BM, E, K); Lima, La Molina, Dec 1986, *Dreyfus s.n.* (USM); Lima, 1825, *Durville s.n.* (W); Lima, La Molina, 8 Nov 1986, *Vilcapoma 520* (USM).

**Puerto Rico**. Ensenada, 28 Jun 1963, *Liogier 9732* (DUKE, NY); Barrio Llanos Costa, Rancho Cabassa, on S. side of Sierra Bermeja, 16 Nov 1994, *Proctor* & *Colón 49664* (MO); Sabana Grande, 4 Feb 1935, *Sargent 513* (MO); Lajas, along road 324 several miles east of its intersection with road 304, 28 Jun 1965, *Stimson 1403* (DUKE, MO); Punta Jorobado, rte 235, south of Ensenada, 17 Jul 1968, *Wagner 1559* (DUKE); Ponce, 7 Sep 1979, *Woodbury & Del Llano s.n.* (MO, NY); Guanica, Ensenada, 19 Jun 1969, *Wagner 1880* (BM, DUKE);

**United States of America**. **Arizona**: Yavapai County, Ashfork, 10 km W on road to Seligman, 27 Sep 1985, *Bartholomew et al. 2430* (CAS, MEXU); Cochise County, Paradise, Chiracahua Mountains, 27 Sep 1907, *Blumer 1732* (DS, K, NY, W); Mohave County, Kingman, 3 miles SE of Kingman in foothills of Hualapai Mountains, 7 Sep 1961, *Breedlove 1150* (DS); Pima County, Twin Buttes, Sierrita Mountains, NW of Placer Peak, 25 Jun 1981, *Butterwick* & *Hillyard 7782* (CAS); Pinal County, Bloody Tanks Wash, near Miami, 11 Jun 1982, *Butterwick* & *Mittleman 8217* (CAS); Cochise County, Warren, Country Club S of Warren, Sulphur Springs Valley, 7 Jun 1915, *Carlson s.n.* (CAS); Yavapai County, Verde River, ca. 0.5 mile NE of Clarkadale [Clarkdale], 14 Apr 1960, *Crosswhite 720* (K); Santa Cruz County, route 82 toward Patagonia. 5 miles past N of Nogales, 12 Oct 1979, *Davis 1000* (BM, CAS, MEXU); Apache County, Springerville, Hwy 666, 3 miles S of P.O, 11 Jul 1963, *Deaver 6439* (CAS); Santa Cruz County, Huachaca Military Reservation, inside west gate, 21 Jul 1969, *Dreyer s.n.* (CAS); Maricopa County, Wadell, ca. 1/4 mile N of Wadell, or 14 miles NW of Peoria, 17 Apr 1960, *Dullas 135* (K); Gila County, Roosevelt Dam, Apache trail and adjacent regions, 22 May 1929, *Eastwood 17097* (CAS); Yavapai County, Camp Verde, 4 miles ESE on main road, 24 Jun 1979, *Ertter* & *Strachan 2939* (CAS); Adamana, 1954, *Ewan 15* (BM); Pima County, below White House Canyon, Santa Rita Mountains, 16 Apr 1928, *Graham 3619* (DS); Apache County, Canyon de Chelly National Monument, Antelope House Ruins, Canyon del Muerto, 29 Jul 1971, *Halse 568* (ARIZ); Coconino County, Grand Canyon National Park, Havasu Canyon, 27 May 1950, *Howell 26563* (CAS); Monroe, 22 Jul 1921, *Jones s.n.* (UC); Cochise County, San Pedro Riparian National Conservation Area, Upper San Pedro River floodplain, Escalante Crossing, near northern border of SPRNCA, 15 Jul 2002, *Makings 1102* (MEXU); Yuma County, Gila Mountains, Mt. Ord, at base of Mt. Ord Road (FSR 636) in paved area where road widens to allow parking, 22 Oct 2005, *Price 338* (MEXU); Pima County, Fort Lowell, 16 May 1894, *Price s.n.* (DS); Maricopa County, Tempe, corner of 8th Street and Van Ness, parking next to street, 19 Apr 1963, *Sprankle 96* (DUKE); Pinal County, Sacaton, 13 Jul 1928, *Thackery 226* (DS); 10 mi W on rt. 80 from Douglas, 12 Jun 1974, *Tilton 374* (CORD, MO); Navajo County, Holbrook, 16 Jun 1901, *Ward s.n.* (K, NY); Santa Cruz County, Coronado Forest, 0.6 km S of Yellow Jacket Road junction, 29 Jun 1995, *Way et al. WSSB- 3* (K); Maricopa County, Glendale, 1 mile W of Glendale, 21 Apr 1960, *White 6* (K); Pima County, Tucson, 3 miles SW of Tucson, 4 Jul 1928, *Wolf 2485* (CAS, DS); Cochise County, Douglas, 42 miles NE on road to Rodeo, New Mexico, 7 Jul 1928, *Wolf 2558* (CAS, DS). **Arkansas**: Monroe County, Brinkley, 28 Aug 1934, *Demaree 10857* (DS); Cleveland County, New Edinburg, 4 Jul 1938, *Demaree 17916* (CAS, DS). **California**: Los Angeles County, San Fernando Valley, near Cahuenga Pass, 17 Jul 1917, *Abrams 6607* (DS, NY); Yuba County, Honcut, Glen Clark Farm, 25 Aug 1969, *Ahart s.n.* (CAS); Tulare County, Sultana, at Santa Fe railway crossing on outskirts of town, 29 Jul 1941, *Bacigalupi et al. 2663* (DS); Sonoma County, Joe Kohuke Place at mouth of Adobe Canyon, 24 Oct 1945, *Baker 11244* (CAS); Contra Costa County, Pittsburgh, hiway S of Pittsburgh, 3 Sep 1946, *Baker 11574* (CAS); Sacramento County, Sacramento valley and south, 24 Sep 1931, *Ball s.n.* (CAS, DS); Sutter County, Yuba City, 5 miles W along road, 13 Aug 1965, *Bennett 8486* (DS); Kern County, just N of the jct between Hwy 58 and Bealville Road, on road to Caliente, between Bakersfield and Tehachapi, 17 Jun 1989, *Charlton 3259* (MEXU); Yolo County, Davis, 3 miles N along Southern Pacific Railroad, 2 Aug 1964, *Crampton 7136* (CAS); Solano County, railroad track between Suisern and Van Den, 28 Aug 1920, *Eastwood 1040* (CAS); San Bernardino County, Needles, 23 Jun 1916, *Eastwood 5958* (CAS); Los Angeles County, West Los Angeles, 25 Aug 1932, *Ewan 680* (BM, CAS, DS, DUKE, K, NY); Amador County, Jackson, Don Favre property, Previtali Road, Sierra Nevada, 15 Jul 1968, *Farnham s.n.* (CAS); Glenn County, Feenstra Farm, N side of Newville Road between Rd. FF and Rd. E, ca. 2 miles W of Orland, 27 Jul 1987, *Feenstra s.n.* (CAS); Riverside County, E side of Hwy 95, W bank of Colorado River, 2.1 miles N of Palo Verde Dam Road, 22.4 miles W of San Bernardino County line, 3 Oct 1962, *Fuller 9706* (CAS); Imperial County, Bard, 3.1 miles S of Bard, W of San Pasqual School, 17 Oct 1962, *Fuller 9796* (CAS); Calaveras County, Wallace, W limits, N side of Hwy 12, Sierra Nevada, 23 Jun 1970, *Fuller 19758* (CAS); Imperial County, Salton Sink, Brawley, 29 Jun 1965, *Goeden* & *Ricker s.n.* (CAS); San Luis Obispo County, El Pomar, 7 miles E of Templeton, Rienitz Ranch, 8 Aug 1957, *Hardham 2804* (CAS); San Francisco County, San Francisco, Embarcadero, 1956, *Howell s.n.* (CAS); Tehama County, Red Bluff, 6 miles S of Red Bluff, 17 Jun 1934, *Howell 12238* (CAS); Monterey County, King City, 21 Jun 1963, *Howell 39457* (CAS); Mariposa County, White Rock Road, 6 miles SW of Westfall Road, 22 Sep 1971, *Johnson* & *Keffer s.n.* (CAS); Inyo County, Haiwee, 17 May 1935, *Kerr s.n.* (CAS); Merced County, Ingomar, 30 Jul 1948, *Mason* & *Smith 8256* (DS); Contra Costa County, Pittsburgh, 2 miles W of Pittsburgh, 3 Sep 1946, *Mason* & *Grant 13097* (DS); Imperial County, El Centro, 2 miles E of El Centro, 30 May 1917, *McGregor 857* (DS); Mendocino County, Boonville, 9 Jul 1934, *Ornbaum s.n.* (CAS); Santa Barbara County, Santa Barbara, Southern Pacific Railroad W of town, 13 Sep 1953, *Pollard s.n.* (CAS); Ventura County, Foster Park, W side of Ventura Avenue (freeway) about 0.5 mile S of Foster Park, 9 Jul 1971, *Pollard s.n.* (CAS); Santa Barbara County, Santa Barbara, South Pacific Railway S of Samarkand Hotel, 24 Jul 1957, *Pollard s.n.* (W); Yolo County, Merritt Island, 3.5 miles S of Clarksburg near junction of county roads 140 & 142, 18 Aug 1969, *Quick 69-15* (CAS); San Bernardino County, Cajon Pass, just below Cajon Campground, 17 Sep 1961, *Raven 16690* (CAS); Mariposa County, Coulterville, 4.4 miles W on N side of Hwy 132, 22 Sep 1971, *Roberson* & *Ferlatte s.n.* (CAS); San Bernardino County, Yucaipa, between Ave E and F, east from Wilson Creek channel to top of slope, 4 Oct 1996, *Sanders 19612* (CAS); Riverside County, The Badlands, west fork of Laborde Canyon near old rocket test facility, 26 Apr 2002, *Sanders et al. 25026* (CAS); Tulare County, Prixley Wildlife Sanctuary, 15 Oct 1970, *Shevock 502* (CAS); Kern County, along California Highway 178 just W of Kern River Canyon entrance, 9 Jul 1994, *Shevock 12209* (CAS); San Mateo County, East Palo Alto, 779 Bell Street, 1 Sep 1962, *Thomas 9978 a* (DS); Tulare County, Angiola, 0.25 mile N of Angiola, 13 Jul 1955, *Twisselmann 2248* (CAS); Kern County, Devil’s Den region, Twisselmann Road 1.5 miles W of Highway 33, Western San Joaquin Valley, 10 Nov 1963, *Twisselmann 9088* (CAS); Kern County, Jerry Slough, at the Tracy Ranch (US Public Health study area), San Joaquin Valley (E side of Kern County), 22 Aug 1968, *Twisselmann 14887* (CAS); Monterey County, Spence, south of Salinas, 19 Oct 1943, *Wheeler 5859* (BM); Riverside County, Hemet, at NW corner of Stetson Ave and Stanford intersection, 22 Sep 1996, *White 4526* (CAS); Tehama County, Red Bluff, Jun 1917, *Wickes s.n.* (CAS); Riverside County, Blythe, field at edge of town, Palo Verde Valley, 28 Apr 1932, *Wolf 3086* (DS); Monterey County, Lewis Creek, 4.5 miles into Lewis Creek, 13 Sep 1984, *Yadon s.n.* (CAS). **Colorado**: Mesa County, public right-of-way, along a fence-line just S of Patterson Road near 27 1/2 Road, 22 Aug 1998, *Austin 277* (MESA); El Paso County, E of Fountain in a valley on old RR grade, 12 Jul 1935, *Christ 991* (CS); Baca County, Comanche National Grassland, at junction of County Road M and County Road 13, ca 16 mi S of Pritchett, 4 Oct 2003, *Elliot 11929c* (RM); Fremont County, E edge of Florence, 4 Jun 1955, *Harrington 796* (CS); Huerfano County, Wet Mountains, Wet Mountain Valley, Sangre de Cristo Range, and vicinity; County Road 330, 1.5 road mi S of Walsenburg (where road and railroad parallel), 28 Jul 1999, *Hartman 65234* (RM); Las Animas County, Comanche National Grassland, along Purgatoire River Valley near Rourke Ranch, ca 28 air mi SSW of La Junta, 13 Jul 2007, Kuhn et al. 2605 (RM); Bent County, near Las Animas, 4 Aug 1954, *Zonitch s.n.* (CS). **Florida**: Escambia County, Pensacola, 30 Jun 1892, *Curtiss 5913* (K, NY); Monroe County, Newport, Key Largo, 26 Mar 1898, *Pollard et al. s.n.* (BM); Monroe County, Key West, 30 Nov 1913, *Small* & *Small 4841* (K, NY, W). **Illinois**: Chicago [cultivated?], 3 Aug 1897, *Umbach s.n.* (CAS). **Indiana**: Lawrence County, Anderson Farm belonging to Purdue University, 5 miles west of Bedford, 6 Jul 1939, *Kriebel 8208* (DUKE). **Kansas**: Meade County, Meade, 13 Jul 1950, *Horr 3521* (DUKE); Sumner County, South Haven, 3 miles E of town, 19 May 1967, *Stephens 10953* (DS). **Louisiana**: Richland Parish, beside La. 183, 1.1 miles N of Holly Ridge and US 80, 24 Jul 1980, *Dixon 3636* (MEXU); Calcasisu Parish, Lake Charles, 18 May 1915, *Palmer 7678* (CAS, K); Boissier Parish, at edge of Bodcau Wildlife Management Area southeast of La. 160 and Ivan, Sec. 4, T20N, R11W, 29 May 1986, *Thomas* & *Dorris 96112* (BM). **Mississippi**: De Soto County, on Red Banks Rd 2.8 mi S of the junction of this road and US Hwy 78, 2 Jul 1969, *Ferrari 313* (MMNS); Washington County, 3 mi ESE of Leland, farm field by Bogue Philia Creek, 30 Aug 1994, *MacDonald 7551* (MMNS); Rankin County, on Reese’s Farm in Old Byram community, field surrounding Stumps Lake, 24 May 1975, *Snow s.n.* (MMNS); Lafayette County, ca. 2 mi N of a point off College Hill Rd, ca. 2 mi NW of College Hill, 19 Jun 1967, *Temple 5513* (MISS, MMNS); Wilkinson County, along county blacktop 2.8 mi. E of a point 9.4 mi. S of Adams-Wilkinson county line on U.S. Hwy. 61, 8 Aug 1969, *Temple 12151* (MMNS). **Missouri**: Barry County, sin. loc., 25 Jul 1893, *Bush 266* (K); Howard County, along valley of Missoury[i] River, north of quarry, 2 1/2-2 3/4 mi. north of Lisbon, 9 Oct 1956, *Steyermark 83071* (BM). **Nevada**: Nye County, Ash Meadows, SE Ash Meadows, US Atomic Energy Commission’s Nevada Test Site, Amargosa drainage basin, 17 Jun 1969, *Beatley 9028* (DS); Clark County, Overton, 6 May 1941, *Clokey 5937* (CAS, DS); Clark County, Las Vegas, NW limits of Las Vegas, Hwy 95 1 mile SE of Decatur Lane, 10 May 1962, *Fuller 8567* (CAS); U. S. Highway 95, 7 mi north of Searchlight, 7 Jul 1952, *Gullion 383* (CORD); Lincoln County, Dray Lake Valley, ca. 2 mi by air SW of intersection with Bennet Pass Road on Black Canyon power line road, 15 Jun 2013, *Gust 2206* (ENLC); Clark County, Moapa, Warm Springs, 24 May 2012, *Johnson 12-029* (BRY); Clark County, Kyle Canyon, leaving canyon, Spring Mountains, 8.2 miles from Hwy 95, 30 Jul 1979, *Williams 79-176 1* (CAS). **New Mexico**: Luna County, mesa at base of Little Florida Mountains, 24 Jul 1919, *Abrams s.n.* (DS); Eddy County, White’s City, 5 miles N of city, 17 Jun 1965, *Barbour 119* (DUKE); Otero County, along Rt. near Rio Tularosa at west base of Sacramento Mt. front, 8 miles N.E. from Tularosa, 4 Aug 1965, *Bennet 8659* (BM); San Miguel County, Las Vegas, vicinity, Oct 1919, *Brother Anect 115* (CAS); Eddy County, Hope, Jun 1907, *Campbell s.n.* (CAS); San Miguel County, Soham, Foothills Ranch, 30 Jun 1958, *Clear s.n.* (CAS); Grant County, Silver City, 8 May 1919, *Eastwood s.n.* (CAS); Hidalgo County, Lordsburg, 15 May 1919, *Eastwood 8550* (CAS); Santa Fe County, Glorieta, 12 Oct 1928, *Eastwood 15419* (CAS); Bernalillo County, Sandia Mountains, on road from Santa Fe to Albuquerque, 16 Oct 1928, *Eastwood 15646* (CAS); Socorro County, San Antonio, 21 Jun 1921, *Ferris* & *Duncan 2302* (CAS, DS); Dona Ana County, Mesquite, 28 Jul 1930, *Fosberg S-3399* (CAS, W); valley of Rio Grande, two miles north of Socorro, 3 Jul 1927, *Goddard 836* (BM, K); Dona Ana County, Las Cruces, bank of Rio Grande River S of Las Cruces, 17 Jun 1930, *Goodman* & *Hitchcock 1142* (CAS, DS); San Miguel County, Las Vegas Hot Springs, 30 May 1903, *Grant 5550* (DS); Lea County, Paduca Habitat Evaluation Area, 31.7 air miles SW of Hobbs, 21 air miles WSW of Eunice, at old oil well drilling pad, 14 Aug 2009, *Hansen 4421* (W); Doña Ana County, banks of Rio Grande near Doña Ana, 1 Aug 1958, *Hawkes et al. 1166* (K); Santa Fe County, Canoncito, 18 Jun 1897, *Heller* & *Heller 3733* (BM, DS, E, G, K, NY); Sandia County, Albuquerque, 3 Sep 1884, *Jones 4119* (BM, CAS, G); Dona Ana County, Jornada del Muerto, base of Mt. Summerford, 8 Jun 1983, *Lajtha 123* (DUKE); Colfax County, Vermejo Park Ranch, fields along south side of Ponil Creek at Cimarron Headquarters on northeast side of Cimarron, 15 Aug 2008, *Legler 10779* (RM, UNM); Grant County, Dog Spring, 27 May 1892, *Mearns 122* (DS, NY); Luna County, Carrizalillo Mountains, 15 Apr 1892, *Mearns 135* (DS); Grant County, Mangas Springs, Aug 1901, *Metcalfe s.n.* (DS); Grant County, Santa Rita, 8 Aug 1895, *Mulford 684* (K); Grant County, Lordsburg, 15 miles E of Lordsburg, 4 Sep 1938, *Rollins* & *Chambers 2749* (DS); San Miguel County, Pecos Indian Ruins, Santa Fe National Forest, 21 Jul 1937, *Sagalyn 69* (DUKE); Lincoln County, Gray, 1 Jun 1898, *Skehan 16* (K, W); Guadelupe County, Santa Rosa, 20 miles W at junction of US 84 and 66, 20 Jul 1958, *Smith 1030* (CAS); Grant County, Pinos Altos, mountains near Pinos Altos, 26 Jun 1936, *Stewart s.n.* (CAS); Union County, 7 mi. SW of Logan, 7 Jul 1949, *Stiteler s.n.* (BM); San Miguel County, 75 mi W of Roswell, , 11 Jun 1974, *Tilton 321* (CORD, MO); San Miguel County, 3 mi W of Alamogordo, NM on rt. 82 toward Las Crucas, 11 Jun 1974, *Tilton, D. 330* (CORD, MO); Dona Ana County, Mesilla, 28 Jun 1897, *Wooton 59* (DS, E, G, K, NY); Dona Ana County, Mesilla Valley, 10 Aug 1907, *Wooton* & *Standley 3127* (DS). **North Carolina**: Dare County, Nag’s Head, along US 158 business, northern part of Nag’s Head, 30 Jul 1970, *Leonard* & *Radford 3394* (MEXU, NY). **Oklahoma**: Cleveland County, Norman, 2 Aug 1924, *Bruner s.n.* (DS); Creek County, Sapulpa, “I. Ten.” (Plants of Indian Territory), 19 Jun 1894, *Bush 392* (K); Beckham County, north side of North fork of Red River about 6.5 miles N of Texola, 9 Jun 1940, *Clausen 4597* (K, NY); Comanche County, Fort Sill, 14 Jun 1916, *Eastwood 11763* (CAS); Cleveland County, Norman, Dec 1944, *Hopkins et al. 897* (DS); Custer County, 1 mile north of Weatherford, Jul 1938, *Mericle 500* (BM, CAS, DS, DUKE, K); Alfalfa County, Aline, 8 Jun 1913, *Stevens 802* (DS, K); Dewey County, Putnam, 11 Jun 1913, *Stevens 889* (SI); near Oklahoma City, 16 Sep 1938, *White 1180* (MEXU). **Oregon**: Umatilla County, Hermiston, Jul 1945, *Hachler s.n.* (OSC). **South Carolina**: Aiken County, corner of Ridgecrest Avenue and Martintown Road in : North Augusta, 9 Jun 1962, *Ahles* & *Baird 56834* (BM, E, NY). **Texas**: Reeves County, Balmorhea, 4 mi S of Balmorhea on Texas 17, 4 Sep 1966, *Anderson* & *Laskowski 3504* (DUKE); Bexar County, Bejar [Presidio San Antonio de Bexar], *Berlandier s.n.* (K); Jim Hogg County, Hebronville, 26 miles SW on Farm Road 496, 27 Mar 1965, *Botello* & *Ayala 14* (DS); La Salle County, Encinal, US Hiway 81, 7 miles N of Encinal, 27 Oct 1962, *Bruno 4* (DUKE); Nolan County, Sweetwater, 5 May 1925, *Burkill 432* (K); Brazoria County, Columbia, [West Columbia], 20 Oct 1900, *Bush 1588* (K); Grimes County, College Station, 13 miles E of city on Hwy 30, 3 May 1974, *Calhoun 129* (CAS); El Paso County, Fort Bliss, 30 Apr 1915, *Carlson s.n.* (CAS); Edwards County, Substation No. 14, 5 Oct 1945, *Cory 52468* (DS, NY); Zapata County, Zapata, 2 miles SE of Zapata, 25 Apr 1965, *Cuesta 57* (DS); Travis County, Austin, junction Texas 270, Texas 271, 5 Sep 1978, *D’Arcy 11689* (MEXU); Lubbock County, Lubbock, Texas Tech. campus, 13 May 1930, *Demaree 7674* (DS); Jeff Davis County, Fort Davis, 19 Sep 1920, *Eggleston 7424* (BM); Harris County, Houston, 21 Jul 1917, *Fisher 5089* (CAS); San Patricio County, on N side of Nueces Bay, west of Portland, near junction of county rds. 66 and 67, 22 Apr 1998, *Fryxell 5131* (MEXU); Zapata County, Mecom Ranch, hiway 83, 6 miles S of San Ignacio, 15 Mar 1964, *García 23* (DUKE); Grayson County, Denison, edge of town, 20 Jun 1949, *Gentry 51-393* (MEXU); Palo Pinto County, Mineral Wells, 8 Jun 1931, *Gillespie 5222* (DS); Zapata County, Laredo, 22 miles S on US Highway 83, 4 Apr 1965, *Guerra et al. 601* (DS); Waller County, Hempstead, 10 Jun 1872, *Hall 498* (BM, G, K); Nueces County, Corpus Christi, 23 Mar 1894, *Heller 1511* (E, G, K); Bowie County, near Texarkana, 31 Aug 1898, *Heller* & *Heller 4185* (E, G); Presidio County, Chinati Mountains State Natural Area, along jeep trail W of Cienaga, SW of Sierra Parda, 4 Aug 2004, *Lott et al. 5121* (CAS); Presidio County, Chinati Mountains State Natural Area, NE- to SW-trending tributary to San Antonio Canyon, below old San Antonio Mine, 21 Aug 2005, *Lott et al. 5573* (CAS); Willacy County, San Perlita, 8 May 1940, *Lundell* & *Lundell 8784* (DS); Bexar County, San Antonio, Our Lady of the Lake College campus, 29 Oct 1939, *Metz 3164* (CAS); Webb County, Laredo, 29 May 1974, *Nee 11787* (K, MEXU); Travis County, Austin, west side of Austin, along Red Bud Road just W of Colorado River, 30 May 1974, *Nee* & *Whalen 11815* (MEXU); Hidalgo County, near entrance to Bentsen-Rio Grande State Park, 4 mi SW of Mission, 18 Dec 1981, *Nee 24064* (CORD, F); Parker County, 8.3 km NW of center of Weatherford, Shady Grove Road, 1.3 km N of Old Garner Road, 22 May 2010, *Nee 57070* (MEXU, NY); Tarrant County, along Long Ave. at Braswell Drive, 1/4 km W of I-35 W, 6 km NNE of center of Fort Worth, 26 May 2010, *Nee 57083* (NY, SI); Terrell County, Sanderson, 26 Jun 1924, *Orcutt 680* (DS); Taylor County, Abilene, Aug 1883, *Parish s.n.* (DS); Dallas County, Dallas, Jun 1880, *Reverchon s.n.* (G); Frio County, Dilley, US Highway 81, 2 miles N of Dilley, 22 Mar 1964, *Rodríguez 43* (DUKE); Victoria County, Victoria, 16 Sep 1908, *Schallert 540* (DUKE); Washington County, Millerick Settlement, 1856, *Schlottmann 269* (LE); Atascosa County, ‘southern Atascosa County’, 20 Apr 1920, *Schulz 97* (CAS); Maverick County, 5 miles N of Normandy, 10 Sep 1962, *Scora 2285* (MEXU); Jeff Davis County, Valentine, 0.5 miles S of Valentine, 18 Aug 1953, *Selander 21* (DS); Kinney County, Brackettville, 4 miles NE of town, 15 May 1965, *Strother 258* (DS); Hidalgo County, about 5 miles W of Mission, in railroad cut, 30 Apr 1949, *Tharp* & *York 51- 261* (MEXU); Wichita County, 10.6 mi outside Wichita Falls on route 82 W to Lubbock, 9 Jun 1974, *Tilton 218* (CORD, MO); Baylor County, 21 mi W of Seymour on rt. 82 (roadside has been moved very recently from Seymour to Vera), 9 Jun 1974, *Tilton 237* (CORD, MO); Taylor County, Camp Barkeley, 1 Nov 1942, *Tolstead 5859* (W); Galveston County, Galveston Island, 23 Sep 1901, *Tracy 7576* (BM, CAS, W); Chambers County, Wallisville, ca. 1/4 mile S of Tex hwy 73, ca. 1/8 mile N of Trinity River, along road toward river, S side of road, 14 Jul 1958, *Traverse 802* (MEXU); Kaufman County, Mabank, on highway 175, 1/4 mile E of the second bridge over the reservoir, 5 Jun 1996, *Whitson* & *Whitson 803* (DUKE); Kimble County, on West I-10 at the Fort McKavett overpass, 41 miles E of Sonora, 16 Jun 1996, *Whitson* & *Whitson 817* (DUKE); Cameron County, Brownsville, near the Western Union Station on St. Charles Street, 14 Jul 1984, *Wilbur 35189* (DUKE); Hale County, about 3 miles N of Abernathy, near small lake, 25 Sep 1948, *York* & *Cowan 52-300* (MEXU); Lubbock County, Lubbock, 2 May 1934, *Zobel s.n.* (CAS). **Utah**: San Juan County, 2 mi E of Bluff, 21 May 1985, *Atwood 11023* (NY); Washington County, near Zion National Park, Jun 2006, *Bohs s.n.* (UT); Kane County, Kaiparowits Plateau, south side of US Highway 89 ca. 1 mile east of The Cockscomb, 15.5 miles west of Big Water, 7 Jun 2006, *Fertig 22568* (NY); Wshington County, St. George, 12 Nov 1999, *Higgins 20971* (NY); San Juan County, Glen Canyon NRA, San Juan River mile 44, Honaker Trail, 16 Jun 2003, *Hill 225* (BRY); Tooele County, Open Reventment Area, 3 Aug 2000, *Long 1100* (BRY, RENO). **Washington**: Asotin County, Snake River Canyon, 5 miles upstream (SE) from intersection (in Asotin) w/ Hwy 3 at east end of town, and ca ½ mile SSE of confl[uence] of Snake River and Tenmile Cr. along road from Asotin to Heller Bar, 21 Aug 1983, *Henderson & Cholewa 6848* (NY).

**Uruguay**. **Colonia**: Colonia, 6 Jan 1902, *Berro 7942* (G). **Río Negro**: Fray Bentos, 5 Feb 1879, *Felippone 5092* (K). **Soriano**: Villa Soriano, 24 Jan 1908, *Berro 4411* (G).

#### 2. Solanum hindsianum

Taxon classificationPlantaeSolanalesSolanaceae

Benth., Bot. Voy. Sulphur 39. 1844.

Figures 2C , 7

##### Type.

Mexico. Baja California: Bay of Magdalena [Bahía Magdalena], [1841], *R. Hinds s.n.* (holotype: K [K000063726]).

**Figure 7.**
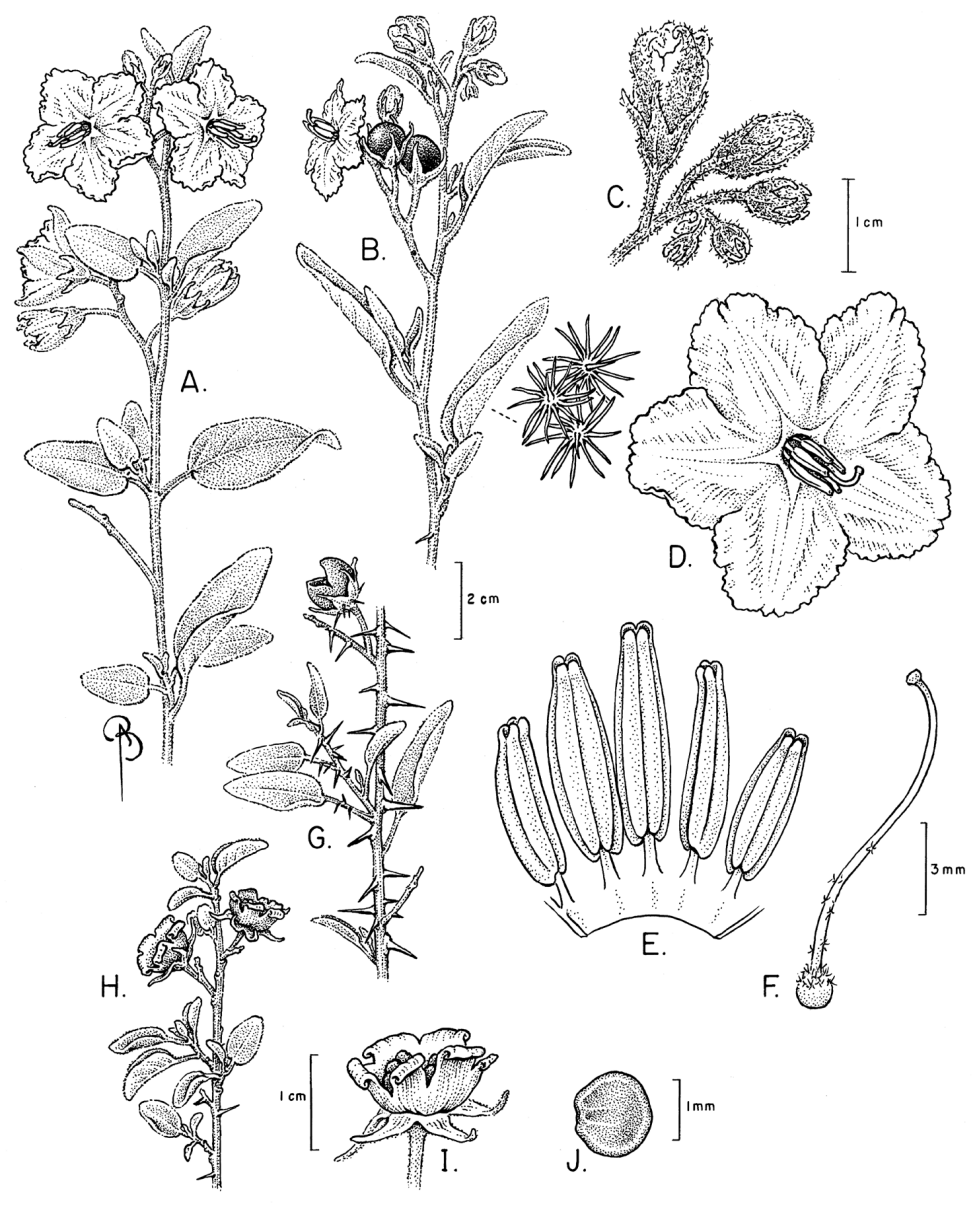
*Solanum hindsianum* Benth. **A** Flowering branch **B** Fruiting branch showing stellate trichomes **C** Buds **D** Corolla **E** Anthers **F** Gynoecium **G** Fruiting branch of prickly individual with broad based stem prickles **H** Fruiting branch with mature berries **I** Mature berry with irregular dehiscence **J** Seed. Drawn by Bobbi Angell. **A, D–F** from *Halse s.n.* (NY00854332) **B, C** from *Sousa-Peña 79* (NY00751522); G from *Orcutt 1339* (NY00751531); **H, J** from *Daniel 1856* (NY00751517).

##### Description.

Erect shrub, 0.5-3 m tall. Stems armed or more often unarmed; young stems pale yellowish green, densely stellate-pubescent with sessile to short-stalked porrect-stellate trichomes with 8–12 rays (0.03-)0.1–0.2 mm long, the midpoints up to 0.07(0.1) mm long, often reduced; prickles if present 0.2–1.5 cm long, 0.2–2 mm wide at base, usually straight, reddish brown, bark of older stems smooth, brown or greyish or reddish brown, glabrescent. Sympodial units difoliate, not markedly geminate. Leaves simple, (1-)2–3 cm long, (0.5-)0.8–2.3(-6) cm wide, ovate to elliptic (unusually narrowly elliptic), usually concolorous, drying yellowish green to pale green, densely pubescent on both sides and the blade surface not visible, the pubescence usually more dense beneath, the trichomes porrect-stellate, sessile or short-stalked, translucent, the rays 8–14, 0.05–0.2(0.3) mm long, straight, slightly fused near the midpoint base, the midpoint to 0.1 mm, often absent; primary veins 4–7 pairs, raised adaxially, flat abaxially, spreading at ca. 45° to the midvein, the tertiary venation mostly obscured by the dense stellate pubescence; base rounded to truncate, usually oblique; margins entire, rarely shallowly lobed, if so the lobes 2–3 on each side, the sinus extending only to 1/8 of the distance to the midvein, up to 3 mm long, rounded; apex obtuse or rounded; petiole 0.5–1.5 cm long, 1/3–1/4 of the leaf length, densely stellate-pubescent like the young stem. Inflorescences terminal or lateral, 3–4 cm long, unbranched, with 3–4 flowers; peduncle 1–2 cm long, rachis 0.5–1 cm long; pedicels 0.4–0.5 cm long, ca. 1 mm in diameter, filiform or apically dilated, densely stellate-pubescent like the leaf blade, unarmed, even in plants with prickly stems, articulated near the base; pedicel scars spaced 1–2 mm apart, forming prominent brown stumps. Buds ellipsoid, the calyx ca. ½ of the corolla length prior to anthesis. Flowers 5-merous, heteromorphic and the plants andromonoecious, the lower ones (1–3) hermaphroditic and long-styled and the more distal staminate and short-styled. Calyx tube 6–7 mm long, conical or cup-shaped, strongly keeled at the midvein, the lobes 2–3 mm long, 2–3 mm wide at base, long-triangular from a deltate base, densely stellate-pubescent abaxially, unarmed. Corolla 2.5–4(-5) cm in diameter, apparently expanding with age (slightly smaller in short-styled flowers), violet, drying yellowish-light brown, lobed for 2/3–1/2 of its length, pentagonal with abundant interpetalar tissue, the lobes 0.8–1.2 cm long, 1–1.2 cm wide, broadly deltate with an abruptly acuminate tip, spreading or slightly reflexed, densely stellate-pubescent abaxially where exposed in bud, the interpetalar tissue glabrous abaxially. Stamens equal or very slightly unequal, with the 2 adaxial anthers shorter than the 3 abaxial anthers; filament tube 1–1.5 mm long; free portion of the filaments 2–3 mm long, glabrous; anthers 6–10 mm long, free, slightly unequal, poricidal at the tips, the pores about the same diameter as the anther apices, clearly delineated, the anther surface smooth to finely papillose. Ovary globose, 2–3 mm in diameter, glabrous; style in long-styled flowers 13–18 mm long, glabrous, strongly curved, in short-styled flowers 5–6 mm long, straight or only slightly curved; stigma capitate in long-styled flowers, the surface minutely papillose. Fruit a globose berry, 1(-3) per infructescence, 1–1.5 cm in diameter when dry, the pericarp thin, smooth, glabrous, light green, sometimes with dark stripes or a marbled pattern when young, drying dark brown or brown-reddish, drying and cracking open to release the seeds when mature; fruiting pedicels 1–2 cm long, 1–2 mm in diameter at the base, 2–3 mm at the apex, woody, erect, usually unarmed; fruiting calyx not accrescent, up to 1.5 cm long, usually unarmed. Seeds ca. 10–30 per berry. 2–3 mm long, 2–3 mm wide, flattened reniform, golden to dark reddish brown, the surface minutely pitted, the margins thickened, the testal cells sinuate, more rectangular in shape near the margins. Chromosome number: n=12 (Averett and Powell 1972).

##### Distribution

**Figure 8.**
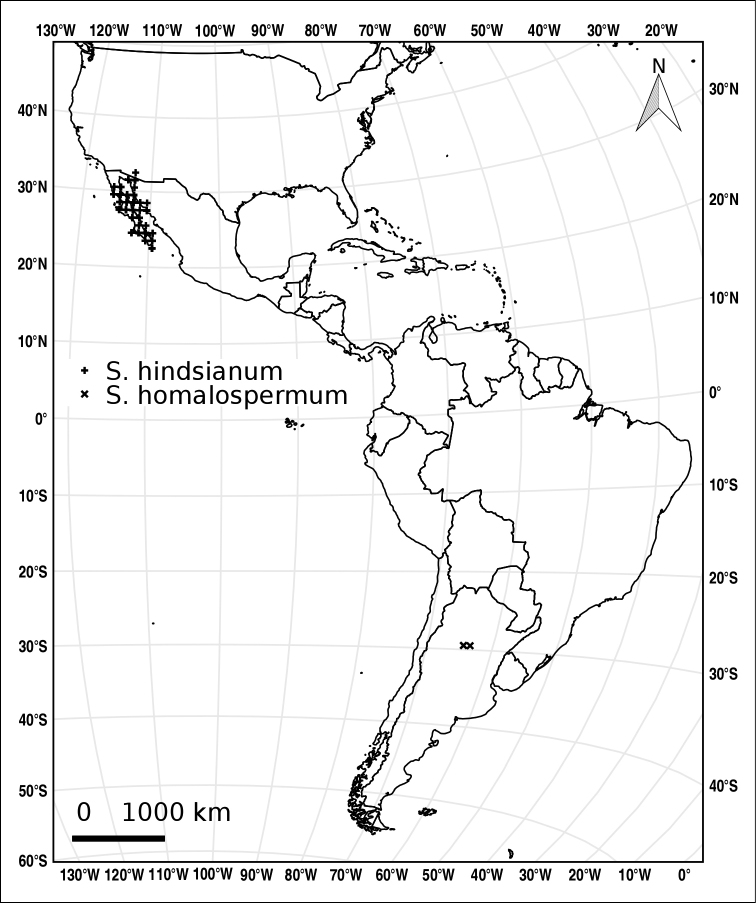
Distribution of *S. hindsianum* and *S. homalospermum*.

(Figure 8). *Solanum hindsianum* is endemic to the Sonoran Desert region of southern Arizona (Organ Pipe Cactus National Monument) and northern Mexico (States of Baja California, Baja California Sur, Sonora and Sinaloa); it occurs in matorral xerófilo, bosque tropical caducifolio and bosque espinoso (classification of Rzedowski 1978), from sea level to 400 m.

##### Ecology and habitat.

In the Sonoran Desert biome (Pinkava et al. 1992), *S. hindsianum* is most often recorded as growing in rocky outcrops and scrubby areas, coastal scrub (matorral) in rocky areas and dunes near the shore.

##### Common names and uses.

Mexico. Baja California: mariola (many collections); Baja California Sur: mariola (many collections), trompillo (MEXU-1203067), malva (*Ramirez 33*); Sonora: mariola (*López 85*). United States of America. Arizona: Hinds’ nightshade, Baja nightshade (in cultivation).

##### Preliminary conservation status

(IUCN 2016). LC (Least Concern). EOO 315,043 km^2^ (LC – Least Concern); AOO 544 km^2^ (VU- Vulnerable). *Solanum hindsianum* is widespread throughout Baja California and along the coast of the Gulf of California, and although it is habitat restricted, it appears to be common where it occurs.

##### Discussion.

*Solanum hindsianum* is similar to the widespread *S. elaeagnifolium*, but has larger, more pentagonal flowers with a curved style and somewhat dimorphic anthers (although not as dimorphic as those in *S. houstonii*). Anderson et al. (2007) do not record the anthers of *S. hindsianum* as dimorphic, nor the flowers as zygomorphic. *Solanum hindsianum* is sister to *S. houstonii* (sometimes identified as *S. tridynamum*) in all molecular analyses to date (Anderson et al. 2007; Stern et al. 2011; Wahlert et al. 2014). *Solanum hindsianum* can be distinguished from *S. houstonii* by its less strongly zygomorphic flowers, its usually rounder, smaller leaves and its berries that are not completely enclosed in the accrescent calyx. In areas of sympatry in northwestern Mexico, populations of *S. houstonii* often have anthers that are purple or purple-tinged, while those of *S. hindsianum* have not been recorded as other than bright yellow, and populations of *S. houstonii* in these areas of sympatry often have deeply pinnatifid or lobed leaves (e.g., type of *S. azureum*).

*Solanum hindsianum* has entered the garden plant trade in the southwestern United States (e.g., http://www.desert-tropicals.com/Plants/Solanaceae/Solanum_hindsianum.html) where it is used in xeric landscaping. As a wild species in the United States it is only known from extreme southern Arizona in Organ Pipe National Monument (see Specimens examined).

##### Selected specimens examined.

**Mexico. Baja California**: Ensenada, Cataviña, 10 km N of Cataviña, 26 Feb 1991, *Breedlove* & *Burns 71604* (CAS, MEXU); Ensenada, San Agustin, 12 miles S, 29 Jul 1955, *Chambers 720* (DS, MEXU); Ensenada, Socorro, about 12 miles NE of Socorro, Distrito del Norte, 6 Feb 1947, *Constance 3116* (DS, K, MEXU); Punta Prieta, 15 miles SW of Punta Prieta, 10 Feb 1935, *Epling* & *Robison s.n.* (DS, K, NY); Mulegé, Santa Rosalia, 9 miles from Santa Rosalia on the San Ignacio road, 10 Mar 1934, *Ferris 8634* (DS); Bahia de Los Angeles, S of inner road, 13 Mar 1992, *Fritsch et al. 1325* (MEXU); Ensenada, afuera de la cueva de las Pinturas Rupestres de Cataviña, 6 May 2008, *García-Mendoza et al. 9016* (MEXU); Sierra Calavario, in eastern bajada of Sierra, systema de Sierra Viscaino (Desierto Viscaino Region), 10 Mar 1947, *Gentry 7502* (DS); Calmalli, 8 miles W of Calmalli, 8 Feb 1935, *Haines* & *Stewart s.n.* (DS, K); Mesquital, about 3 miles inland, 29 miles N of Mesquital, 27 Sep 1941, *Hammerly 75* (CAS, DS); Ensenada, 7.1 miles by road east of Rancho Laguna Chapala, 13 Oct 1963, *Hastings* & *Turner 63- 182* (DS); Turtle Cove, Magdalena Bay, type region, 10 Aug 1932, *Howell 10643* (CAS, DS, NY); San Fernando, 1 Mar 1958, *Huey s.n.* (W); Ensenada, Socorro, 2.4 miles N of Socorro, 21 Apr 1935, *Ingram* & *Chisaki 650* (DS, NY); Mexicali, Isla Angel de la Guarda, opposite Pond Island, 30 Jun 1921, *Johnston 4201* (CAS, K); Ensenada, 0.5 miles E of Hwy 1, 20 miles S of San Quintin, 24 Dec 1973, *Kipping 308* (CAS); Ensenada, Isla San Lorenzo Sur, cañon grande que da al S, lado SW de la isla, 6 May 1985, *Lott* & *Atkinson 2469* (CAS, MEXU); 5 mi S of San Quintin on Hwy 1, 17 Apr 1992, *Merello* & *Brunner 250* (MEXU); Manuela, ca. 6.4 miles S of Rancho de Mesquital, ca. 5.3 miles N of site of Manuela, 13 May 1983, *Michener et al. 4253* (CAS); San Juan Mine, Sierra San Borja, 24 Mar 1960, *Moran 8030* (DS); Arroyo de la Escopeta, head of arroyo, S slope, 3 Jun 1975, *Moran 22371* (CAS, MEXU); Aguascalientes, 2 Oct 1925, *Orcutt 2793* (DS); Sierra la Asamblea, SW foot of the range near the W edge of Mesa Yubay and the SSW side of Mesa Cuerno de Borrego, ca. 7 road miles NNE of the abandoned site of El Desengaño., 3 May 1993, *Ross et al. 7032* (BM, CAS); Isla Mellizas, norte, Bahía de Guaymas, 26 Sep 2006, *Suárez Gracida 2006- 25* (MEXU); Ensenada, San Simone, N of San Quintin, 22 Mar 1949, *Thomas 111* (DS); Arroyo just W of Highway 1 near gravel quarry, ca. 6.7 miles S of Socorro Wash (Arroyo Hondo), 23 Apr 1984, *Thorne et al. 58047* (MEXU); Ensenada, El Marmol, 6 Mar 1930, *Wiggins 4366* (CAS, DS, K, NY); Mexicali, San Felipe, 28 miles S of San Felipe, 4 Mar 1962, *Wiggins 16972* (DS); Arroyo Grande, side canyon of Arroyo Grande E of El Rosario, near end of road N of San Juan de Dios, 2 May 1993, *Wisura et al. 4843* (MEXU). **Baja California Sur**: Los Cabos, rancho Boca del Salado carretera costera Buena Vista-Los Cabos, 31 Oct 1983, *Agúndez 297* (MEXU); La Paz, Isla Espiritu Santo, Gulf of California, 15 May 1939, *Berry, E.O. 19* (CAS); Comondú, Bahia San Juanico, 8 Oct 1939, *Berry 34* (CAS, DS); Mulegé, northern Sierra La Giganta, S of Mulegé, 11.2 mi. W of Hwy 1 on road from Rosarito to San Isidro, 5 Apr 1991, *Boyd* & *Ross 5956* (MEXU); Mulegé, Puerto Nuevo, Sierra de Placeros, 10 Mar 1991, *Breedlove* & *Burns 71795* (CAS, MEXU); La Paz, a 2 km del entronque del a Colonia Tabachines, sobre la carretera de terraceria a la Colonia Oriental, por la carretera federal La Paz a Los Cabos, 23 Feb 2008, *Calzada 25037* (K); Mulegé, Arroyo San José de Castro, Desierto Vizcaino, 26 Jun 1983, *Cancino 20* (MEXU); La Paz, Ejido El Centenario, 16 km por ONO de La Paz por la carretera transpeninsular, 11 Jun 2001, *Carrillo-Reyes* & *Cabrera 1974* (MEXU); Mulegé, Tres Virgenes, at base of southernmost of Tres Virgenes, 25 miles W of Santa Rosalia on W side of pass, 3 Aug 1955, *Chambers 771* (DS, MEXU); along road between Hwy 1 and La Purisima, 9.5 km SW of Hwy 1, 11 Dec 1994, *Daniel* & *Butterwick 6784* (CAS, MEXU); La Paz, carretera por Pichilingue, 1 km por carretera a Puerto Carralvo desde la entrada en el estero Enfermeria, norte de La Paz, 12 Oct 2001, *Domínguez Cadena 2420* (MEXU); Mulegé, Rancho El Cuarenta, 29 km al SE de Laguna San Ignacio, 18 Oct 2009, *Domínguez Cadena 3063* (MEXU); Santa Rosalia, 0 Apr 1986, *Elizondo 319* (MEXU); Los Cabos, Rancho Cabo El Sol, 10 km al noroeste de Cabo San Lucas, 2 Aug 1984, *Encarnación* & *Agúndez 32* (MEXU); Isla Espiritu Santo, 1 km al O de la bahia, en frente del Islote del Gallo, 11 Jan 1987, *Flores-Franco 400* (MEXU); Isla Espiritu Santo, 12 May 1975, *Gaviño de la T. s.n.* (MEXU); La Paz, Isla Cerralvo, 10 May 1975, *Gaviño de la T. s.n.* (MEXU); Isla Monserrat, 19 May 1975, *Gaviño 13* (MEXU); Los Cabos, El Arco, 14 miles NE, 18 Nov 1938, *Gentry 4037* (DS,K); Desierto Vizcaino, Las Tinajas and vicinity in cerros east of Los Picachos de Santa Clara, 21 Mar 1947, *Gentry 7542* (DS); Mulegé, Arroyo de Tecolote, near lava flows, 19 Nov 1947, *Gentry 7863* (DS); Los Cabos, Cabo San Lucas, 6 Aug 1932, *Howell 10610* (CAS, DS); La Paz, Pescadero, S of Todos Santos, 4 Apr 1930, *Johansen 551* (CAS, DS); Los Cabos, San Juan del Cabo, 18 Jan 1923, *Jones 24004* (CAS,DS); Sierra Cacachilas, base del Cerro del Puerto de Los Soldados, 6 Feb 2003, *León de la Luz 10161* (MEXU); Sierra la Giganta, Llanos de Kakiwi, Rancho El Choyal, 19 Nov 2004, *León de la Luz 10591* (MEXU); a 30 km al NW de Ciudad Constitución, 10 Feb 1975, *López-Forment 292* (MEXU); La Paz, 12.2 mi NW of junction carretera Las Garzas on Hwy 1, 31 Aug 1985, *Luckow et al. 2837* (MEXU); Mulegé, 81 km entre Guerrero Negro y Santa Rosalia, 28 Nov 2004, *Martínez 6618* (MEXU); 5 mi N of San Ignacio on Mexican Highway 1, 30 Apr 1992, *Miller et al. 7291* (MEXU); San Juanico, 27 Mar 1952, *Moran 3518* (BH, BM, DS); Isla Cerralvo, south end, Gulf of California, 3 Apr 1952, *Moran 3603* (DS); Isla San Francisco, 11 Apr 1952, *Moran 3740* (DS); Isla San Jose, south end of Amortajada Bay, 11 Apr 1952, *Moran 3779* (DS); Aguaje de San Esteban, 5 Oct 1905, *Nelson* & *Goldman 7210* (BM); about 10 miles north of Loreto, 3 Feb 1977, *Reeder* & *Reeder 6772* (MEXU); Vizcaino Desert, mountain pass through the Sierra El Placer, 11.45 road miles S of the main Vizcaino-Bahia Tortugas road, along the road toward Cerro El Elefante and Bahia Asuncion, 1 May 1993, *Ross et al. 6995* (CAS); Isla San José, costa suroeste de la isla, a 20 m de la playa, 4 Nov 1986, *Sousa-Peña 79* (MEXU, NY); Isla Santa Catalina, lado SW de la isla, en una pequeña ladera a unos 500 m del mar, 6 Nov 1986, *Sousa-Peña, M. 102* (MEXU); Isla del Carmen, lado W de la isla, Puerto Balandra, a 100 m de la playa, 7 Nov 1986, *Sousa Peña 133* (CAS, MEXU); Isla Cerralvo, playa SW, Los Viejitos, cerca del Faro, 8 Nov 1986, *Sousa-Peña 175* (MEXU); Santa Rosalia, 31 km al SE de Bahía de Tortugas, brecha a Viscaino, 21 Apr 1987, *Tenorio L.* & *Romero de T. 1306* (MEXU); San Antonio, 4 km al SE de San Pedro, carretera a Cabo San Lucas, 13 Apr 1987, *Tenorio L.* & *Romero de T. 12824* (MEXU); Mulegé, El Arco, 4–5.5 miles southwestward (by road) from El Arco on road to La Banderita, 22 Oct 1959, *Thomas 8285* (CAS, DS, MEXU); Comondú, Cadaje, 1.5 mi S of Cadaje, 19 Dec 1959, *Wiggins* & *Ernst 596* (DS, MEXU); La Paz, 11 miles E of La Paz on road to Las Cruces, 27 Nov 1959, *Wiggins 15648* (CAS, DS, MEXU). **Sinaloa**: Mazatlán, 20 Nov 1926, *Jones 22514* (MEXU). **Sonora**: San Luis Río Colorado, Sonoyta, 11.4 miles S on Mexico 2, 26 Jan 1962, *Breedlove 1455* (DS); Isla Pájaros, Bahía de Guaymas, 7 Oct 2006, *Búrquez 2006- 89* (MEXU); El Himalaya, peña Blanca, faja costera entre San Carlos y Tastiota, 21 Mar 1991, *Búrquez 91-316* (MEXU); Guaymas, ca. 5 mi (8 km) N of San Carlos towards Playa del Mar, 17 Mar 1992, *Eggli et al. 1958* (MEXU); al E de Pinacate, 16 Apr 1981, *Equihua et al. s.n.* (MEXU); Cañón del Nacapule, ca. 6 km N of Bahía San Carlos, 13 Dec 1992, *Felger* & *Búrquez 92-1031* (MEXU); Isla San Esteban, 5 Apr 1963, *Felger 7071* (MEXU); Puerto Peñasco, pinacate region, ca. 4 km SSE of Tinajas de los Papagos, 26 Sep 1964, *Felger 10542* (MEXU); Guaymas, Ensenada Grande, (=Bahía San Pedro), bajada at N end of bay, 13 Nov 1964, *Felger et al. 11613* (MEXU); Isla Turners, (=Isla Datil), small island off shore of Isla Tiburon, NW side of island, 20 Dec 1966, *Felger* & *Cooper Miller 15319* (CAS); Hermosillo, Isla Tiburon, Tecomate, 24 Mar 1968, *Gold 365* (DS, MEXU); Pitiquito, Desemboque, 6 miles N (by road) of Desemboque, near Río San Ignacio, 29 Apr 1964, *Hastings, J.R.* & *Turner, R.M.*, *64- 51* (DS); Hermosillo, Tastiota, 19 Oct 1965, *Hastings* & *Turner 65-165* (DS); Hermosillo, Isla San Esteban, 19 Apr 1921, *Johnston 3178* (CAS); Pitiquito, Bahia Tepoca, 25 Apr 1921, *Johnston 3302* (CAS); Altar, Quitovac, 11.6 miles N of Quitovac, N of summit of Paso San Emeterio (just S of Sierra de Cubabi), ca. 17 miles S of Sonoyta, 13 Mar 1936, *Keck 4142* (CAS, K); Puerto Peñasco, Highway 2, 11 miles (17.7 km) by road S of Sonoyta, near where ridge of Sierra Cubabi approaches the road, 13 Apr 1992, *Levin 2159* (MEXU); Hermosillo, antes de llegar a Punta Sargento, al NO de Punta Chueca, 27 Mar 1987, *López 85* (MEXU); Empalme, despues de La Palma, ejido Santa Maria Morelos (La Atravezada) a 13 km por la carretera 85 del km 115 de la carretera 15, 23 Jul 1995, *Saucedo et al. 338* (MEXU); Bahia San Carlos, inland from bay, Guaymas, Cerro Otatal, 5 Apr 1964, *Sherwin 226* (DUKE); Guaymas, 27.4 km south of Restuarant Los Arrieros, 4.4 km north of Pemex El Caballo (toll road turnoff north of Guaymas) km 143 on Mex 15, 12 Jan 1998, *Van Devender et al. 98- 29* (MEXU); Sierra Pinacate, Campo Rojo (Palo Verde), E of Pinacate Peak, 21 Mar 1980, *Webster 24269* (MEXU).

**United States of America**. **Arizona**: Pima County, Organ Pipe Cactus National Monument, Puerto Blanco Mountains, ca. 18 km S of Pinkley Peak and 1 km NW of Red Tanks Wall, 18 Oct 1987, *Baker et al. 7568* (ASU); Pima County, Organ Pipe Cactus National Monument, Pinkley Peak, 28 Jan 1986, *Lechner s.n.* (ARIZ).

#### 3. Solanum homalospermum

Taxon classificationPlantaeSolanalesSolanaceae

Chiarini, Brittonia 56: 284. 2004.

Figures 2B , 9

##### Type.

Argentina. Córdoba: Dpto. Sobremonte, a 6 km de San Francisco de Chañar, a orilla de ruta rumbo a Lucio V. Mansilla, 29°46'34"S, 63°59'59"W, 29 Nov 2001, *F. Chiarini, G. Barboza & M. Matesevach 505* (holotype: CORD [CORD00004226]; isotype: NY [NY00804056]).

**Figure 9.**
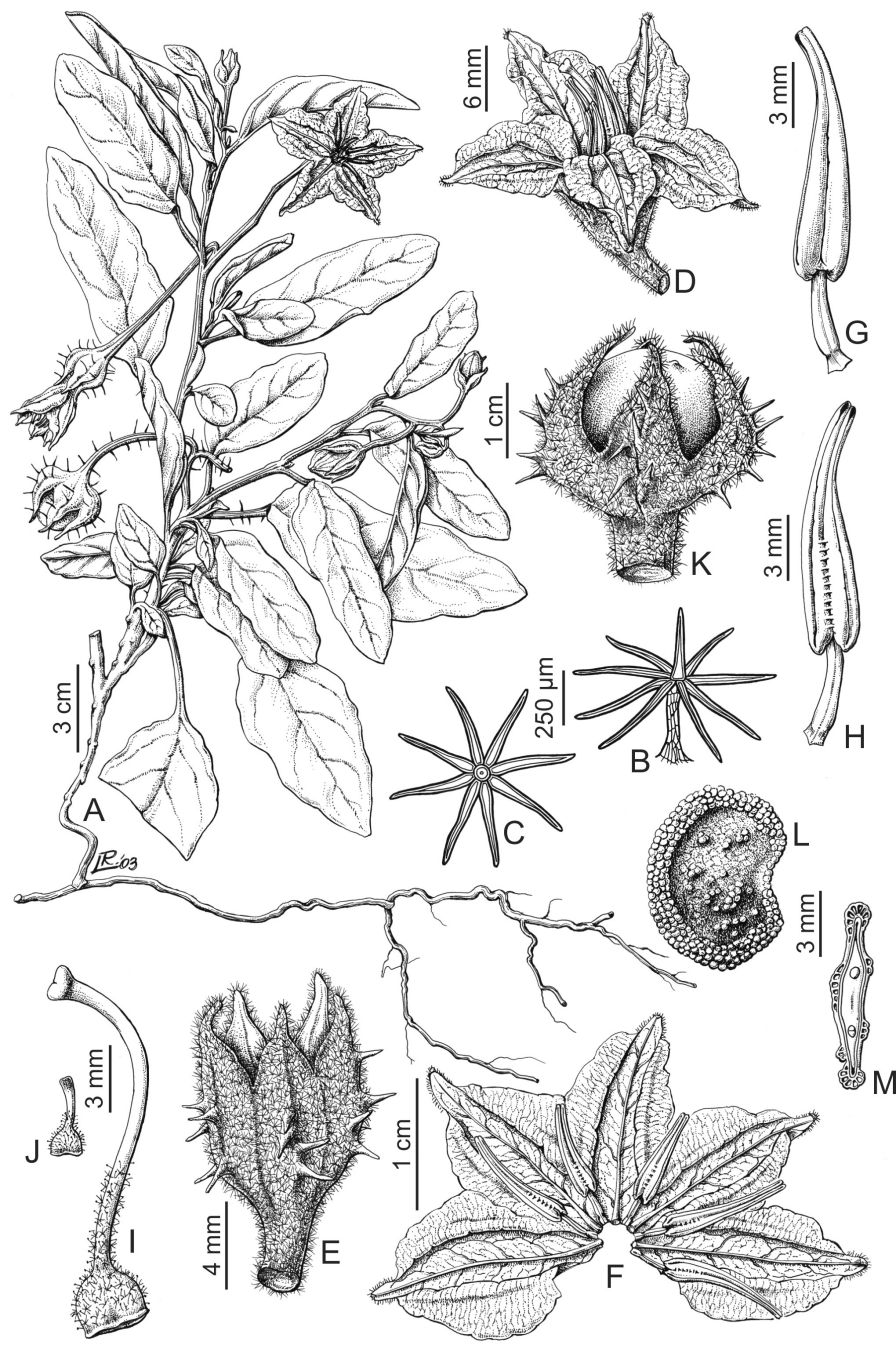
*Solanum homalospermum* Chiarini **A** Whole plant **B** Stellate trichome from adaxial leaf surface, seen from the side **C** Stellate trichome from adaxial leaf surface, seen from above, D. Long-styled flower **E** Calyx **F** Spread corolla **G** Dorsal view of stamen **H** Ventral view of stamen **I** Gynoecium of long-styled flower **J** Gynoecium of short-styled flower, K. Fruit **L** Seed **M** Transverse section of seed. Drawn by Laura Ribulgo. All from *Chiarini et al. 505* (CORD). Reproduced with permission from Flora Argentina (Chiarini 2013).

##### Description.

Erect rhizomatous shrub, 0.3–0.5 m tall. Stems sparsely armed or more commonly unarmed; young stems densely pubescent, the trichomes stellate, porrect, yellowish golden, sessile and short-stalked, the rays 6–8, ca. 1 mm long, the midpoints up to 1 mm long, equal in length to the rays; prickles if present 5–6 mm long, straight, slightly wider at the base, pale yellowish brown; bark of older stems smooth, brown or yellowish brown from persistent pubescence. Sympodial units difoliate or trifoliate, not markedly geminate. Leaves simple, 3–8 cm long, 1–3.1 cm wide, elliptic to narrowly elliptic, 4–5 times longer than wide, mostly concolorous, drying yellowish green to green; adaxial surfaces densely pubescent with translucent, mostly sessile porrect, stellate trichomes, the rays 8–14, 0.05–0.2(0.3) mm long, straight, the midpoint up to 0.1 mm, often reduced; abaxial surfaces densely pubescent with stalked porrect, stellate trichomes, the stalks to 0.5 mm long, the rays 8–10, 0.4–0.6 mm long, the midpoint shorter; principal veins 4–8 pairs, impressed adaxially, flat abaxially, spreading at ca. 45° to the midvein, the tertiary venation mostly not visible due to the dense pubescence; base acute; margins entire, rarely very shallowly lobed, the lobes 2–3 on each side, the sinus extending only 1/8 or less of the distance to the midvein, rounded; apex obtuse, rarely rounded; petiole 0.5–1.5 cm long, densely stellate-pubescent like the young stem, prickly, the prickles ca. 2 mm long, acicular. Inflorescences terminal or lateral, to 5 cm long, with up to 10 flowers, unbranched; peduncle less than 0.2 cm long, the lowest flower arising from very near the main axis; pedicels ca. 2 cm long, ca. 2 mm in diameter, robust, articulated near the base, densely stellate-pubescent like the leaf blades, the basal flower with the pedicel prickly, the prickles 2–3 mm long, orange-yellow; pedicel scars prominent, spaced 0.5–1.5 cm apart, space between the basal flower and the next much longer than that between the more distal staminate flowers. Buds ellipsoid, the calyx ca. 1/2 of the corolla length prior to anthesis. Flowers strongly heteromorphic and the plants andromonoecious, only the basal flower perfect and long-styled, the rest functionally staminate and short-styled. Calyx conical or cup-shaped, the tube 3–5 mm long, the lobes (3)6–8 mm long, 3–4 mm wide at base, lanceolate or long-deltate with acuminate apices, densely stellate-pubescent abaxially like the leaf blades, prickly in basal hermaphroditic flowers. Corolla 3–4 cm in diameter, white, drying yellowish-light brown, stellate, lobed for ca. 1/2 of the length, the lobes 0.8–1.5 cm long, ca. 1.2 cm wide, broadly deltate, densely stellate-pubescent abaxially. Stamens unequal, with the 2 adaxial anthers shorter than the 3 abaxial anthers; filament tube 1–1.5 mm, free portion of the filaments 3–5 mm; anthers 8–12 mm long, free, slightly unequal, poricidal at the tips, the pores about the same diameter as the anther apices, clearly delineated, the anther surface smooth to finely papillose. Ovary globose, minutely puberulent with simple glandular trichomes and some stellate trichomes; style of hermaphroditic flowers ca. 10 mm long, glabrous or minutely puberulent in the lower half; stigma capitate, papillose. Fruit a globose berry, 1 per infructescence, 1.2–3.5 cm in diameter when dry, the pericarp thin, smooth, glabrous, greyish brown and chartaceous when mature, when immature light green with dark stripes or marbled pattern, drying dark brown; fruiting pedicels 3–4 cm long, ca. 2 mm in diameter at base, 3–4 mm at apex, woody, strongly deflexed, sparsely armed with straight yellowish red prickles or unarmed, channelled in dry specimens; fruiting calyx accrescent, to 1.5 cm long, the lobes covering 2/3 or the entirety of the mature fruit, sparsely prickly. Seeds ca 10–30 per berry, 5–6 mm long, ca. 2.5 mm wide, flattened, reniform-rounded, brown, the surface minutely pitted and somewhat warty. Chromosome number: n=ca. 24 (Chiarini et al. in press; *Chiarini 505*).

##### Distribution

(Figure 8). *Solanum homalospermum* is endemic to north central Argentina in the Provinces of Córdoba and adjacent Catamarca at ca. 700 m elevation.

##### Ecology and habitat.

*Solanum homalospermum* grows in open areas in the dry Chaco forest region; the vegetation at the type locality is a highly disturbed palm (*Trithrinax* Mart., Arecaceae) woodland-grassland (Zak and Cabido 2002). This species has a complex root system that facilitates vegetative reproduction and spread like other members of the Elaeagnifolium clade.

##### Common names and uses.

None known.

##### Preliminary conservation status

(IUCN 2016). Endangered/Critically Endangered (EN/CR). EOO = 273 km^2^ (EN- Endangered); AOO = 12 km^2^ (EN – Endangered). *Solanum homalospermum* might best be considered CR (Critically Endangered) because the EOO and AOO measures are at the low end of the EN scale (IUCN 2016). It is known only known from two localities (see Specimens examined) and despite repeated recent searches by F. Chiarini and colleagues, has not been found since its first description.

##### Discussion.

*Solanum homalospermum* is partially sympatric with *S. elaeagnifolium* and *S. mortonii*. It is similar to *S. elaeagnifolium* in its narrowly elliptic or lanceolate leaves, but can be distinguished from that species by its porrect-stellate, rather than lepidote, pubescence and strongly andromonoecious breeding system with a single hermaphroditic flower at the base of the inflorescence. It differs from *S. mortonii* in its lanceolate, concolorous leaves, porrect-stellate rather than multangulate pubescence, and in its strongly deflexed fruiting pedicel. *Solanum homalospermum* occurs at (usually) lower elevations and to the east of the main distribution of *S. mortonii* (see Figs 8 and 12).

##### Specimens examined.

**ARGENTINA**. **Catamarca**: La Paz, km 981, 4 Apr 1950, *Brizuela 1164[a*] (CORD). **Córdoba**: Sobremonte, Sierra del Norte, ca. 6-7 km al oeste de la plaza de San Francisco de Chañar, rumbo a Lucio V. Mansilla, 20 Jan 1987, *Hunziker* & *Subils 24942* (CORD).

#### 4. Solanum houstonii

Taxon classificationPlantaeSolanalesSolanaceae

Martyn, Gard. Dict. (Miller), ed. 9, no. 91. 1807, as ‘ houstoni’

**Figure 10.**
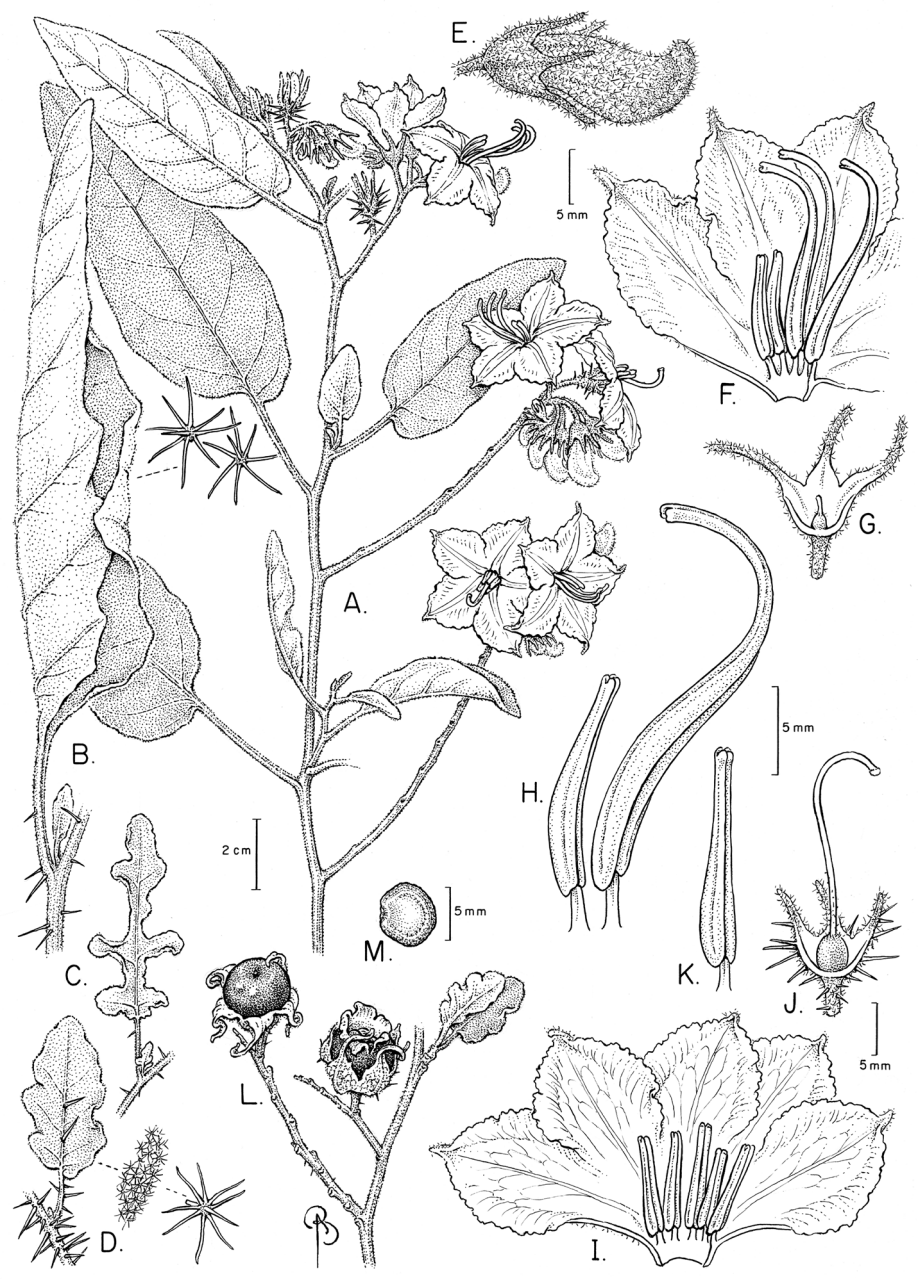
*Solanum houstonii* Martyn. **A** Whole plant **B** Leaf with prickly petiole and stellate trichomes **C** Lobed leaf **D** Prickly stem with stellate trichomes **E** Bud **F** Staminate flower **G** Ovary and style of staminate flower **H** Anthers of staminate flower **I** Hermaphroditic flower spread **J** Gynoecium of hermaphroditic flower **K** Anther of hermaphroditic flower **L** Fruits **M** Seed. Drawn by Bobbi Angell **A, E** from *Fryxell 3706* (NY00751643) **B** from *Durán et al. 4737* (NY00751647) **C** from *Reina G. 98-2082* (NY00751617) **D** from *Palmer 1243* (NY00751622) **F–H** from *Reina G. 98-2123* (NY00751616) **I–K** from *Cabrera 10755* (NY00751572) **L** from *Cabrera 11000* (NY00751567).

Figures 2D, E , 3E, F , 4B , 10

Solanum carolinense Mill., Gard. Dict. ed. 8, no. 21. 1768, as ‘carolinense’’, non S. carolinense L. (1753). Type. Mexico. Sin. loc. [probably the Yucatán peninsula or Veracruz], *W. Houstoun s.n.* (lectotype, designated here: BM [BM000514926]).
Solanum tridynamum Dunal, Encycl. [J. Lamarck & al.] Suppl. 3: 776. 1814. Type. Mexico. Sin. loc. (no specimens cited; lectotype, designated here: Service du Patrimoine Historique de l’Université de Montpellier Node-Véran, Sol. Tab. 75, MPU310713).
Solanum amazonium Ker Gawl., Bot. Reg.1: tab. 71. 1815. Type. Cultivated in London at Chelsea Physic Garden, plants received from A.B. Lambert, said to be from Mexico (no specimens cited; lectotype, designated here: Ker, Botanical Register 1, tab. 71).
Nycterium amazonium (Ker Gawl.) Sims, Bot. Mag. 43: tab. 1801. 1816. Type. Based on Solanum amazonium Ker Gawl.
Solanum herbertianum Paxton, Paxton’s Mag. Bot. 5: 269. 1838. Type. Cultivated in England at the Epsom Nursery of unknown origin, no specimens cited; lectotype, designated here: Paxton, Paxton’s Magazine of Botany, un-numbered plate labelled “Solanum herbertiana”, facing page 269).
Solanum obtusilobum M.Martens & Galeotti, Bull. Acad. Roy. Sci. Bruxelles 12(1): 152. 1845. Type. Mexico. Puebla: Tehuacán [de las Granadas], 6000 ft, Aug 1840, *H. Galeotti 1168* (lectotype, designated here: BR [BR00000523923]; isolectotypes: BR [BR00000523971], K [K000532440], K [K000063990 “Cordillera, Oaxaca”], P [P00366959 “Cordillera, Oaxaca”], W [W0003056 “Cordillera, Oaxaca”]).
Solanum tridynamum Dunal var. anoplocladum Dunal, Prodr. [A. P. de Candolle] 13(1): 334. 1852. Type. Cultivated in Geneva, Aug 1848, *Anon. s.n.* (holotype: G-DC [G00131315]).
Solanum tridynamum Dunal var. stylosum Dunal, Prodr. [A. P. de Candolle] 13(1): 334. 1852. Type. Mexico. “*J. Pavón s.n.*” [almost certainly collected by M. Sessé & J. Mociño, see Knapp 2008b] (holotype: G [G00343357]; probable isotypes: M. Sessé & J. Moçiño 1538 [MA-604686]; M. Sessé & J. Moçiño 5367 [MA-604687], 1538 bis [MA-604685]).
Solanum azureum Fernald, Proc. Amer. Acad. 35: 570. 1900. Type. Mexico. Sinaloa: Topalobampo [Topolobampo], 15–25 Sep 1897, *E. Palmer 178* (lectotype, designated here: A [A00077437]; isotypes: ARIZ [ARIZ-BOT-0005048], GH [GH00077436], MICH [MICH1109885], P [P00324679], RSA [RSA0006300], S [S09-39899, S-G-5690], UC [UC00770350], US [US-315500]).

##### Type.

Based on *Solanum carolinense* Mill.

**Figure 11.**
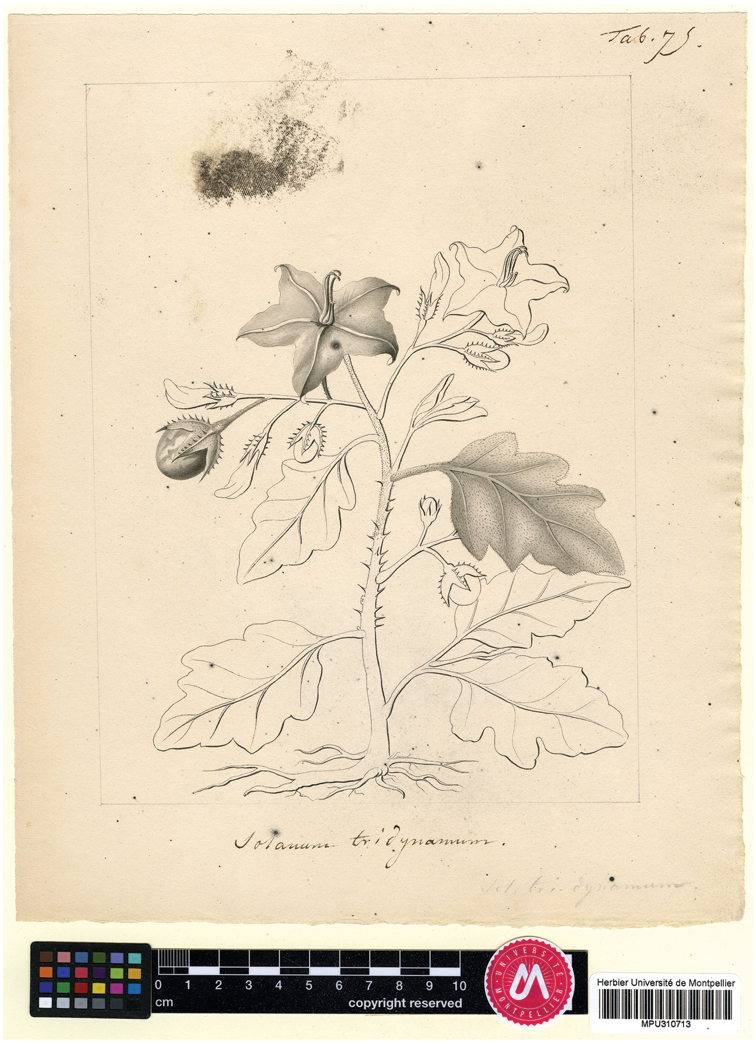
Lectotype of *S. tridynamum* Dunal. Unpublished drawing by T.F. Node-Véran (1773–1852) based on an illustration of Sessé and Mociño (MPU310713). Reproduced with permission of the Université de Montpellier – Herbier MPU (Service de Patrimoine Historique); copyright Université de Montpellier – Herbier MPU (SPH).

##### Description.

Shrub, 0.2–1.5 m tall. Stems erect, sparsely to densely armed, or unarmed; young stems densely stellate-pubescent; trichomes porrect, translucent and often reddish gold, sessile to subsessile, the rays 6 to 9, 0.1–0.3 mm long, the midpoints ca. 0.1 mm long, the prickles irregularly distributed throughout the plant, sometimes more dense on the calyx and pedicels, 1–10 mm long, 0.5–1.5 mm wide at base, straight, brown or reddish, sometimes yellow on the young stems, spaced 0.1–1 cm apart if dense, 1–10 cm apart if sparse; bark of older stems brown, glabrescent. Sympodial units usually difoliate, but not markedly geminate, sometimes plurifoliate. Leaves simple, variously lobed, 3–7(10) cm long, 1.5–3(5) cm wide, variable in shape, ovate to elliptic to broadly elliptic, 1.5–3 times longer than wide, mostly concolorous, drying yellowish green to green, densely stellate-pubescent adaxially and abaxially, the trichomes porrect, translucent, sessile to subsessile, the stalk up to 0.1 mm long, the rays 7 to 9, (0.1) 0.2–0.4 mm long, the midpoints 0.1–0.2 mm long; principal veins 3–6 pairs, raised abaxially, flat adaxially, spreading at ca. 45° to the midvein, the tertiary venation mostly not visible to the naked eye; base obtuse, rounded or truncate, sometimes oblique; margins variously lobed, rarely entire, the lobes 2–5 on each side, 0.1–1.5 cm long, usually rounded, rarely obtuse, the sinuses extending up to 1/3–1/2(2/3) of the distance to the midvein; apex rounded to acute; petiole 1–4 cm long, 1/2–1/10 of the leaf length, densely stellate-pubescent like the young stems. Inflorescence terminal or lateral, 4–7(15) cm long, usually unbranched but occasionally forked, with 4–9(26) flowers, usually with one hermaphrodite flower at the base, all distal flowers staminate; peduncle 0.5–2 cm long; rachis 1–6(9) cm long; pedicels 0.4–1 cm long, ca. 1 mm in diameter, filiform or apically dilated, densely or sparsely armed only in hermaphroditic flowers, unarmed in staminate flowers, articulated near the base; pedicel scars irregularly spaced 2–15 mm apart, prominent and brown. Buds strongly curved and zygomorphic, more so in staminate flowers; the corolla exserted ca. halfway from the calyx tube before anthesis. Flowers 5-merous, heterostylous, heterandrous and markedly dimorphic, the plants strongly andromonoecious, the basal flower long-styled and hermaphroditic, the distal flowers short-styled and staminate. Calyx tube 0.8–2 cm long, cup-shaped, the lobes 0.7–2.5 cm long, 1–2 mm wide, long-triangular with an elongate acumen, unarmed in staminate flowers, densely armed in hermaphroditic flowers, the prickles 0.5–1 cm long, 0.5–1 mm wide at base, yellow or reddish, straight, spaced 0.5–2 mm apart. Corolla 2–4 cm in diameter (slightly larger in staminate flowers), lilac or purple or occasionally white, the midvein paler and greenish yellow, drying yellow or brownish tan in herbarium specimens, stellate to broadly stellate, lobed ca. halfway to the base, the lobes 1–1.5 cm long, 0.8–1 cm wide, spreading or slightly reflexed at anthesis with abundant interpetalar tissue, the abaxial surfaces densely pubescent along the middle portions where exposed in bud, the adaxial surfaces glabrous or with a few trichomes along the margins, the tips somewhat cucullate. Stamens strongly unequal in both hermaphroditic and staminate flowers, with the 2 adaxial anthers shorter than the 3 abaxial anthers; filament tube minute; free portion of the filaments 1.5–2 mm long, glabrous; anthers tapering, yellow or occasionally purple (Sinaloa and Sonora), the surfaces smooth, poricidal at the tips, the pores about the same diameter as the anther apices, clearly delineated, directed distally, in hermaphroditic flowers three longer abaxial anthers ca. 9 mm long, two shorter adaxial ca. 6 mm long, in staminate flowers three curved abaxial anthers 15–20 mm long, two straight adaxial anthers ca. 10 mm long. Ovary in hermaphroditic flowers ca. 2 mm in diameter, conical. porrect-stellate pubescent in the distal third, the trichomes sessile to subsessile, with 7–10 rays, the rays 0.1–0.2 mm long, the midpoints 0.1–0.15 mm long; ovary in staminate flowers vestigal; style 20–30 mm long in long-styled flowers, in staminate flowers ca. 1.5 mm long; stigma capitate and slightly bilobed in hermaphroditic flowers, indistinctly bilobed and much smaller in staminate flowers. Fruit a globose dehiscent berry, (1-)2–2.5 cm in diameter, pale green with darker green mottled areas when young, dark brown or orange-brownish when dry, dehiscing irregularly in several parts, usually breaking open irregularly in the upper half of the fruit, the pericarp thin, smooth, glabrous; fruiting pedicels 0.5–1.5 long, 2–3 mm in diameter at base, 2–5 mm in diameter at apex, erect, herbaceous to woody; fruiting calyx (hermaphroditic flowers only) strongly accrescent, the lobes covering up to the total length of the fruit, sparsely or densely armed with prickles up to 8 mm long. Seeds ca. 20–40 per berry, 3–4 mm long, 3–4 mm wide, flattened reniform, dark brown to black, the surface minutely pitted, the testal cells pentagonal. Chromosome number: n=12 (Averett and Powell 1972; Chiarini et al. in press).

##### Distribution

**Figure 12.**
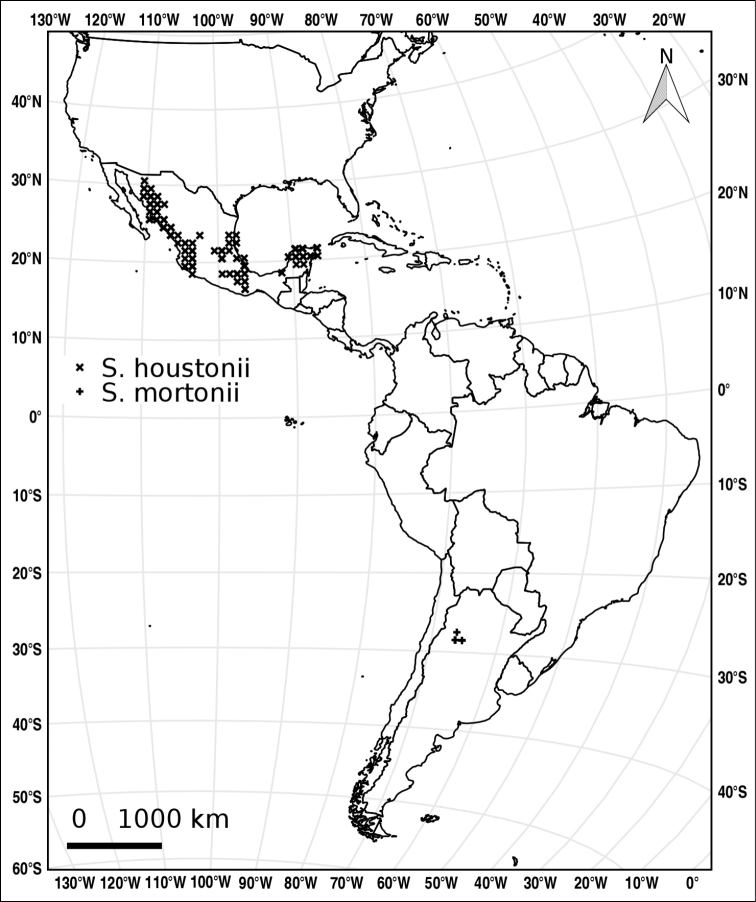
Distribution of *S. houstonii* and *S. mortonii*.

(Figure 12). *Solanum houstonii* is endemic to Mexico, but widespread in south central Mexico on both coasts, from the Yucatán Peninsula and Veracruz to Sinaloa and Sonora where it is sympatric with *S. hindsianum*; from sea level to nearly 2000 m.

##### Ecology and habitat.

*Solanum houstonii* grows in a wide variety of dry and semi-deciduous forests, from Sinaloan thorn scrub to the humid semi-deciduous forests of the Caribbean coast (selva mediana subcaducifolia) and in open situations such as coastal dunes. Like other members of the group, it can form large colonies, especially in disturbed ground.

##### Common names and uses.

Mexico. Colima: mala mujer; Puebla: diente de burro; Sinaloa: melón de coyote, coyotillo; Sonora: sacamanteca; Veracruz: berenjena espinosa, necachane; Yucatán: berenjena de monte, k’omya’ axnik (Maya).

The leaves of *S. houstonii* are used for cleansing after birth and as a dieting aid (*Nee & Taylor 29629*) and for skin diseases and problems (*Rivera 90*).

##### Preliminary conservation status

(IUCN 2016). LC (Least Concern). EOO = 1,837,020 km^2^ (LC – Least Concern); AOO = 1,052 km^2^ (VU- Vulnerable). Based on its wide distribution *S. houstonii* is not of immediate conservation concern, but populations across Mexico exhibit significant heterogeneity in morphology that is presumably also reflected in genetic variability.

##### Discussion.

*Solanum houstonii* has the most pronounced flower dimorphism in group, with differences in size and shape of both corolla and anthers. Staminate flowers are larger and exhibit stronger anther zygomorphy, while hermaphrodites tend to be smaller and with anthers of more equal size. The anther dimorphism in hermaphroditic flowers of *S. houstonii* is similar to that seen in both flower morphs of *S. hindsianum* and *S. homalospermum*. On the coast of western Mexico *S. houstonii* grows in sympatry with *S. hindsianum*; it differs from that species in its larger fruit, more dimorphic flowers, more prickly calyx of long-styled flowers, and usually lobed leaves. Populations from around the bay of Topolobambo in Sinaloa are unusual in having deeply pinnatifid leaves, and individuals from northwestern Mexico are polymorphic for purple or yellow anther coloration.

This species was long known as either *S. amazonium* or *S. tridynamum*; Nee (1999) found that the name coined by Thomas Martyn (Miller 1807) as a replacement for the illegitimate *S. carolinense* Mill. had priority, thus *S. houstonii* becomes the correct name for this distinctive and relatively widespread Mexican species. Martyn coined his new name for this plant in honour of its collector, William Houstoun (with the original spelling ‘*houstoni*’), who sent specimens and seeds of plants collected in western Mexico to Philip Miller at the Chelsea Physic Garden in London (Stearn 1974); many of Houstoun’s plants were grown in the gardens and glasshouses of Chelsea. We select here as the lectotype the single specimen in BM (BM000514926) with labels in Houstoun’s hand and that bears another in Miller’s hand (the upper label); both of these feature verbatim in the polynomial description of *S. carolinense* Mill. (see image in Global Plants, http://plants.jstor.org/ - search for *S. houstonii*).

The protologue of *S. tridynamum* (Dunal 1814) cites no specimens, only two illustrations. One was an illustration in the collection of original drawings done for the Sessé and Mociño expedition to Mexico and Central America (see McVaugh 2000) and the other Dunal’s own, unpublished illustration copied from this original, now held in the collections at MPU. Dunal is likely to have seen the former illustration when Mociño was in Montpellier between 1812 and 1817 (see McVaugh 2000: 12); this set of original illustrations was then copied for Candolle, but no images of *Solanum* species are held in the set of copies at G. Two illustrations in the Torner Collection of the Hunt Botanical Institute (accession numbers 6331.0674 and 6331.1883) both clearly show the distinctive flower morphology of *S. houstonii*. One is a sketch (6331.1883) upon which the other (6331.0674) showing the whole plant is based. The illustration made by Node-Veran and cited by Dunal in the protologue (MPU310713; Fig. 11) is apparently copied from these originals, but we select it as the lectotype of *S. tridynanum* because it is the only one of these elements we can be sure was seen by Dunal at the time he described the species.

*Solanum amazonium* was described from specimens cultivated at the Chelsea Physic Garden sent by Aylmer Lambert (Ker 1815); two sheets at Vienna (W-292940, W-292941) from the A.B. Lambert herbarium are possibly from the same introduction, but are not type material. The plate accompanying the description was prepared from live plants cultivated at Chelsea and is the only unambiguous authentic material that could be used as a lectotype. The protologue contains an excellent description of the anther dimorphism that differs in hermaphroditic and staminate flowers (Ker 1815). *Solanum herbertianum* was similarly described from cultivated material (Paxton 1838) of unknown origin that was grown in the Epsom Nursery in southern England. The plate is the only unambiguous material associated with the protologue and we select this here as the lectotype. The plant depicted is completely devoid of prickles (like that in Ker 1815) and may have come from the same stock. In contemporaneous horticultural collections in W there appear to have been two distinct morphs of *S. houstonii* in cultivation in Vienna, one with (e.g., W-0001800) and the other without prickles (e.g., W-0001799).

The lectotype we have selected for *S. obtusilobum* is one of two sheets held in BR, where Martens and Galeotti worked. We have selected the sheet (BR00000523923) that was labelled (but not published) as lectotype by M. Nee in 1986. The other duplicate of *Galeotti 1168* in BR (BR00000523971) is clearly labelled as “2^ème^ exemplaire” and is less complete. The protologue (Martens and Galeotti 1845) cites *Galeotti 1168* from “montagnes cactifères et arides près Tehuacan de las Granadas” in today’s state of Puebla. The sheet selected as the lectotype (BR00000523923) states only “Tehuacan”; other sheets labelled with the same number (*Galeotti 1168*) but with the locality “Cordillera de Oaxaca” are recognised here as isolectotypes, they are morphologically extremely similar to the sheets in BR.

Of the two varieties described in Dunal (1852) var. anoplocladum was based on plants cultivated in the gardens in Geneva and a specimen in the Candolle herbarium was cited, and var. stylosum was based on material from “Herb. Pavon” and is likely to be a collection made by Sessé and Mociño in Mexico (see Knapp 2008b for a discussion of the distribution of Sessé and Mociño’s collections by Pavón). This latter specimen (G00343357) could be related to the illustration used in the description of *S. tridynamum*, but does not exactly match it; it is probably from western populations with more deeply lobed leaves.

Of the two sheets of *Palmer 178* held in the Harvard University herbaria (where Fernald worked) we select the A sheet (A00077437) as the lectotype of *Solanum azureum* because it is better preserved and has flowers. This plant comes from populations on the west coast of Mexico with deeply sinuate leaves and flowers with occasionally purple anthers.

##### Selected specimens examined.

**MEXICO. Campeche**: 3 km al S de Bolochen de Rejón, cerca de las Grutas de Xtacumbilxunan, por la carretera via ruinas a Campeche, 25 Jul 1986, *Cabrera & de Cabrera 11770* (MEXU); Calkiní, 8 km al E de Tancuche, sobre el camino Calkini-Punta Arenas, 26 Jul 1987, *Cabrera & de Cabrera 13920* (MEXU); 8 km al O de Nunkini, sobre el camino a Calkini-Punta Arenas, 19 Sep 1987, *Cabrera & de Cabrera 14318* (MEXU); Ciudad del Carmen, Isla del Carmen, 12 km al SO de Puente de la Unidad, sobre la carretera Campeche-Ciudad del Carmen, 21 Sep 1987, *Cabrera & de Cabrera 14535* (MEXU). **Chihuahua**: Urique, on road coming out of canyon from Urizue, 17 Aug 2005, *Ford 11* (NY); Batopilas, La Bufa, SE of Creel, 12 Sep 1957, *Knobloch 419* (BM); SW part of state, on road ca. 16.5 mi W of Ocampo, 1 Sep 1989, *Mayfield et al. 301* (MEXU); SW Chihuahua, Aug 1885, *Palmer 237* (BM, K, MEXU, NY). **Colima**: Coquimatlán, Coquimatlán, en un terreno solo cercano a zona habitacional, 1 May 2013, *Gaitón Hinojosa et al. 2013- 05* (MEXU); 400 m al norte de El Trapiche, 3 Sep 1992, *García Torres 78* (MEXU); Colima, 1 km antes de Cardona, 15 Mar 1994, *García Torres 148* (MEXU); Armería, Rancho El Mirador, Valle de A, 19 Aug 1994, *García Torres* & *Navarrete 183* (MEXU); Coquimatlán, Rancho Buenos Aires, 1 km del cruce Coquim.-El Poblado, 25 Jul 1995, *García Torres 315* (MEXU); Colima, 2 km al SE de Colima, 20 Dec 1040, *Langman 3190* (MEXU); Coquimatlán, Pueblo Juárez, 14 Jan 1985, *Maillet 14* (MEXU); Comala, Ejido La Caja, cerca del Río Armería, 4 Aug 1992, *Navarrete de la Paz 33* (MEXU); Colima, Jul 1897, *Palmer 95* (LE, S); Manzanillo, 1 Dec 1890, *Palmer 1035* (K, NY); Comala, Predio Cerro Alto y Potrero Nuevo 3 km al suroeste de Los Colomos, 28 Aug 1990, *Román Miranda 1371* (MEXU); Armería, 1 km de la desv. carret. Colima-Manz, rumbo a Armería, 26 Feb 1994, *Román Miranda 1795* (MEXU); Comala, 8 km al N de Comala, brecha a La Caja, 22 Aug 1984, *Santana M.* & *Cervantes A. 546* (MEXU). **Durango**: San Dimas, La Desmontada, 7 km al S, por el camino a Mala Noche, 7 Mar 1990, *González 2394* (MEXU); Tabahuelo (al catorce) 196 km al W de Tepehuanes, 31 Aug 1983, *Torres C. et al. 3561* (MEXU, NY). **Jalisco**: Guadalajara, 5 miles S of Guadalajara, 12 Aug 1947, *Barkley et al. 7505* (MEXU); Tecolotlán, 1.5 km al NE de Lindavista, km 59 carretera Guadalajara-Barra de Navidad, 10 Jul 2003, *Carrillo-Reyes* & *Ortíz-Catedral 4139* (MEXU); Huejuquilla, Rancho Los Arroyos del Agua, 15 km al NW de Huejuquilla, 4 Aug 1990, *Flores M. 2037* (MEXU); Villa Guerrero, 41 km al SW de Villa Guerrero, camino a Chimaltitlán, 18 Oct 1983, *Lott et al. 2016* (BH, MEXU, NY); San Martín de Bolaños, 14 km al NE de San Martín de Bolaños, 1 km al SE de Chimaltitán, 21 Oct 1983, *Lott et al. 2114* (MEXU); Amacueca, 2.5 km al NE de Cofradía del Rosario, 7 Nov 1992, *Novoa L.* & *Acevedo R. 54 A* (MEXU); San Martín de Bolaños, 5 km salida oeste a al Zualaga, 17 Feb 1992, *Reynoso Dueñas et al. 634* (MEXU); Atoyac, camino a Atoyac, a partir de la carretera libre a Zacoalco-Sayula, 27 Jul 1991, *Rodríguez C. 2107* (MEXU); Zapotitlán de Vadillo, 5 km al N de Zapotitlán de Vadillo, 26 Oct 1994, *Sánchez* & *Santana M. 46* (MEXU); El Limón, Rancho El Recodo, 2 km al E de San Miguel Hidalgo, 1 Sep 1987, *Santana Michel 2955* (MEXU); Ciudad Guzmán, Floripondio, km 13 carretera Autlán, 12 Mar 1994, *Villareal de Puga et al. 16135* (MEXU); Tenamaxtlán, inmediaciones de la presa, camino a Tecolotlán y Tenamaxtlán, 24 Feb 1980, *Villarreal de Puga 11507* (MEXU); Atoyac, Laguna de Sayula, entre el km 51-52 Autopista Guadalajara-Colima, 27 Mar 1993, *Villegas F.* & *Macías R. 219* (MEXU); Amacueca, Laguna de Sayula, carretera Guadalajara-Sayula, 3 km después de la desviación a Tapalpa, 9 Jul 1993, *Villegas F.* & *Ramírez D. 329* (MEXU); Autlán, ca. 1 mile south, 21 Aug 1949, *Wilbur* & *Wilbur 2469* (DS, DUKE, MEXU). **Nayarit**: Nayar, recorrido entre el poblado y Aguamilpa y el Cerro del Colomo, 20 Sep 1991, *Benítez-Paredes 3405* (MEXU); Nayar, 15 km al SW de Jesus Maria, camino a la Mesa del Nayar, 28 Jul 1990, *Flores F. et al. 2122* (MEXU); Nayar, Santa Cruz, camino rumbo al rancho El Ocotillo [Santa Cruz de Guaybel], 11 Sep 1990, *Rodríguez González 143* (MEXU); Tepic, km 20-40 terraceria hacia La Presa de Aguamilpa, 15 Jan 1988, *Téllez V. 11198* (MEXU). **Oaxaca**: Tepelmeme Villa de Morelos, 45 miles S of Tehuacan, 26 Aug 1967, *Clarke et al. s.n.* (CAS); W slopes of Sierra Zongolica, 2km (by road) above Teotitlán del Camino on road to Huautla de Jiménez, , 8 Jul 1978, *Cochrane et al. 8516* (BH, BM, CAS); along highway 190, 55 miles SE of Oaxaca, 2 Jul 1977, *Croat 39945* (MEXU); carretera Oaxaca-Mitla km 39, 10 Feb 1989, *Escalante Membrillo s.n.* (MEXU); San Pedro Totolapa, Dto. Tlacolula, Valles Centrales, 3.5 km de Totolapan, hacia Tehuantepec, 14 Jul 1988, *Flores Martínez 1328* (MEXU, NY); 2 km E of Tomallín, 18 Oct 1992, *Newman et al. 435* (K); Mejia Station, 9 May 1894, *Pringle 5850* (MEXU, MEXU, VT); Tlacolula, a 1 km al E de San Juan Guegoyache, o a 5 km al NE de Totolapán, 22 May 1982, *Rico Arce et al. 378* (BM, MEXU); Valerio Trujano, Tomellín, Sep 1905, *Rose* & *Painter 10073* (K, NY); beyond reservorio 2 km E of San Juan de los Cues, 6 Dec 1994, *Way WS-023* (K). **Puebla**: Cerro Tres Cruces, 10 Dec 1967, *Boege 689* (DUKE); Acatlán de Osorio, Route 190 near km 293, ca. 8 km SE of Acatlán, 12 Aug 1966, *Cruden 1174* (MEXU); Coxcatlán, 1 km SE of Guadelupe Victoria, path to San José Tilapo, 13 Sep 2004, *Guzmán C. 2121* (K); Acatlán de Osorio, Acatlán, 26 Jul 1942, *Miranda 2119* (MEXU); Zapotitlán, Zapotitlán de las Salinas, 4 km al NE de Zapotitlán de las Salinas, sobre la Carretera a Tabuacán, 2 Jun 1975, *Rzedowski 33231* (CAS, CORD); along Highway 190, 41.5 miles SE from Tehuitzingo, 2 Aug 1975, *Torke et al. 381* (MEXU); Zapotitlán, 11 km SW of Tehuacan, on Hwy 125, 30 Jul 1974, *Whalen 16* (BH). **Querétaro**: Cadereyta, entre Nizarrón [Vizarrón de Montes] y Higuerillas, 23 Aug 1905, *Altamirano 1678* (MEXU); Cadereyta, Vizarrón, ca. 10 km al N, 22 Jul 1991, *Carranza 3292* (CAS); km 110 hacia al poblado de Camargo, carretera federal 120, 7 Jul 2000, *Lira Charco et al. 1379* (MEXU); Peñamiller, Nacimiento, ca. 1.5 km de La Higuera rumbo al Balneario El Oasis, 12 Jun 2000, *Ocampo et al. 874* (MEXU); Peñamiller, Mex. 120 entre Camargo y Peña Blanca, 28 Sep 2016, *Ochoterena et al. 976* (MEXU); Tolimán, carretera San Pablo-Higuerillas, 17 Jun 1992, *Orozco H. et al. 9957* (MEXU); Cadereyta, cerros calizos al este de Vizarrón, 31 Aug 1994, *Orozco H. et al. 10757* (MEXU); Pinal de Amoles, orilla del Río Estorax, cerca de Bucarali, 15 Apr 1988, *Rzedowski 46431* (MEXU); Peñamiller, 3.5 km al NE de Peñamiller, 25 May 1977, *Zamudio 2097* (MEXU); Tolimán, 7 km al N de San Pablo Tolimán, sobre la brecha a Higuerillas, 12 Jul 1977, *Zamudio 2212* (MEXU); Tolimán, 2 km al NE de “El Chilar”, 16 Jun 1978, *Zamudio 2868* (MEXU); Cadereyta, ca. 1 km al N de La Florida, 14 Jul 1997, *Zamudio* & *Zamudio 10249* (MEXU). **Quintana Roo**: Isla Mujeres, a 1 km al N de El Faro de la punta sur, sobre la carretera perimetral, 8 Jan 1986, *Cabrera & de Cabrera 10461* (CIQR, MEXU); Isla Mujeres, camino al Puerto de Abrigo, 1 Nov 1970, *Cabrera et al. 17207* (CIQR, MEXU); Cozumel, Cobá, Ruinas de Cobá, 24 Nov 1980, *Chan et al. 45* (CIQR, MEXU); Isla Mujeres, a small isle off the coast at Puerto Juarez, along gravel road between lighthouse and Garrafón beach on the S end of the isle, 11 Jan 1979, *Doebley* & *Sager 203* (CAS, MEXU); Cozumel, Cozumel Island, coast of Yucatan, 11 Jun 1885, *Gaumer 135* (K); José María Morelos, Chichankanab, *Gaumer 1708* (BM, CAS, K); Cozumel, Cobá, parque arqueológico y natural Cobá, entre el camino y el tipo de pinturas, 21 May 1977, *López Franco* & *Villers 1100* (MEXU); Cozumel, carretera Tulúm-Cobá, 10 km antes de llegar a Cobá, 19 Feb 1976, *Moreno 494* (MEXU); Cozumel, Cobá, Grupo Coba, 20 Aug 1981, *Rico-Gray* & *Chan 421* (MEXU); Isla Mujeres, a 6 km al SE de la cuidad de Isla Mujeres, a 200 m del Faro despues de Garrafón, 11 Feb 1980, *Téllez V.* & *Cabrera 1576* (BM, MEXU); Cobá, orilla de Laguna Cobá, 18 Oct 1980, *Téllez V. et al. 766* (MEXU); Solidaridad, Cobá, zona arqueologica, 9 Sep 1980, *Ucán Ek 394* (CICY). **San Luis Potosí**: Tampamolon, 1 km N of Tampamolon, 12 Oct 1978, *Alcorn 2028* (MEXU); Rioverde, ca. 27 km del entronque a la Presa El Realito, 19 Feb 2010, *Guzmán C. 3279* (K). **Sinaloa**: Culiacán, La Lima, ca. 1 km al NE de La Lima, 12 Feb 1984, *Aguayo C.* & *Gómez R. 2* (MEXU); Escuinapa, orilla de estero Navajas, ca. 8 km de Isla de Bosque, 5 Jan 1985, *Aguiar H. 59* (MEXU); Badiraguato, Ojito de Agua, ca.3 km de Huajote, carretera Badiraguta-Surutato, 7 Mar 1995, *Armenta et al. 132* (MEXU); Cofradía, 7 Apr 1934, *Bailey 21* (BH); Mazatlán, 7.4 km N of the Tropic of Cancer on road from Mazatlán to Culiacán, 31 Oct 1985, *Bartholomew et al. 3643* (CAS, MEXU); Salvador Alvarado, cerros al SE de Tabullal a ca. 5 km, 15 Aug 1988, *Bojórquez B. 721* (MEXU); Morcocito, Terreros, 3 miles N, ca. 48 miles N of Culiacán, 28 Jan 1962, *Breedlove 1511* (DS, DUKE); Badiraguato, 28 km E of Badiraguato, 20 Aug 1986, *Breedlove* & *Anderson 62857* (CAS); Cosalá, ca. 12 km al NO de Cosalá, 18 Oct 1986, *Carrasco et al. 151* (MEXU); Agua Caliente de Zevada, por la casa de Silveiro Perez, *Chapiro 41* (MEXU); Culiacán, km 27 carretera Culiacán-Guamuchil, Ejido La Campana, 29 Sep 1984, *Chávez Montes* & *Niebla Armenta*, *82* (MEXU); Calmoa, Río Fuerte, 21 Nov 1933, *Gentry 930* (DS); Bahia Topolobampo, Cerros de Navachiste about Bahia Topolobampo, 26 Sep 1954, *Gentry14362* (MEXU); Mazatlán, zona ganadera del poblado El Quelite, desviación km 33 carretera Mazatlán-Culiacán, 2 Nov 1994, *González E. s.n.* (MEXU); Elota, La Roble, Sind. La Cruz, *González Ortega 56* (MEXU); Mazatlán, Urias, *González Ortega 5609* (K, MEXU); Mazatlán, Mazatlán, Jul 1934, *González Ortega 7249* (CAS); Elota, Mexican Highway 15, 7 miles N of the bridge over Río Piaxtla, ca. 87 km N of Mazatlán, 16 May 1973, *Hansen et al. 1402* (BH, MEXU); Cosalá, Mineral de Nuestra Señora, 22 Jan 1988, *Hernández A.* & *Hernández V. 567* (MEXU); Guasave, carretera 15 (libre) km 81, 1 km al S de Terrero, entre Mazatlán y Guamúchil, camino de terracería hacia El Progreso, menos de 1 km al E de la carretera 15, 30 Sep 2007, *Igic* & *Vallejo-Marín 07s*-*20* (MEXU); Culiacán, La Divisa km 6 al N de la carretera Culiacán-Sanalona, 25 Feb 1984, *López Felix* & *Antio 16* (MEXU); Culiacán, 23 km al N de Culiacán camino a El Barco, 30 Jul 1983, *Martínez S. et al. 4083* (MEXU, NY); Salvador Alvarado, along Hwy 15 about 59 miles N of Culiacán, 22 Dec 1971, *Norris et al. 20104* (CAS, MEXU); Culiacán, carretera Culiacán-Guamuchil km 15, 12 Oct 1984, *Ochoa Avalos* & *Bojórquez B. 188* (MEXU); Culiacán, El Chaparral, 30 Sep 1994, *Pineda C. 477* (MEXU); Cosola, junction between Mazatlán and Culiacán, 22 Feb 1970, *Roderick et al. 3136* (CAS); Culiacán, Costa Rica, km 5 carretera Costa Rica-C. Internacional, 18 Feb 1984, *Rosas J.* & *Sánchez G. 1* (MEXU); Imala, 20 miles NE of Culiacán, about 1-2 miles beyond Cofradia along artificial lake, 24 Apr 1959, *Thomas 7683* (CAS, DS, MEXU); Culiacán, La Loma de Rodriguera, camino a Las Guasimas, ribera del Río Humaya, 4 Feb 1984, *Torres et al. 21* (MEXU); Culiacán, Dique La Primavera, ca. 8 km al SE de Culiacán, 14 Feb 1985, *Vega Aviña* & *Bojórquez B. 1467* (MEXU); Mazatlán, El Venadillo, ca. 5 km al N de Mazatlán, 5 Aug 1985, *Vega Aviña 1595* (MEXU); Ahome, San Miguel de Zapotitlán, al pie de Barobampo, 2 Mar 1999, *Vega Aviña* & *Gutierrez 9884* (MEXU); E of Playa Escondida, near Mazatlán, 20 Dec 1974, *Webster 19916* (MEXU); along Hwy 150, 20 miles N of Rosario, 27 Jul 1975, *Whalen 182* (BH); Mocorito, Pericos, 8 miles S, 17 Mar 1955, *Wiggins 13150* (DS, MEXU). **Sonora**: Alamos, 25 miles E of Navajos on road to Alamos, 19 Feb 1953, *Blakley 3-1652* (DS); Cañada Tetabejo, Sierra Libre, al S de la Cuidad de Hermosillo, por la Carr. Fed. 15, 8 Aug 1995, *Búrquez 95-154* (MEXU); Cañon el Abolio, Sierra Libre, al S de la Ciudad de Mermosillo, por la carretera Federal 15, 16 Jan 1996, *Búrquez 96-15* (MEXU); Cañón Tepoca, km 177 a los lados de la Carr. Fed. 16, 7 Sep 1996, *Búrquez et al. 96-960* (MEXU); Sahuaripa, región de Los Mulatos, Cerro La Estrella, 1 km al SE del pueblo Mulatos, 11 Sep 1996, *Búrquez* & *Yetman 96-1127* (MEXU); Baviacora, 40 km al NE de Ures, camino a Moctezuma, 22 Jul 1978, *Castellanos 206* (MEXU); Cumpas, Jécori, in ravine 1 mile N of Jécori, 20 Nov 1939, *Drouet et al. 3695* (DS); Arivechi, 2 km al NW de Arivechi, carr. a Sahuaripa, 27 Sep 1996, *Flores Martínez* & *Arvizo Y. 4751* (MEXU); Alamos, Río Mayo, San Bernardo, 10 Feb 1955, *Gentry 1294* (K, MEXU); La Tinaja, 19 Nov 1890, *Hartman 243* (K); Bacanora, 18 miles by road E of bridge of Río Yaqui at El Novillo, 27 Oct 1965, *Hastings* & *Turner 65-197* (DS); Alamos area, SE side of Cerro Prieto, W of Alamos, 29 Dec 1989, *Joyal 1339* (MEXU, NY); Las Cabras, ESE of Alamos, 13 May 1990, *Joyal 1423* (MEXU); La Palmita, between Bacadehauchi and Nacori Chico, Tedehuachi drainage into Río Bavispe, 6 Feb 1991, *Joyal 1520* (MEXU); road from Hermosillo to Sahuaripa, 3.8 mi E of Río Yaqui, 21 Feb 1987, *Landrum et al. 5409* (MEXU, NY); Onavos, Rancho la Mula, 5 km W of Agua Amarilla, 28.2 km SW of Río Yaqui on Mex. 16 (km 195 E of Hermosillo), 27 Mar 1997, *Reina G.* & *Van Devender 97-297* (CAS, MEXU); Huatabampo, ca. 6 km NW of Camahuiroa on road to Las Bocas, near Bachomojaqui, 24 Nov 1998, *Reina G. et al. 98-2123* (MEXU, NY); Madgalena, Río Madgalena at Tubutama road, 23 Jul 2001, *Reina G.* & *Van Devender 2001-598* (MEXU); Navajoa, 20 Jan 1931, *Souviron* & *Erlanson 27* (BH); Alamos, estación de microondas, Cerro Prieto, 15 km al DE de Navojoa, carretera a Alamos, 1 Oct 1983, *Tenorio L.* & *Torres C. 4644* (MEXU); San Javier, km 135 carretera 16 a 10 km al NW de al desviación al pueblo, 29 Aug 1996, *Varela E. 96-184* (MEXU); San Javier, Cerro El Halcón ladera N a 3.5 km al NE del poblado camino brecha hacia el Cañón del Aliso, 11 Jan 1997, *Varela* & *Cuamea 97-01* (MEXU); La Colorada, Colorado, 7 miles NE between Colorado and Mazatán, 6 Sep 1941, *Wiggins* & *Rollins 326* (DS, NY); Mazatán, Mazatán, 5 miles S between Mazatán and Colorado, 7 Sep 1941, *Wiggins* & *Rollins 382* (DS, NY); Cajeme, small valley in foothills 17 miles NE of Cajame on road to Tesopaco, 3 Mar 1933, *Wiggins 6393* (DS); Ures, W side of mountains 6 miles N of Ures on road to Babiacari, 20 Sep 1934, *Wiggins 7333* (DS). **Tamaulipas**: Aldama, 13 km NE of Aldama on road to Barra del Tordo, 22 Sep 1981, *Fryxell 3706* (BH, MEXU, NY); Aldama, entre Rancho Nuevo y San Vicente, 28 Jul 1970, *González-Medrano et al. 3122* (MEXU); Aldama, Ejido El Nacimiento, 5.5 km al S de Aldama, 31 Aug 1984, *Hernández* & *Romo 1174* (MEXU); Soto de la Marina, Campamento Tortugero La Pesca, 4 Apr 2007, *Martínez S. 39209* (MEXU); Aldama, 18 km E de la carretera Aldama-Barra del Tordo, 27 Jun 1992, *Mora-López 189* (MEXU); Aldama, 17.2 km al NW de la desviación a Rancho Las Yucas, 26 Jun 1983, *Torres C. 3141* (MEXU, NY); Aldama, 5 miles N or ca. 75 miles N of Tampico, 15 Jul 1984, *Wilbur 35210* (DUKE). **Veracruz**: Actopán, alrededores de Laguna de la Mancha, 28 Nov 1975, *Acosta* & *Dorantes 663* (MEXU, NY); Emiliano Zapata, 15 miles SE of Xalapa, 3 Aug 1947, *Barkley et al. 2616* (CAS); Carrizal, 0.5 km de la desviación a Carrizal, por la carretera Jalapa [Xalapa]-Veracruz, 20 Jul 1975, *Calzada 1837* (MEXU); Veracruz, Colonia Las Amapolas, en el km 100 de la carretera Xalapa Veracruz, 13 May 1977, *Calzada 3182* (MEXU); Vega de Alatorre, Cañada de Mesillas entrada por Santa Ana, 24 Jul 1981, *Calzada 7725* (MEXU); Actopán, Playa Paraíso, 0.5 km antes de la playa, 6 Sep 1977, *Castillo Campos et al. 189* (MEXU); Coatepec, Cerro de las Palmas, 2 km antes de Jalcomulco en las faldas del Cerro de las Palmas, 20 May 1979, *Castillo Campos* & *Tapia 700* (MEXU); Apazapan, 1 km E del pueblo, en las faldas (N) del Río Jalcomulco, 10 Oct 1991, *Castillo Campos* & *Birke B. 6971* (MEXU); El Remuladera, carrretera Conejos a Huatusco, 13 Jul 1970, *Chavelas P. ES- 971* (MEXU); Xalapa, km 13 carretera Jalapa[Xalapa]-Veracruz, 5 Dec 1985, *Chehaiber* & *Espejo 201* (MEXU); entre rancho del Diamante y el rancho del Capulin, camino a Misantla, 3 Nov 2001, *Cruz Durán et al. 5483* (MEXU); Actopán, La Mancha, carretera Cardel-Nautla, 1 Aug 1971, *Dorantes 251* (CAS, MEXU); Emiliano Zapata, Barranca de San Antonio al E de Corral Falso, carretera Xalapa-Veracruz, 17 Aug 1971, *Dorantes 260* (CAS, MEXU); Alto Lucero de Gutiérrez Barri, W de Laguna Salada, 26 Jun 1972, *Dorantes 1033* (CAS, MEXU); Actopán, Sierra Manuel Diaz, 11 Jul 1972, *Dorantes 1333* (CAS, MEXU); Alto Lucero, alrededores de Laguna Verde, 18 Nov 1975, *Dorantes et al. 5209* (MEXU); Camarón de Tejeda, Matta de Agua, 13 Aug 1926, *Fisher s.n.* (DS); Veracruz, Villarin, 5 km al W del Puerto, 2 Jul 1990, *García Bielma 536* (MEXU); a 4 km de Paso de Macho, hacia Camarón, 12 Jul 1970, *González G. 133* (MEXU); La Antigua, Salmoral, camino a los cañales, a 1 km de Salmoral, 6 Apr 1988, *González H. 267* (MEXU); Veracruz, Rancheria Neveria, carretera antigua nacional Xalapa-Veracruz km 90.5, 10 Sep 1981, *Gutiérrez B. 723* (MEXU); Soledad de Doblado, Paso de Conejos, [Paso de los Cedros], 6 Aug 1966, *Hernández Magaña 207* (DS, MEXU, NY); Colipa, 1841, *Karwinski 1399* (LE); Cuitláhuac, along route 150 just NW of Cuitláhuac, 6 Jun 1960, *King 2669* (DS, NY); Veracruz, along route 180, about 14 miles S of Veracruz, 7 Jun 1960, *King 2701* (DS, NY); Puente Nacional, Jun 1838, *Linden 243* (K); Emiliano Zapata, carretera Rinconada-Buenavista, 13 Oct 1999, *Lizama M. 1479* (MEXU); hacia el Plan de Las Hayas km 739-761, 13 Aug 1969, *Lot 454* (CAS, MEXU); Actopán, La Mancha, desviacion de la carretera J. Cardel a Nautla, cerca de Ursulo Galvan, 29 Nov 1969, *Lot 483* (CAS, MEXU); Veracruz, Santa Rita, 600 m al NW de la estación de Santa Rita (agricultura prehispanica), 19 Oct 1987, *Luna M. 36* (MEXU); Veracruz, 1 km de Santa Elena, 13 May 1988, *Luna M.* & *Zola B. 262* (MEXU); Emiliano Zapata, Palmar, and vicinity, 3 Sep 1935, *MacDaniels 443* (BH); 15 km S of Vega de la Torre, 18 Nov 1963, *MacKee, H.S. 10974* (K); Emiliano Zapata, alrededores del poblado de la Balsa, 25 May 1982, *Marquez, M. 1095* (MEXU); 10 km S of Nautla, 18 Nov 1963, *McKee 10974* (MEXU); Paso de Ovejas, 1 km al N de Cantarranas, 25 Apr 1985, *Medina A.* & *Acevedo R. 24* (MEXU); Totutla, along Hwy 144 W of Mata Oscura, km 36-37, 25 Jul 1965, *Mertz 148* (MEXU); Puente Nacional, 6 km E of Banos de Carrizal (downstream), on N side of valley of Río Chico [upper part of Río de La Antigua], 8 km ESE of Emiliano Zapata [=Carrizal], 17 Apr 1983, *Nee* & *Taylor 26632* (BH, BM, CAS, CORD, MEXU, NY); 20 km al N de Martinez de la Torre, rumbo a Papantla, 5 Sep 1967, *Nevling* & *Gomez-Pompa 544* (MEXU); Juchique de Ferrer, 22 km de Palma Sola, rumbo a Plan de las Hayas, 16 Apr 1969, *Nevling* & *Gomez-Pompa 1043* (MEXU); Cardel, a 500 m del puente del Río La Antigua, 27 Oct 1988, *Orea L. 153* (MEXU); Actopán, 5 km antes de la desviación a san Nicolas, 5 Aug 1976, *Ortega 427* (K, MEXU); Rancho La Palmilla, Oct 1929, *Purpus s.n.* (DS); near Rancho Camarones, Feb 1930, *Purpus 11048* (K); Emiliano Zapata, Chavarillo, 22 Oct 1930, *Reddick 157* (BH); Axocuapán, camino Coetzalán-Cueva del Abono, Ejido de Coetzalán, 14 Jan 1984, *Robles H. 397* (MEXU); Acultzingo, Cumbres de Acultzingo 30km carretera Orizaba-Tehuacan, D-8-50, 30 Nov 1967, *Rosas R. 876* (BM); Emiliano Zapata, La Laja, entre Corral Falso-Pinoltepec, a 900 m de al carretera Jalapa [Xalapa]-Veracruz, desv. a 16 km a SE de Jalapa [Xalapa], 20 Sep 1975, *Sousa S.* & *Ramos 4836* (MEXU); La Laja-Pinoltepec, 6 Mar 1976, *Trejo, L. 13* (MEXU); Ursulo Galvan, 31 Mar 1971, *Ventura A. 3385* (DS); Dos Ríos, Palo Gacho, 17 Sep 1974, *Ventura A. 10560* (MEXU); Dos Ríos, Carrizal, 25 Jul 1975, *Ventura A. 11718* (MEXU); La Antigua, 700 m antes de la playa (agricultura prehispanica), carretera Cardel-Veracruz, camino de terraceria que sale a la playa a 500 m antes de la caseta de cobro, 21 Sep 1987, *Zamora C. 598* (MEXU); Emiliano Zapata, carretera a Lencero, 28 Jul 1976, *Zola B. 578* (MEXU); Paso de Ovejas, carretera El Faisán-La Víbora, 500 m de El Faisán, 16 Jun 1987, *Zola B.* & *Mota 2135* (MEXU). **Yucatán**: a 14 km al O de Chemax, sobre la carretera Valladolid-Cancún, 16 Jul 1985, *Cabrera & de Cabrera 8895* (CIQR, MEXU); Mayapan, alrededores de la zona arqueológica de Mayapan, a 1 km al S de Telchaquillo, carretera Tecch-Oxkutzoab, 21 Jul 1985, *Cabrera & de Cabrera 9130* (CIQR, MEXU); a 2 km al S del crucero Las Coloradas-San Felipe, sobre la carretera Tizimín-Río Lagartos, 20 Dec 1985, *Cabrera & de Cabrera 10064* (MEXU); 10 km al E de Telchak Puerto, sobre la carretera Pto. Progreso-Dzilam de Bravo, 23 Jan 1986, *Cabrera & de Cabrera 10755* (MEXU, NY); a 4 km de Telchak Puerto sobre el camino a Telchak Pueblo, 18 Apr 1986, *Cabrera & de Cabrera 11289* (CIQR, MEXU); 2 km al O de Ticuch, sobre la carretera 180 tramo Cancún-Valladolid, 21 Jul 1986, *Cabrera & de Cabrera 11530* (MEXU); 3 km al E del crucero Río Lagartos-San Felipe, sobre el camino a Las Coloradas, 22 Jul 1986, *Cabrera & de Cabrera 11566* (MEXU); Gruta de Blanacanche, 32 km al O de Valladolid, en los alrededores de la gruta, 23 Jul 1986, *Cabrera & de Cabrera 11625* (MEXU); Progreso, 6 km al O del Puerto Progreso, sobre el camino a Yucalpeten, 24 Nov 1986, *Cabrera & de Cabrera 12835* (CIQR, MEXU); Motul, 4 km al O de Motul, sobre el camino a Mérida, 21 Mar 1988, *Cabrera & de Cabrera 15787* (CICY, MEXU); carretera Komchén-Cosgaya, 28 Apr 1992, *Campos* & *Simá 2800* (MEXU); Progreso, km 10-16 carretera Sierra Papacal a Chuburná Puerto, 28 Apr 1992, *Campos* & *Simá 2836* (MEXU); Muna, km 8 carretera a Opichén, 24 Sep 1984, *Chan 3983* (CICY, MEXU); Río Lagartos, entronque de la carretera Río Lagartos a Las Coloradas, 13 Mar 1985, *Chan 4835* (MEXU); Ticul, Hacienda Yokat, camino Muna-Ticul, 29 Apr 1985, *Colunga 155* (MEXU); Cuzamá, 4 km al S de Chunkanán, en un cenote, 19 Nov 1995, *Dorantes* & *Ek 34* (CICY); Hunucmá, 6 km de Hunucmá rumbo a Sisal, 10 Feb 1992, *Durán et al. 1581* (MEXU); Mérida, junto a la discoteca Bin Bon Beach, 26 Jun 1992, *Durán* & *Méndez 1626 B* (CIQR, MEXU); San Felipe, 100 m al E del desvio de San Felipe a Río Lagartos, 1 Jul 1999, *Durán et al. 3328* (MEXU); Tekon, 30 Sep 1955, *Enríquez 98* (MEXU); Chuburná, 18 Jul 1956, *Enríquez 686* (MEXU); 7 km S Xtampu to San Diego Guerrera, 18 Feb 1991, *Escalante R. et al. 240* (K); Mérida, Dzilbilchaltún, 27 Sep 1982, *Escalante 672* (CIQR, MEXU); Sinanché, a 13 km de Telchac Puerto camino a Dzilam, 17 Oct 1980, *Espejel 99* (MEXU); Abalá, 2 km al W de Mucuyché, 31 Mar 1993, *Feliciano Kú y Yam 254* (MEXU); Telchac Puerto, 10 km al W de Telchac Puerto, 9 Dec 1996, *Feliciano Kú y Yam 626* (MEXU); Izamul, *Gaumer 366* (BM, CAS, DS, E, G, K, LE, NY, W); Dzidzantún, Mina de Oro, May 1916, *Gaumer and sons 23325* (K, NY); Yaxcabá, Stancabxonot, 1917, *Gaumer 23622* (LE); Dzoncauich, a 10 km de Chacmay, 12 Apr 1983, *Góngora 301* (MEXU); Tixkokob, cerca del poblado Ruinas de Ake, 28 Jun 1983, *Góngora 694* (CICY, MEXU); Tinum, 1 km al E de Chichen Itza, 30 Aug 1991, *Méndez et al. 261* (MEXU); Mérida, Sierra Papacal, 2 km al N, 22 Apr 1993, *Méndez et al. 812* (MEXU); Mérida, ruinas de Dzibilchaltun [Chablekal], 4 Apr 1981, *Narváez et al. 339* (CIQR); Sacalum, camino Sacalum-Mérida, 10 Jul 1981, *Narváez* & *Puch 590* (CICY); Mérida, Opichén, Hcda. Xixim, 10 Feb 1983, *Orellana 119*-*BB* (MEXU); Cantamayec, 6 km S Tixcalcupil, 5 Mar 1996, *Ortíz M. s.n.* (MEXU); entrada a la zona arqueológica de Mayapán, 11 Mar 1999, *Peña-Chocarro* & *Tun 407* (BM, MEXU); Dzemul, 4 km al S de Dzemul, 4 Sep 1991, *Pérez Lara 94* (MEXU x2); Buctzotz, a 15 km al E de Buctzotz, 5 Jun 1992, *Pérez Lara 186* (MEXU); Abalá, 1 km al W de Hacienda Uayaicén, 30 Oct 1996, *Pérez Lara 612* (MEXU); Tecóh, 1 km al S de Lepan, 2 Aug 1999, *Pérez Lara 860* (MEXU); Hunucmá, 10 km al SE de Sisal, 9 Nov 1999, *Pérez Lara 892* (MEXU); Abalá, 4 km al E de Tenozón, 14 Aug 1979, *Pérez S. et al. 318* (MEXU); Oxkutzcab, salida de Oxkutzcab yendo a Lol-Tun, 7 Jul 1981, *Puch* & *Narváez 447* (CIQR, MEXU); Temox, km 17 carretera Izamal-Temax, Dzoncauich, 25 Apr 1984, *Puch* & *Ortiz 1319* (CICY); Hunucmá, al 8 km al S de Hunucmá, 19 Mar 1992, *Reyes de los Santos 147* (MEXU); Tunkás, a 5 km al W de Tunkás, 23 Sep 1993, *Reyes de los Santos 272* (MEXU); Timucuy, 2 km al E de Timucuy, 24 Aug 1994, *Reyes de los Santos 367* (MEXU x 2); Yaxkukul, 1 km al N de Yaxkukul, 30 Aug 1995, *Reyes de los Santos 477* (MEXU); Panabá, 2 km al S de Panaba, 25 Apr 1996, *Reyes de los Santos 545* (MEXU); Chicxulub Pueblo, 5 km al N de Chicxulub, 2 Dec 1998, *Reyes de los Santos, E. 788* (MEXU); Peto, 1 km al N de Peto, 30 Sep 1999, *Reyes de los Santos 871* (MEXU); a 1.5 km al S de Conkal, 7 Jun 1984, *Rivera 90* (CIQR); Xpacamul, Maya, Mérida, 29 Jul 1865, *Schott 310* (BM); Tinum, Chichen Itzá, 8 Jun 1932, *Steere 1065* (BM, MEXU); Dzemul, km 6 de al carretera Dzemul-Xtampú, 4 km S del entronque a las ruinas de Xtampú, 3 Dec 2004, *Tapia et al. 1533* (MEXU); Baca, 8, 5 km al N de Baca, camino a ranchos ganaderos, 10 Dec 1998, *Tun* & *González-Iturbe 443* (MEXU); Tepakan, carrretera Izamal sobre km 6 rumbo a Tepakan, 18 Jan 1982, *Ucán Ek 1826* (MEXU); Valladolid, Pixoy, calle N de la plaza principal, 12 Oct 1983, *Ucán Ek et al. 3021* (CIQR, MEXU); Mérida, Parque Científico, a 5 km de Sierra Papacal, 5 km al oeste, 14 Aug 2013, *Uh Cauich et al. 4* (MEXU); Yaxcabá, Tixcacaltuyub, 27 Jun 1980, *Vargas 68* (MEXU); Chac-May, 5 Dec 1985, *Waizel Bucay s.n.* (MEXU); 10 km N of Sinanche, 1 km E into Rancho Chumhabin [Chum Habin], 15 Feb 1994, *Way 107* (CICY, K); Chicxulub Pueblo, ca. 4 mi SE of Chicxulub Puerto, 30 Jul 1972, *Webster et al. 17551* (MEXU); N slopes of hills S of Ticul, 22 Feb 1982, *White* & *Mott 96* (MEXU). **Zacatecas**: Moyahua, Los Otates, 20 Aug 1992, *Enríquez E. 114* (MEXU); Apozol, desviación presa Achoquén (projecto Cañon de Juchipila), 3 Aug 1995, *Enríquez E. 465* (MEXU); Juchipila, Apozol, 20 km al N de Juchipilas, 16 Aug 1984, *Hernández Magaña et al. 9027* (MEXU).

#### 5. Solanum mortonii

Taxon classificationPlantaeSolanalesSolanaceae

Hunz., Kurtziana 12-13: 133, fig. 1. 1979.

Figures 2F , 3 C, D , 11

##### Type.

Argentina. Catamarca: Dpto. Capayán, San Pablo, between Concepción and Huillapima, 700 m, *A. Hunziker & R. Subils 22764* (lectotype, designated by Chiarini 2013, pg. 221: CORD [CORD00004247]; isolectotype: CORD [CORD00004248]).

##### Description.

Erect rhizomatous shrub, to 1 m tall. Stems erect, woody, armed or unarmed; young stem densely stellate-pubescent, the trichomes multangulate, translucent, short-stalked, the rays 10–12, ca. 0.5 mm long; prickles if present 5–6 mm long, needle-like and straight, pale yellowish brown; bark smooth, brown or yellowish brown from persistent pubescence. Sympodial units difoliate, not markedly geminate. Leaves simple, (2-)4–9 cm long, (1-)2–4 cm wide, elliptic, ca. 3 times longer than wide, discolorous, drying yellowish green to greyish green; adaxial surfaces densely stellate-pubescent but the leaf blade tissue visible, the trichomes multangulate, translucent, sessile, the rays 10–12, ca. 0.5 mm long; abaxial surfaces more densely pubescent with similar multangulate trichomes; principal veins 5–7 pairs, impressed adaxially, flat abaxially, spreading at ca. 45° to the midvein, the tertiary venation usually visible in dry material; base truncate to slightly cordate, often somewhat oblique; margins shallowly lobed, the lobes 4–7 on each side, of varying sizes, becoming smaller towards the leaf apex, the sinuses extending only 1/4 or less of the distance to the midvein, triangular; apex acute to somewhat rounded, rarely obtuse; petiole 1–2 cm long, densely stellate-pubescent like the young stem, unarmed. Inflorescences terminal or lateral, to 6.5 cm long, with up to 10 flowers, unbranched; peduncle 1.5–3 cm long, densely pubescent with multangulate trichomes like those of the stems; pedicels 0.4–1 cm long, ca. 1.5 mm in diameter, robust, articulated less than 0.5 mm from the base, densely pubescent like the leaf blade; pedicel scars prominent, spaced ca. 0.5 cm apart. Buds turbinate, the corolla strongly exserted from the calyx tube prior the anthesis. Flowers 5-merous, strongly heteromorphic and the plants andromonoecious, only the basal flower perfect (hermaphroditic), the distal flowers functionally staminate and short-styled. Calyx conical or cup-shaped, the tube 5–6 mm long, strongly keeled, the lobes 7–10 mm long, ca. 1.5 mm wide at base, subulate, densely pubescent abaxially with multangulate trichomes. Corolla 3–3.5 cm in diameter, pentagonal, pale lavender, drying pale brown, barely lobed, interpetalar tissue abundant, the lobes ca. 0.2 cm long, ca. 0.1 cm wide, mere acumens, densely pubescent with multangulate trichomes abaxially along the midveins and surfaces exposed in bud. Stamens equal or very slightly unequal and the adaxial anthers slightly shorter; filament tube ca. 0.5 mm, free portion of the filaments ca. 2 mm; anthers 7–12 mm long, free, occasionally very slightly unequal, poricidal at the tips, the pores about the same diameter as the anther apices, clearly delineated, the anther surface smooth to finely papillose. Ovary globose, glabrous or minutely glandular puberulent; style of hermaphroditic flowers ca. 1 cm long, glabrous; stigma capitate, papillose. Fruit a globose berry, 1 per infructescence, ca. 2 cm in diameter when dry, the pericarp thin, smooth, glabrous, light green, when immature with dark stripes or marbled pattern, drying dark brown or brown-reddish and shattering; fruiting pedicels 1–1.7 cm long, ca. 1.5 mm in diameter at base, ca. 2.5 mm at apex, woody, erect, sparsely armed with straight yellowish red prickles or unarmed; fruiting calyx somewhat accrescent, to 2 cm long, the lobes covering up to 1/3 of the mature fruit, usually unarmed, if prickly the prickles needle-like and straight. Seeds ca. 40 per berry, 4–5 mm long, 3–4 mm, flattened, reniform-rounded, black, the surfaces minutely pitted. Chromosome number: not known.

##### Distribution

(Figure 12). *Solanum mortonii* is endemic to northern Argentina in the Province of Catamarca at 600 to 1000 m elevation.

##### Ecology and habitat.

Like some other members of the group, *S. mortonii* has rhizomatous stems (see Fig. 4C) and is apparently clonal; it grows on rocky shaded or open banks in dry forests and can form large colonies.

##### Common names and uses.

None recorded.

##### Preliminary conservation status

(IUCN 2016). EN (Endangered). EOO = 1,115 km^2^ (EN – Endangered); AOO = 32 km^2^ (EN – Endangered). *Solanum mortonii* has a very narrow distribution along the Sierra de Ambato and is not common where it occurs. It does, however, appear to reproduce vegetatively through underground stems, so may persist well within this narrow range.

##### Discussion.

*Solanum mortonii* is a striking species with dense greyish silvery pubescence. It is sympatric with *S. elaeagnifolium*, and differs from that species in its multangulate, rather than lepidote, trichomes, its broader leaves and strongly andromonoecious habit. This species is restricted to very small area in the eastern part of Catamarca province, Argentina. During attempts to germinate seeds in order to perform mitotic chromosome counts number from rootlets, all seeds collected proved to be inviable. This suggests that *S. mortonii* has some type of reproductive abnormality or that seeds have very time-limited viability.

##### Specimens examined.

**Argentina**. **Catamarca**: Capayán, San Pablo, a 1 km saliendo San Pablo rumbo a Concepción, 23 Feb 2003, *Barboza et al. 633* (CORD); Capayán, San Pablo, a unos 100 m de la Iglesia San Pablo rumbo a Huillapima, 23 Feb 2003, *Barboza et al. 639* (CORD); Capayán, subiendo a la Cuesta de los Angeles, entre km 12/13, 24 Feb 2003, *Barboza et al. 644* (CORD); Capayán, San Pablo, pasando el Río (vado) de San Pablo, 10 Feb 2012, *Barboza et al. 3437* (BM, CORD); Capayán, San Pablo, entre la Iglesia y el Río (vado) de San Pablo, 10 Feb 2012, *Barboza et al. 3438* (BM, CORD); Capayán, desde Miraflores rumbo a Los Angeles, a 19 km de Los Angeles, 10 Feb 2012, *Barboza et al. 3439* (BM, CORD); La Paz, El Potrero, 23 Apr 1947, *Brizuela 1181* (W); Capayán, Concepción, Quebrada El Totoral, 10 Jan 1909, *Castillón 1030* (LIL); Capayán, San Pablo, Sierra Ambato, 3 Feb 1910, *Castillón 1628* (LIL); Capayán, Sierra de Ambato, falda E, subiendo por la Quebrada de Simbolar desde Concepción hacia La Abuelita, 21 Nov 1965, *Hunziker et al. 18322* (CORD, NY, US); Capayán, Sierra de Ambato, falda E, subiendo por la cuesta entre Miraflores y Los Angeles, 27 Nov 1965, *Hunziker et al. 18333* (CORD, NY, US); Capayán, Sierra de Ambato, falda E, subiendo por la cuesta entre Miraflores y Los Angeles, 16 Mar 1972, *Hunziker 21889* (CORD); Capayán, Sierra de Ambato, falda E, subiendo por la cuesta entre Miraflores y Los Angeles, 16 Mar 1972, *Hunziker 21895* (CORD, NY).

#### Doubtful names and names not validly published

*Solanum ocoapense* Sessé & Moc., Fl. Mex., in La Naturaleza, ser. 2, Suppl. part 5: 1894.

Type. “Habitat in Ahualulci montibus” [México. Tabasco: Ocuapan, 17°51'N, 93°29'W, or San Luis Potosí: Ahualulco, 22°24'N, 101'10"W], *M. Sessé & J.M. Moçino s.n.* (MA?). This name possibly refers to *S. houstonii* or *to* *S. lanceolatum* Cav. of the Torva clade (see Knapp 2008b). No specimens were located by McVaugh (2000) or Knapp (2008b), so it remains *incertae sedis* (a name of uncertain application).

*Solanum polygamum* Boiss. ex Dunal, Prodr. [A. P. de Candolle] 13(1): 334. 1852. pro syn. S. tridynamum var. stylosum Dunal = *S. houstonii* Martyn

*Solanum uniflorum* Meyen ex Nees, Nova Acta Phys.-Med. Acad. Caes. Leop.-Carol. Nat. Cur. 19, Suppl. 1: 388. 1843. pro syn. *S. elaeagnifolium* Cav. = *S. elaeagnifolium* Cav.

*Solanum violaceum* Dunal, Prodr. [A. P. de Candolle] 13(1): 334. 1852. pro syn. S. tridynamum var. anoplocladum Dunal = *S. houstonii* Martyn

**Figure 13.**
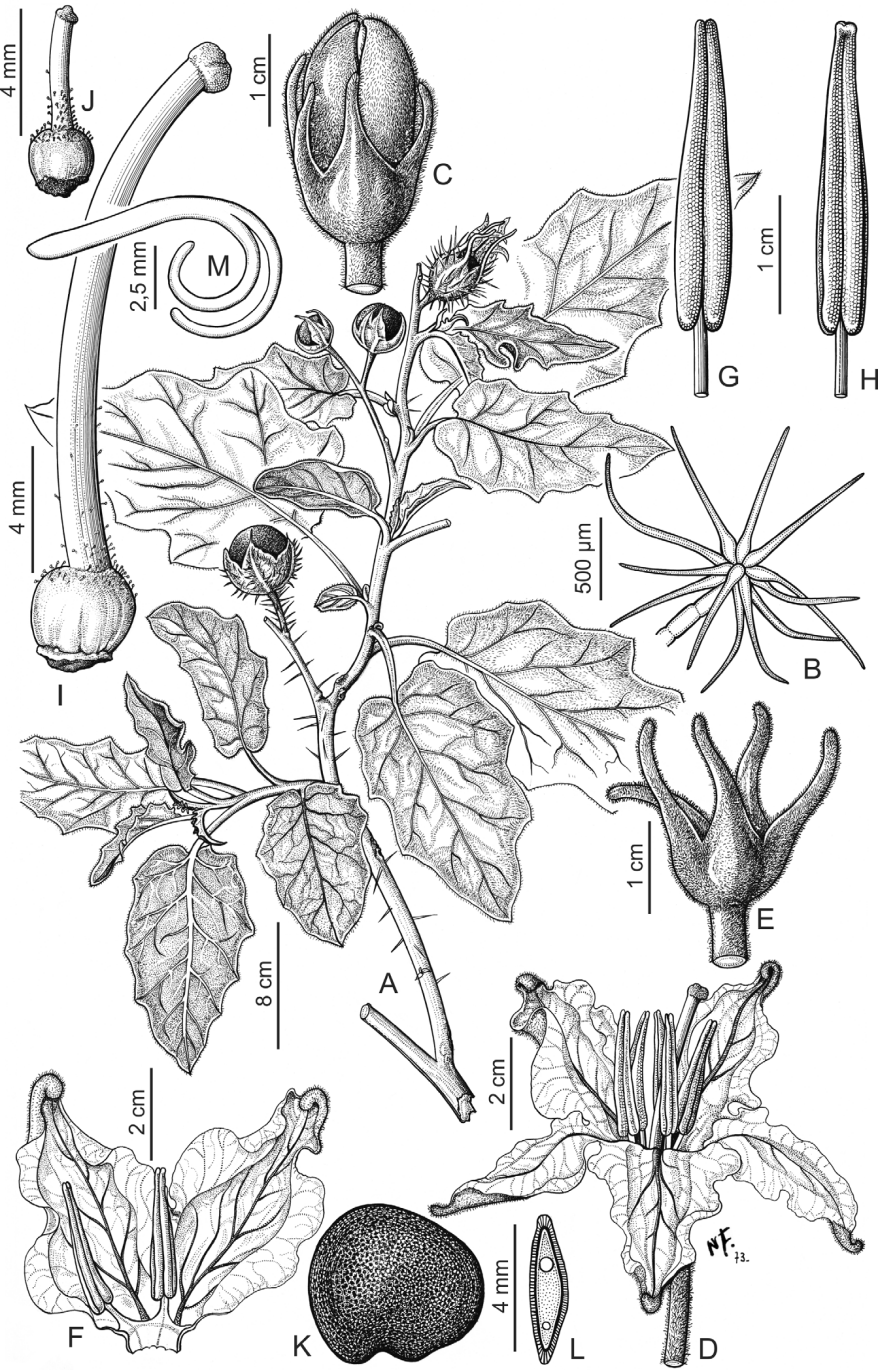
*Solanum mortonii* Hunz. **A** Fruiting branch **B** Stellate trichome of leaf **C** Floral bud (showing elongate calyx acumens) **D** Flower **E** Calyx **F** Spread corolla **G** Dorsal view of stamen **H** Ventral view of stamen **I** Gynoecium of long-styled flower **J** Gynoecium of short-styled flower **K** Seed **L** Transverse section of seed **M** Embryo. Drawn by Nidia Flury. **A–F, I–L** from *Hunziker 22764* (CORD) **G, H** from *Hunziker 21869* (CORD). Reproduced with permission from Flora Argentina (Chiarini 2013).

## Supplementary Material

XML Treatment for Solanum

XML Treatment for Solanum elaeagnifolium

XML Treatment for Solanum hindsianum

XML Treatment for Solanum homalospermum

XML Treatment for Solanum houstonii

XML Treatment for Solanum mortonii

## Appendix 1

### Index to numbered collections

Only first collectors in collections made by two or more collectors are listed here; pairs of collectors are listed in the Specimens examined section, while groups of three or more are listed as *et al*. Collections by anonymous collectors without date or other identifying features are not listed here. Full collector strings can be found on Solanaceae Source (http://www.solanaceaesource.org) or on the NHM Data Portal (http://dx.doi.org/10.5519/0007624).

Abd El Ghani, M. 6908 (*elaeagnifolium*).

Abrams, L. 6607 (*elaeagnifolium*).

Abulaila, K. JOR3-1 (*elaeagnifolium*).

Acevedo, R. 32 (*elaeagnifolium*).

Ackermann, M. 218 (*elaeagnifolium*).

Acosta, L.E. 21 (*houstonii*).

Acosta, M. 663 (*houstonii*).

Aguayo C, J.A. 2 (*houstonii*).

Aguiar H, H. 59 (*houstonii*).

Aguilar C, A. 164 (*elaeagnifolium*).

Aguilar Zepeda, J.A. 344 (*houstonii*).

Aguilar, L.M. 912 (*elaeagnifolium*).

Aguilar, R.M. 425, 555 (*elaeagnifolium*).

Aguirre T., S. 45, 116, 116, 151, 259 (*elaeagnifolium*).

Agúndez, J. 132, 297 (*hindsianum*).

Ahles, H.E. 56834 (*elaeagnifolium*).

Alba, A. de 45 (*elaeagnifolium*).

Alcorn, J.B. 2028 (*houstonii*).

Altamirano, F. 1678 (*houstonii*); 1736 (*elaeagnifolium*).

Álvarez, R. 401, 499, 573, 631, 989 (*elaeagnifolium*).

Ambrosetti, J.A. 1418, 1472, 1480, 1481, 1483 (*elaeagnifolium*).

Anderson, W.R. 3504 (*elaeagnifolium*).

Araque M., J. 19Ar341 (*elaeagnifolium*).

Arenas, P. 2360 (*elaeagnifolium*).

Argañaras, J.L. 51 (*elaeagnifolium*).

Argüelles, E. 23, 2468 (*elaeagnifolium*).

Ariza Espinar, L. 1575, 2393, 3587, 3588b (*elaeagnifolium*).

Armenta, P.M.A. 132 (*houstonii*).

Arnoldo-Broeders, M. 3621 (*elaeagnifolium*).

Arsène, G. 10632a (*elaeagnifolium*).

Arteaga Saucedo, M.C. 840 (*elaeagnifolium*).

Articó, L. 241, 244, 250 (*elaeagnifolium*).

Atwood, N.D. 11023, 16071, 28852 (*elaeagnifolium*).

Austin, S.B. 277 (*elaeagnifolium*).

Bacigalupi, R. 2663 (*elaeagnifolium*).

Bailey, L.H. 21 (*houstonii*); 204 (*hindsianum*).

Baines, R. 255 (*elaeagnifolium*).

Baird, G.I. 1818 (*elaeagnifolium*).

Baker, M.A. 7568, 7581 (*hindsianum*).

Baker, M.S. 11244, 11574 (*elaeagnifolium*).

Balegno, B. 249 (*elaeagnifolium*).

Balls, E.K. 10857 (*elaeagnifolium*).

Barbour, M. 119 (*elaeagnifolium*).

Barboza, G.E. 76, 220 (*elaeagnifolium*); 633, 639, 644 (*mortonii*); 1923, 1944, 2010, 2308, 2319, 2321, 2334, 2340, 3084, 3131, 3159, 3160, 3246, 3434, 3435 (*elaeagnifolium*); 3437, 3438, 3439 (*mortonii*); 3449, 4120, 4337, 4450 (*elaeagnifolium*).

Barclay, G.W. 3079 (*hindsianum*).

Barkley, F.A. 14A539, 783 (*elaeagnifolium*); 2616, 2617, 7505 (*houstonii*).

Baro, D. 495 (*elaeagnifolium*).

Barr, R.J. 61-121 (*houstonii*).

Bartholomew, B. 2430 (*elaeagnifolium*); 3643 (*houstonii*).

Bartlett, H.H. 10991, 19760, 19871, 19886, 19905 (*elaeagnifolium*).

Beatley, J. 9028 (*elaeagnifolium*).

Beetle, A.A. 26132 (*elaeagnifolium*).

Bell, W.M. 48 (*elaeagnifolium*).

Benedeto, ? 235 (*elaeagnifolium*).

Benítez-Paredes, A. 1814 (*elaeagnifolium*); 3405 (*houstonii*).

Bennett, H.R. 8486, 8659 (*elaeagnifolium*).

Berg, C.C. 153 (*elaeagnifolium*).

Bernardello, G. 449, 503, 522, 534, 746, 754, 788 (*elaeagnifolium*).

Berro, M.B. 4411, 7942 (*elaeagnifolium*).

Berry, E.O. 19, 34 (*hindsianum*).

Biloni, J.S. 175M (*elaeagnifolium*).

Biltmore Herbarium 3438 (*elaeagnifolium*).

Bisschoff, J. 1 (*elaeagnifolium*).

Biurrun, F. 2103, 2729, 7225 (*elaeagnifolium*).

Blakley, E.R. 3-1652 (*houstonii*).

Blanchet, J.S. 6410 (*elaeagnifolium*).

Blumer, J.C. 1732 (*elaeagnifolium*).

Bodenbender, W. 55 (*elaeagnifolium*).

Boege, W. 689 (*houstonii*); 2793 (*elaeagnifolium*).

Boelcke, O. 1412, 11691 (*elaeagnifolium*).

Bojórquez B., G. 721 (*houstonii*).

Borsini, O.H. 1013, 1201, 1333 (*elaeagnifolium*).

Botello, L. 14 (*elaeagnifolium*).

Boulos, L. 18198 (*elaeagnifolium*).

Bowen, J. 27 (*elaeagnifolium*).

Boyd, S. 5335, 5539, 5778, 5956 (*hindsianum*).

Bradburn, A.S. 1134 (*houstonii*).

Braunton, E. 697 (*elaeagnifolium*).

Breedlove, D.E. 1150 (*elaeagnifolium*); 1370, 1455, 1473 (*hindsianum*); 1511 (*houstonii*); 60903, 60957 (*hindsianum*); 62857 (*houstonii*); 71604, 71795 (*hindsianum*).

Brigada Zacatecas 86 (*elaeagnifolium*).

Brizuela, A. 1181 (*mortonii*).

Brizuela, J. 847, 1159 (*elaeagnifolium*).

Brother Anect 115 (*elaeagnifolium*).

Broome, C.R. 313 (*elaeagnifolium*).

Bruno, R. 4 (*elaeagnifolium*).

Burge, A. 25 (*elaeagnifolium*).

Burkill, I.H. 432 (*elaeagnifolium*).

Búrquez, A. 96-15, 94-017 (*houstonii*); 2006-54, 2006-89 (*hindsianum*); 95-154 (*houstonii*); 91-316 (*hindsianum*); 96-960, 96-1127 (*houstonii*).

Bush, B.F. 266, 392, 1588 (*elaeagnifolium*).

Butterwick, M. 7782, 8217 (*elaeagnifolium*).

Bye, R. 28334 (*elaeagnifolium*).

Cabrera, A.L. 7007, 30038, 30771, 31065 (*elaeagnifolium*).

Cabrera, E. 4658, 8272, 8658, 8834, 8895, 8962, 9130, 9225, 9428, 9631, 10064, 10207, 10286, 10461, 10755, 11000, 11289, 11465, 11530, 11566, 11625, 11686, 11770, 12835, 13037, 13210, 13635, 13734, 13920, 14204, 14318, 14535, 15400, 15787, 17207 (*houstonii*).

Cadena, B. 23 (*elaeagnifolium*).

Calhoun, D. 129 (*elaeagnifolium*).

Calzada, J.I. 1837, 3182, 6459, 7725 (*houstonii*); 24391 (*elaeagnifolium*); 25037 (*hindsianum*); 25305 (*elaeagnifolium*).

Campos, G. 2800, 2836 (*houstonii*).

Cancino, J. 20, 55, 123 (*hindsianum*).

Canedo, F. 9075 (*elaeagnifolium*).

Cano R, P. 21 (*elaeagnifolium*).

Cantero, J.J. 6183 (*elaeagnifolium*).

Cantino, P.D. 505 (*elaeagnifolium*).

Carette, E. 22 (*elaeagnifolium*).

Carleton, M.A. 171 (*elaeagnifolium*).

Carnegie, F.G. 40 (*elaeagnifolium*).

Carranza, E. 3292 (*houstonii*).

Carrasco, E.L. 151 (*houstonii*).

Carrillo-Reyes, P. 1974 (*hindsianum*); 4139 (*houstonii*).

Carter, A. 1892 (*hindsianum*).

Castañeda V, ? 64, 68 (*elaeagnifolium*).

Castellanos, A. 90 (*elaeagnifolium*); 206 (*houstonii*); 389, 806 (*elaeagnifolium*).

Castello, L.V. 232 (*elaeagnifolium*).

Castillo Campos, G. 189, 700, 841, 6971, 18152 (*houstonii*).

Castillón, L. 1030, 1628 (*mortonii*); 1779 (*elaeagnifolium*).

Cervantes M., H. 19 (*houstonii*); 58 (*elaeagnifolium*).

Chambers, K.L. 720, 771 (*hindsianum*).

Chan, C. 45, 100, 1827, 2689, 3166, 3983, 4835 (*houstonii*).

Chapiro, G. 41 (*houstonii*).

Charlton, D. 3259 (*elaeagnifolium*).

Chaudhary, S.A. E455 (*elaeagnifolium*).

Chavelas P., J. ES-3971 (*houstonii*).

Chávez Montes 82 (*houstonii*).

Chehaiber, T. 201 (*houstonii*).

Chiapella, J. 1804, 2175, 2186 (*elaeagnifolium*).

Chiarini, F. 40, 462 (*elaeagnifolium*); 505 (*homalospermum*); 507, 558, 559, 565, 566, 736, 781, 824[a], 824, 828, 879, 890, 892, 899, 917, 928, 1026, 1028, 1030, 1033, 1034, 1039, 1270 (*elaeagnifolium*).

Christ, J. 991 (*elaeagnifolium*).

Cirujano, S. R-10431 (*elaeagnifolium*).

Claraz Schenkung, A. 81 (*elaeagnifolium*).

Clark, W.H. 3289, 3304 (*hindsianum*).

Clausen, J. 595 (*elaeagnifolium*).

Clausen, R.T. 4597 (*elaeagnifolium*).

Clokey, I.W. 5937 (*elaeagnifolium*).

Clover, E.U. 165 (*elaeagnifolium*).

Cochrane, T.S. 8516 (*houstonii*).

Cocucci, A.A. 220, 369, 439, 440, 977, 1011, 1014, 1364, 3848, 5014, 5041, 5760 (*elaeagnifolium*).

Cocucci, A.E. 14 (*elaeagnifolium*).

Colunga, P. 155 (*houstonii*).

Compling, M. 9 (*elaeagnifolium*).

Constance, L. 3116 (*hindsianum*).

Copp, J.F. 70-7 (*hindsianum*).

Cory, V.L. 52468 (*elaeagnifolium*).

Cosa, M.T. 44, 108, 115, 124, 162, 182, 186 (*elaeagnifolium*).

Coulter, T. 1246, 1247, 1248 (*elaeagnifolium*); 1251 (*houstonii*).

Crampton, B. 7136 (*elaeagnifolium*).

Croat, T.B. 39945 (*houstonii*).

Cronemiller, F.P. 3061 (*hindsianum*).

Crosswhite, F.S. 720 (*elaeagnifolium*).

Cruden, R.W. 1174 (*houstonii*).

Cruz Durán, R. 4737, 5483 (*houstonii*).

Cuesta, L.R. 57 (*elaeagnifolium*).

Cuezzo, A.R. 872, 898, 1058, 1302, 2227, 2254 (*elaeagnifolium*).

Cuming, H. 169, 1090 (*elaeagnifolium*).

Curtiss, A.H. 5913 (*elaeagnifolium*).

D’Arcy, W.G. 44A, 1974, 4861, 11689, 12027 (*elaeagnifolium*).

D´Angelo, C. 285 (*elaeagnifolium*).

Daniel, T.F. 6784, 6827 (*hindsianum*).

Daveau, J. 2260 (*elaeagnifolium*).

Davidse, G. 9252 (*elaeagnifolium*).

Davis, P.H. 66299 (*hindsianum*).

Davis, T. 677, 1000 (*elaeagnifolium*).

Deaver, C.F. 6439 (*elaeagnifolium*).

Del Vitto, L.A. 850, 3558 (*elaeagnifolium*).

Dells, A. 3247 (*elaeagnifolium*).

Demaree, D. 7674, 10857, 17916 (*elaeagnifolium*).

Di Fulvio, T.E. 799, 823, 825, 831, 941, 942, 949, 1110, 1112, 1126 (*elaeagnifolium*).

Díaz Luna, C.L. 4141 (*hindsianum*).

Díaz Vilchis, I. 124, 438 (*elaeagnifolium*).

Dinelli, E. 654 (*elaeagnifolium*).

Dixon, D. 3636 (*elaeagnifolium*).

Doebley, J. 203 (*houstonii*).

Domingo, M.V.Z. 524 (*elaeagnifolium*).

Domínguez Cadena, R. 1237 (*elaeagnifolium*); 2420, 2696, 3063 (*hindsianum*).

Dorantes, A. 34 (*houstonii*).

Dorantes, J. 251, 260, 827, 1033, 1127, 1224, 1333, 1416, 5209, 5260 (*houstonii*).

Dottori, N.M. 138, 139, 145, 146, 148, 169, 179, 180, 186 (*elaeagnifolium*).

Drouet, F. 3695 (*houstonii*); 3885 (*hindsianum*).

Drummond, J.R. 93, 266 (*elaeagnifolium*).

Dullas, W. 135 (*elaeagnifolium*).

Dunn, D. 23177 (*elaeagnifolium*).

Dunnett, D. 13 (*elaeagnifolium*).

Durán, R. 1581, 1626B, 3328 (*houstonii*).

Dusén, P.K.H. 5217 (*elaeagnifolium*).

Dwyer, J.D. 14151 (*elaeagnifolium*).

Eastwood, A. 1040, 5958, 8550, 8627, 9301A, 11763, 13946, 15419, 15646, 17097, 18099 (*elaeagnifolium*).

Eaton 57 (*elaeagnifolium*).

Eggleston, W.W. 7424 (*elaeagnifolium*).

Eggli, U. 1958 (*hindsianum*).

Ehlers, J.H. 6410 (*elaeagnifolium*).

Ekman, E.L. 13942 (*elaeagnifolium*).

El Ghani, M.A. 6908 (*elaeagnifolium*).

Elizondo, J.L. 319 (*hindsianum*).

Elliot, B. 11929c (*elaeagnifolium*).

Elliot, C. 62 (*elaeagnifolium*).

Els, P. 3118 (*elaeagnifolium*).

Encarnación, R. 32 (*hindsianum*).

Enríquez E., E.D. 114, 465 (*houstonii*).

Enríquez, O.G. 98, 686 (*houstonii*).

Equipo 5 (*elaeagnifolium*).

Ertter, B. 2939 (*elaeagnifolium*).

Escalante R., S. (*houstonii*).

Escalante, S. 240, 549, 672, 761, 854 (*houstonii*).

Escobedo, J.M. 928, 1594 (*elaeagnifolium*).

Espejel, I. 99 (*houstonii*).

Espejo, A. 1023 (*elaeagnifolium*).

Ewan, J.A. 15, 680 (*elaeagnifolium*).

Eyerdam, W.J. 23093, 23735 (*elaeagnifolium*).

Fabris, H.A. 8357 (*elaeagnifolium*).

Farnsworth, E.L. 310 (*elaeagnifolium*).

Felger, R.S. 86-400, 85-795 (*elaeagnifolium*); 92-1031, 7071, 9242, 10542, 11613, 15319, 20226 (*hindsianum*).

Feliciano Kú y Yam, Q.B.A. 203, 254, 626, 751 (*houstonii*).

Felippone, F. 5092, 6166 (*elaeagnifolium*).

Fendler, A. 672 (*elaeagnifolium*).

Fernández Casas, F.J. 11029, 11162 (*elaeagnifolium*).

Fernandez, J.R. 156 (*elaeagnifolium*).

Ferrari, R.V. 313 (*elaeagnifolium*).

Ferris, R.S. 1153, 2302, 2720, 3133 (*elaeagnifolium*); 8561, 8634 (*hindsianum*).

Ferrucci, M.S. 2953, 2979 (*elaeagnifolium*).

Fertig, W. 22568 (*elaeagnifolium*).

Filippa, E.M. 79 (*elaeagnifolium*).

Fischer, W. 238 (*elaeagnifolium*).

Fisher, G.L. 5089 (*elaeagnifolium*).

Flores F., G. 2122 (*houstonii*).

Flores Martínez, A. 1328, 2037, 4751 (*houstonii*).

Flores, J.S. 8431, 8819 (*houstonii*).

Flores, R.M. 2 (*elaeagnifolium*).

Flores-Franco, G. 400, 421, 512 (*hindsianum*).

Floyer [Mrs] 1 (*elaeagnifolium*).

Fortuna, J. 22 (*elaeagnifolium*).

Fortunato, R.H. 9916, 9942 (*elaeagnifolium*).

Fosberg, F.R. S-3399 (*elaeagnifolium*).

Foster, R. 3935 (*elaeagnifolium*).

Frenkel, R.E. 841A (*elaeagnifolium*).

Fritsch, P. 1325 (*hindsianum*).

Frye, T.C. 2383 (*elaeagnifolium*).

Fryxell, P.A. 3706 (*houstonii*); 5131 (*elaeagnifolium*).

Fuller, T.C. 8567, 9706, 9796, 11226, 19758 (*elaeagnifolium*).

Gaitón Hinojosa, M.A. 2013-05 (*houstonii*).

Galeotti, H.G. 1160, 1168 (*houstonii*).

Galetto, L. 24, 214, 232, 237, 239, 260 (*elaeagnifolium*).

Gallegos Harking, F. 81 (*elaeagnifolium*).

Galván, M. 618 (*elaeagnifolium*).

Galván, R. 2761, 3614 (*elaeagnifolium*).

García Bielma, M.A. 536 (*houstonii*).

García E., J.D. 17 (*houstonii*).

García M., E. 322 (*elaeagnifolium*).

García P., J. 670 (*elaeagnifolium*).

García R., L.A. 1181 (*elaeagnifolium*).

García Torres, F. 34, 78, 105, 148, 183, 315 (*houstonii*).

García, A. 40 (*elaeagnifolium*).

García, E. 85 (*hindsianum*).

Garcia, E.M. 413 (*elaeagnifolium*).

García, R.J. 23 (*elaeagnifolium*).

García-Mendoza, A. 9016 (*hindsianum*).

Gardner, M.F. 58 (*elaeagnifolium*).

Gaumer, G.F. 135, 366, 1708, 23325, 23622, 24060 (*houstonii*).

Gaviño, G. 13 (*hindsianum*).

Gay, C. 834 (*elaeagnifolium*).

Genelle, P. 819 (*elaeagnifolium*).

Gentry, A. 48912 (*houstonii*).

Gentry, H.S. 357 (*houstonii*); 51-393 (*elaeagnifolium*); 930, 1294 (*houstonii*); 4037 (*hindsianum*); 5105 (*houstonii*); 7502, 7542, 7863 (*hindsianum*); 11445, 14362 (*houstonii*).

Germán, M.T. 207 (*houstonii*).

Ghiesbreght, A.B. 120 (*houstonii*).

Giacomelli, A. 88900 (*elaeagnifolium*).

Gillespie, J.W. 5222 (*elaeagnifolium*).

Gillett, J.M. 17013 (*elaeagnifolium*).

Gillies, J. ‘Solanum2’, 28, 669 (*elaeagnifolium*).

Gilmartin, A.J. 1677 (*hindsianum*).

Giorgis, M. 693 (*elaeagnifolium*).

Glass, V. 10674 (*elaeagnifolium*).

Goddard, D.R. 836 (*elaeagnifolium*).

Gold, D.B. 365 (*hindsianum*); 757 (*houstonii*).

Gómez Lorence, F. 3 (*elaeagnifolium*).

Gómez, A. 3, 13 (*elaeagnifolium*).

Gómez-Pompa, A. 1857 (*houstonii*).

Góngora, E. 59, 301, 694 (*houstonii*).

González E., M. 13B.C. (*hindsianum*).

González G., J. 133 (*houstonii*).

González H., J.E. 267 (*houstonii*).

González Medrano, F. 8003, 8013 (*elaeagnifolium*).

González Ortega, J. 56, 425, 716, 5609, 6578, 6743, 6805, 7249 (*houstonii*).

González, M. 10 (*elaeagnifolium*); 2394 (*houstonii*).

González-Medrano, F. 39, 115 (*elaeagnifolium*); 3122 (*houstonii*); 8131, 12993 (*elaeagnifolium*).

Goodman, G.J. 1142 (*elaeagnifolium*).

Graham, E.H. 3021, 3619 (*elaeagnifolium*); 3822 (*hindsianum*).

Grant, G.B. 5550 (*elaeagnifolium*).

Griffiths, G. 216, 267 (*elaeagnifolium*).

Gross, C.A. 82 (*elaeagnifolium*).

Guaglianone, E.R. 1555, 1571 (*elaeagnifolium*).

Guerra, O. 601 (*elaeagnifolium*).

Guízar N., E. 4031 (*elaeagnifolium*).

Gullion, G.W. 383 (*elaeagnifolium*).

Gust, G. 2206 (*elaeagnifolium*).

Gutiérrez B., C. 436, 723, 977 (*houstonii*).

Guzmán C., U. 2121 (*houstonii*); 2947, 3013 (*elaeagnifolium*); 3279 (*houstonii*).

Guzmán, C.A. 11, 44 (*elaeagnifolium*).

Guzmán, R. 999 (*elaeagnifolium*).

Haene, E. 61 (*elaeagnifolium*).

Hahn, W. 1420 (*elaeagnifolium*).

Hall, E. 498 (*elaeagnifolium*).

Halse, R.R. 568 (*elaeagnifolium*).

Hammerly, B.J. 75, 216 (*hindsianum*).

Hanekom, W.J. 554 (*elaeagnifolium*).

Hannington, C. 23 (*elaeagnifolium*).

Hansen, B. 1392 (*elaeagnifolium*); 1402 (*houstonii*); 1791 (*elaeagnifolium*).

Hansen, C.J. 4421 (*elaeagnifolium*).

Hansen, G. 1174 (*elaeagnifolium*).

Hardham, C.B. 2628, 2804 (*elaeagnifolium*).

Harrington, H. 7966 (*elaeagnifolium*).

Hartman, C.V. 243 (*houstonii*).

Hartman, R.L. 65234 (*elaeagnifolium*).

Hartweg, K.T. 203 (*elaeagnifolium*).

Harvey, D.R. 585 (*elaeagnifolium*).

Hastings, J.R. 64-51 (*hindsianum*); 64-110 (*houstonii*); 63-123, 65-165, 64-169, 63-182, 66-187 (*hindsianum*); 65-197 (*houstonii*); 63-213, 64-300 (*hindsianum*).

Hawkes, J.G. 1166, 1463 (*elaeagnifolium*).

Helberg, J.J. 2528 (*elaeagnifolium*).

Heller, A.A. 1511, 3733, 4185 (*elaeagnifolium*).

Henderson, D.M. 6848 (*elaeagnifolium*).

Henrickson, J. 5708, 6673a, 7444, 7917 (*elaeagnifolium*).

Hernández A., C. 66 (*houstonii*).

Hernández A., F. 567 (*houstonii*).

Hernández Magaña, R. 207 (*houstonii*); 4642, 7714, 8185, 8352, 8415 (*elaeagnifolium*); 9027 (*houstonii*); 9752 (*elaeagnifolium*); 12115 (*houstonii*).

Hernández X., E. 677 (*houstonii*); X-2362, X-2493 (*elaeagnifolium*).

Hernández, A.C. 66 (*houstonii*).

Hernández, L. 1168, 1174 (*houstonii*).

Herter, W.G. 7290 (*elaeagnifolium*).

Hespenheide, H.A. 8 (*elaeagnifolium*).

Hick, P.M.L. 22, 30, 33 (*elaeagnifolium*).

Hieronymus, G. 288 (*elaeagnifolium*).

Higgins, L.C. 14434, 20971, 26756 (*elaeagnifolium*).

Hill, M. 225 (*elaeagnifolium*).

Hmama, H. 890 (*elaeagnifolium*).

Hopkins, M. 897 (*elaeagnifolium*).

Horr, W.H. 3521 (*elaeagnifolium*).

Hosseus, C.C. 65, 210, 233, 500, 1259, 2007, 2050, 2097, 2638 (*elaeagnifolium*).

Howell, J.T. 10610, 10611, 10643 (*hindsianum*); 12238, 26563, 39457, 47231 (*elaeagnifolium*).

Huckins, C.A. 6912-3002 (*hindsianum*).

Huidobro, A.M. 3514 (*elaeagnifolium*).

Hunziker, A.T. 20, 1030, 3252, 15924, 16035, 17347 (*elaeagnifolium*); 18322, 18333, 21889, 21895, 22764 (*mortonii*); 23318, 24860, 24868, 25089, 25228, 25356, 25385, 25395, 25396, 25403, 25554, 25936 (*elaeagnifolium*).

Hunziker, J.H. 66, 280, 347, 4097 (*elaeagnifolium*).

Hutchison, P. 701 (*hindsianum*).

Ibarrola, T.S. 151, 2955 (*elaeagnifolium*).

Igic, B. 07s20 (*houstonii*).

Illanes, A. 104 (*elaeagnifolium*).

Ingram, J. 650 (*hindsianum*).

Instituto de Investigaciónes Ecológicas Chiloé 502 (*elaeagnifolium*).

Irvine, F.R. 276 (*elaeagnifolium*).

Isern, J. 8096 (*elaeagnifolium*).

Iwen, F.A. 355 (*elaeagnifolium*).

Jafri, S.M.H. 3737 (*elaeagnifolium*).

Jiménez, J. 426 (*elaeagnifolium*).

Johansen, D.A. 551, 615 (*hindsianum*).

Johnson, D. 76, 130 (*hindsianum*).

Johnson, R.L. 12-029 (*elaeagnifolium*).

Johnston, I.M. 2158 (*elaeagnifolium*); 3063, 3178, 3302, 3421, 4201 (*hindsianum*).

Johnston, M.C. 256, 10443 (*elaeagnifolium*).

Jones, M.E. 4119 (*elaeagnifolium*); 22514, 23103, 24004 (*hindsianum*).

Jörgensen, P. 1070 (*elaeagnifolium*).

Joyal, E. 1339, 1423, 1520 (*houstonii*).

Juliani, H.R. 34, 35, 36, 37, 39 (*elaeagnifolium*).

Jury, S.L. 8776, 20869 (*elaeagnifolium*).

Jussel, M.S. 69 (*elaeagnifolium*).

Kadhum, H. 37772 (*elaeagnifolium*).

Karwinski, W.H. 583bis (*elaeagnifolium*); 583 [coll. 1841] (*hindsianum*); 583 [coll. 1842] (*elaeagnifolium*); 1399 (*houstonii*).

Keck, D.D. 2507 (*elaeagnifolium*); 4142 (*hindsianum*).

Kelley, H. 21 (*elaeagnifolium*).

Kiesling, R. 1049, 3006, 4241, 4652, 4827 (*elaeagnifolium*).

King, D.O. 148 (*elaeagnifolium*).

King, R.M. 2669, 2701 (*houstonii*).

Kipping, J. 308 (*hindsianum*).

Kishler, J. 145, 238, 579 (*elaeagnifolium*).

Knapp, S. IM-10078, 10470 (*elaeagnifolium*).

Knobloch, I.W. 70 (*elaeagnifolium*); 419 (*houstonii*).

Koch, S.D. 7493 (*houstonii*).

Kolberg, H. 925 (*elaeagnifolium*).

Krapovickas, A. 5950 (*elaeagnifolium*).

Kriebel, R. 8208 (*elaeagnifolium*).

Krishnappa, D.G. 162 (*elaeagnifolium*).

Kugel, A.R. 2080 (*elaeagnifolium*).

Kuhn, B. 2605 (*elaeagnifolium*).

Kurtz, F. 209, 911, 2604, 4222, 4224, 4744, 5117, 5209, 7901, 7993, 8034, 8086, 8945, 9503, 13117, 14206, 14406, 15732, 16036, 16090, 16090a (*elaeagnifolium*).

La Barrera, J.M. de 18, 20 (*elaeagnifolium*).

La Seta, A.V. de 255 (*elaeagnifolium*).

La Sota, A. de 132 (*elaeagnifolium*).

La Torre, C. de 47 (*elaeagnifolium*).

Lajtha, K. 123 (*elaeagnifolium*).

Lamond, J.M. 1399 (*elaeagnifolium*).

Landrum, L.R. 5409 (*houstonii*); 9537 (*elaeagnifolium*).

Lanfranchi, A.E. 35, 1315 (*elaeagnifolium*).

Langman, I.K. 2915 (*elaeagnifolium*); 3190 (*houstonii*).

Laukkonen, P. 333 (*elaeagnifolium*).

Leal, J. 29, 210 (*houstonii*).

Legler, B. 10779 (*elaeagnifolium*).

Legname, V. 221 (*elaeagnifolium*).

León de la Luz, J.L. 10161, 10591 (*hindsianum*).

Leonard, S.W. 3394 (*elaeagnifolium*).

Leuenberger, B. 2889 (*hindsianum*).

Leuenberger, B.E. 4459 (*elaeagnifolium*).

Levin, G.A. 2159 (*hindsianum*).

Linden, J.J. 243 (*houstonii*).

Lindheimer, F.J. fasc. I-135, III-135, 1041 (*elaeagnifolium*).

Liogier, A.H. 9732 (*elaeagnifolium*).

Lira Charco, E.M. 1379 (*houstonii*).

Lizama M., J. 1479 (*houstonii*).

Long, S. 1100 (*elaeagnifolium*).

López, ? 713 (*elaeagnifolium*).

López, L.E. 85 (*hindsianum*).

López Felix, ? 16 (*houstonii*).

López-Forment, W. 292, 422 (*hindsianum*).

López Franco, R.M. 974, 1100 (*houstonii*); 6335 (*elaeagnifolium*).

Lorentz, P.G. 9, 98, 99, 106, 320, 533, 905, 908, 1243 (*elaeagnifolium*).

Lossen, W. 12 (*elaeagnifolium*).

Lot, A. 454, 483, 604, 633, 646, 1877, 1922 (*houstonii*).

Lott, E.J. 73 (*elaeagnifolium*); 2016, 2114 (*houstonii*); 2469, 2501 (*hindsianum*); 5121, 5573 (*elaeagnifolium*).

Lourteig, A. 1019 (*elaeagnifolium*).

Luchetti, A.M. 4, 5, 6 (*elaeagnifolium*).

Luckow, M. 2837 (*hindsianum*).

Luna M., V.E. 36, 149, 262 (*houstonii*).

Luna, F.E. 1021 (*elaeagnifolium*).

Lundell, C.L. 5145 (*elaeagnifolium*); 7995 (*houstonii*); 8784 (*elaeagnifolium*).

Luz, J.L. de la 6121 (*hindsianum*).

MacDaniels, L.H. 443 (*houstonii*); 703 (*elaeagnifolium*).

MacDonald, J.R. 7551 (*elaeagnifolium*).

MacKee, H.S. 10974 (*houstonii*).

Maillet, J. 14 (*houstonii*).

Makings, E. 1102 (*elaeagnifolium*).

Mandon, E. 1010 (*elaeagnifolium*).

Manning, W.E. 531244 (*elaeagnifolium*).

Manríque, ? 1255 (*elaeagnifolium*).

Marais, W. 28703 (*elaeagnifolium*).

Marcus, E.J. 26245 (*elaeagnifolium*).

Marín, R. 150 (*elaeagnifolium*).

Marino, G. 201, 1914, 1942, 2047 (*elaeagnifolium*).

Marquez, M. 1095 (*houstonii*).

Marsh, E.G. 895 (*elaeagnifolium*).

Martin, H.C. 272 (*elaeagnifolium*).

Martínez Marín, J.L. 416, 464 (*elaeagnifolium*).

Martínez O., E. 413 (*elaeagnifolium*).

Martínez R., C. 104 (*elaeagnifolium*).

Martínez S., E.M. 4083, 39209 (*houstonii*); 39643, 39673, 39711, 40416, 40719 (*elaeagnifolium*).

Martínez, C. 1796 (*elaeagnifolium*).

Martínez, G.J. 314, 486, 524, 1321 (*elaeagnifolium*).

Martínez, J.L. 683 (*elaeagnifolium*).

Martínez, M. 861 (*houstonii*); 6618 (*hindsianum*); 6827 (*elaeagnifolium*).

Mason, H.L. 1903 (*hindsianum*); 8256, 13097 (*elaeagnifolium*).

Matesevach, M. 3 (*elaeagnifolium*).

Mathews, A. 238 (*elaeagnifolium*).

Matuda, E. 18953, 19545, 28754 (*elaeagnifolium*).

Mayfield, M.H. 301 (*houstonii*).

McClatchie, A.J. 1282 (*elaeagnifolium*).

McDaniel, T.F. 1856 (*hindsianum*).

McGregor, E.A. 857 (*elaeagnifolium*).

McKee, ? 10974 (*houstonii*).

Mearns, E.A. 122, 135, 619, 1698 (*elaeagnifolium*).

Medina A, M.E. 24 (*houstonii*).

Medina C., M. 2036 (*elaeagnifolium*).

Medina, V. 241 (*elaeagnifolium*).

Medina, W.A. 57 (*elaeagnifolium*).

Meglioli, S. 33 (*elaeagnifolium*).

Méndez, M. 261, 812 (*houstonii*).

Mereles, F. 4010, 5109, 9095 (*elaeagnifolium*).

Merello, M. 250 (*hindsianum*).

Mericle, L.W. 500 (*elaeagnifolium*).

Mertz, S.L. 148, 205 (*houstonii*).

Mertz, S.M. 15 (*elaeagnifolium*).

Metz, M.C. 3164 (*elaeagnifolium*).

Meyer, T. 565, 3506, 4046, 4067, 9863 (*elaeagnifolium*).

MFB 53 (*houstonii*).

Michener, D.C. 4253 (*hindsianum*).

Miehe, G. 277 (*elaeagnifolium*).

Miers, J. 258 (*elaeagnifolium*).

Miller, J.S. 7291 (*hindsianum*).

Miranda, F. 1418 (*elaeagnifolium*); 2119 (*houstonii*).

Miranda, R. FESC-RE-38 (*houstonii*).

Mooers, B.H.M. 854 (*elaeagnifolium*).

Molinar, R. 47 (*elaeagnifolium*).

Moncada, F. 4741 (*elaeagnifolium*).

Monroy M., R. 4 (*hindsianum*).

Montenegro, M.A. 380 (*elaeagnifolium*).

Montero Castro, J.C. 136 (*elaeagnifolium*).

Moore, H.E. 1640 (*elaeagnifolium*).

Mora-López, J.L. 189 (*houstonii*).

Moran, R. 3518, 3603, 3740, 3779, 7218, 8030, 9266, 11411, 22371 (*hindsianum*); 27703 (*elaeagnifolium*).

Moreno, P. 150, 494, 794 (*houstonii*).

Morong, T. 1190 (*elaeagnifolium*).

Moscone, E.A. 34, 70, 71, 109, 111, 161, 180, 183, 191, 245 (*elaeagnifolium*).

MRVG 503 (*hindsianum*).

Mulford, A.I. 684 (*elaeagnifolium*).

Muller, F. 182 (*houstonii*).

Nagahama, N. 129, 130, 131, 132 (*elaeagnifolium*).

Naranjo, C. 937 (*elaeagnifolium*).

Narváez C., J. 2 (*elaeagnifolium*).

Narváez, M. 339, 590 (*houstonii*).

Navarrete de la Paz, M. 33 (*houstonii*).

Nee, M. 11787, 11815, 18972 (*elaeagnifolium*); 23069 (*houstonii*); 24064, 25319 (*elaeagnifolium*); 26632 (*houstonii*); 57070, 57083 (*elaeagnifolium*).

Nelson, A. 1651, 11558 (*elaeagnifolium*).

Nelson, E.W. 6429 (*elaeagnifolium*); 7034, 7210 (*hindsianum*).

Nevling, L. 544, 1043 (*houstonii*).

Newman, M. 240 (*houstonii*); 315 (*elaeagnifolium*); 435 (*houstonii*).

Nicora, E.G. 1646, 2101, 4162, 4189, 4282, 8114 (*elaeagnifolium*).

Nixon, K.C. 838, 893 (*hindsianum*).

Norris, D.H. 20104 (*houstonii*).

Novara, L.J. 8248, 8504 (*elaeagnifolium*).

Novoa L., C.P. 54A (*houstonii*); 919 (*elaeagnifolium*).

O’Donell, C.A. 224, 1712, 1815, 1910 (*elaeagnifolium*).

Ocampo, G. 874 (*houstonii*).

Ocampo, R. 39 (*elaeagnifolium*).

Ochoa Avalos, S. 188 (*houstonii*).

Orbigny, A. d’ 223 (*elaeagnifolium*).

Orcutt, C.R. 680, 1270 (*elaeagnifolium*); 1339, 2793 (*hindsianum*); 3158, 3422 (*elaeagnifolium*); 4611, 4613 (*houstonii*).

Orea L., L. 153, 206, 274, 407, 440 (*houstonii*).

Orellana, R. 119BB (*houstonii*).

Orozco H, J. 9957, 10757 (*houstonii*).

Ortega O., R.V. O-720 (*houstonii*).

Ortega, R. 427, 708 (*houstonii*).

Osterhout, G.E. 2064 (*elaeagnifolium*).

Oswald, P.H.O. 15 (*elaeagnifolium*).

Palacios, ? 1695 (*elaeagnifolium*).

Palmer, E. 25 (*hindsianum*); 95 (*houstonii*); 101[b] (*elaeagnifolium*); 109 (*hindsianum*); 178 (*houstonii*); 233, 235 (*elaeagnifolium*); 237, 257, 314 (*houstonii*); 388, 935, 936, 941, 942 (*elaeagnifolium*); 1035 (*houstonii*); 2101, 7678 (*elaeagnifolium*).

Panti, M.A. 841 (*elaeagnifolium*).

Parish, S.B. 8104 (*elaeagnifolium*).

Parish, W.F. 172 (*elaeagnifolium*).

Parker, H.M. 379 (*elaeagnifolium*).

Parry, C.C. 536, 636 (*elaeagnifolium*).

Paula-Souza, J. 7856, 8046 (*elaeagnifolium*).

Pedersen, T.M. 5789, 5790, 8235 (*elaeagnifolium*).

Peebles, R.H. 7410 (*elaeagnifolium*).

Peña-Chocarro, M.C. 407, 1076 (*houstonii*); 1483 (*elaeagnifolium*).

Pensiero, J.F. 2424, 6063, 7209, 7374 (*elaeagnifolium*).

Pérez C., E. 3359 (*elaeagnifolium*).

Pérez H., S. 86 (*elaeagnifolium*).

Pérez Lara, M.A. 68, 94, 186, 612, 761, 860, 892 (*houstonii*).

Pérez S., G. 318 (*houstonii*).

Perkins, A.E. 3377 (*elaeagnifolium*).

Perrill, B. 5513 (*hindsianum*).

Peterson, J. 075 (*elaeagnifolium*).

Piccinini, ? 2046 (*elaeagnifolium*).

Pineda C., J.L. 477 (*houstonii*).

Plowman, T. 2711 (*elaeagnifolium*).

Poeppig, E.F. 26, 140, Diar540 (*elaeagnifolium*).

Popov, G.B. GP69266 (*elaeagnifolium*).

Price, T. 338 (*elaeagnifolium*).

Pringle, C.G. 1566 (*elaeagnifolium*); 5850 (*houstonii*); 9370 (*elaeagnifolium*).

Proctor, G.R. 30960, 49664 (*elaeagnifolium*).

Puch, A. 447, 825, 849, 1319 (*houstonii*).

Pujalte, J.C. 210, 259 (*elaeagnifolium*).

Purpus, C.A. 4548 (*elaeagnifolium*); 11048 (*houstonii*).

Quick, C.R. 69-15 (*elaeagnifolium*).

Ragonese, A.M. 6390 (*elaeagnifolium*).

Ramírez, O.P. 33 (*hindsianum*).

Ramírez, R. 1050 (*houstonii*).

Randolph, D. 173, 394 (*elaeagnifolium*).

Rangel Ramírez, R. 130, 330 (*elaeagnifolium*).

Rashid, A. 3692 (*elaeagnifolium*).

Rasp, A.E. 68 (*elaeagnifolium*).

Raven, P.H. 12484 (*hindsianum*); 16690 (*elaeagnifolium*).

Reales, A. 952, 968, 1615, 1678, 2015 (*elaeagnifolium*).

Rechinger, K.H. 29735, 29752, 63645 (*elaeagnifolium*).

Reddick, D. 157 (*houstonii*); 434 (*elaeagnifolium*).

Reed, F.M. 6190 (*hindsianum*).

Reeder, J.R. 6772 (*hindsianum*).

Reina G., A.L. 97-297, 2001-598, 98-2082, 98-2123 (*houstonii*).

Reko, P.B. 4462 (*houstonii*).

Reyes de los Santos, E. 147, 272, 367, 367, 477, 545, 662, 788, 871 (*houstonii*).

Reynoso Dueñas, J.J. 634 (*houstonii*).

Rico Arce, L. 378 (*houstonii*).

Rico-Gray, V. 421 (*houstonii*).

Riedel, J. 54 (*elaeagnifolium*).

Ringuelet, E.J. 255 (*elaeagnifolium*).

Rivera, R. 90 (*houstonii*).

Robles H., L. 397, 398 (*houstonii*).

Roderick, W. 3136 (*houstonii*).

Rodríguez C., A. 2107, 5076 (*houstonii*); 6100 (*elaeagnifolium*).

Rodríguez González, H. 143 (*houstonii*).

Rodríguez, ? (V)869 (*elaeagnifolium*).

Rodríguez, E. 42 (*elaeagnifolium*).

Rodríguez, J.P. 43 (*elaeagnifolium*).

Rojas, T. 1992, 13678 (*elaeagnifolium*).

Rollins, R.C. 2749 (*elaeagnifolium*).

Román Miranda, M.L. C-742, 1337 (*elaeagnifolium*); 1371, 1795 (*houstonii*).

Romero, S. 1405, 1406 (*elaeagnifolium*).

Rosas J., F. 1 (*houstonii*).

Rosas R., M. 876 (*houstonii*).

Rose, J.N. 2308, 10073 (*houstonii*).

Rose, L.S. 65121 (*elaeagnifolium*).

Ross, T.S. 6995, 7032 (*hindsianum*).

Roybal, J.J. 904 (*elaeagnifolium*).

Rubtzoff, P. 1321 (*elaeagnifolium*).

Ruiz Huidobro, A.M. 324 (*elaeagnifolium*).

Ruiz, L. 53398 (*elaeagnifolium*).

Runyon, R. 4194 (*elaeagnifolium*).

Ryder, V.P. 248 (*elaeagnifolium*).

Rzedowski, J. 16116 (*elaeagnifolium*); 26114 (*houstonii*); 29179 (*elaeagnifolium*); 33231, 46431 (*houstonii*).

Sagalyn, J.L. 69 (*elaeagnifolium*).

Salinas T., A. 7104 (*elaeagnifolium*).

Sallee, E.H. ES-54 (*elaeagnifolium*).

Sánchez Mejorada R, H. 80 (*elaeagnifolium*).

Sánchez, E.V. 46 (*houstonii*).

Sanders, A.C. 19612, 25026 (*elaeagnifolium*).

Santana Michel, F.J. 546, 2955 (*houstonii*).

Saravia Toledo, C. 2336a (*elaeagnifolium*).

Sargent, F.H. 513 (*elaeagnifolium*).

Saucedo, E. 338 (*hindsianum*).

Sauleda, R. 2649 (*elaeagnifolium*).

Sayago, M. 570 (*elaeagnifolium*).

Scaldaferro, M. 21, 25 (*elaeagnifolium*).

Schaffner, J.G. 695 (*elaeagnifolium*).

Schallert, P.O. 540 (*elaeagnifolium*).

Schickendantz, F. 80, 82 (*elaeagnifolium*).

Schinini, A. 37059 (*elaeagnifolium*).

Schlottmann, A. 269 (*elaeagnifolium*).

Schmitz, A. 166[a], 166[b] (*elaeagnifolium*).

Scholl, M.R. 26 (*elaeagnifolium*).

Schott, A.C.V. 310 (*houstonii*).

Schreiter, R. 12408 (*elaeagnifolium*).

Schulz, C.L. 1363, 5590 (*elaeagnifolium*).

Schulz, E.D. 97 (*elaeagnifolium*).

Schumann, W. 970 (*elaeagnifolium*).

Schwarz, G.J. 9758 (*elaeagnifolium*).

Scora, R.W. 2285 (*elaeagnifolium*).

Scott-Elliot, G.F. 442 (*elaeagnifolium*).

Selander, R.B. 21, 4-53, 6-53 (*elaeagnifolium*).

Serrano, A. 128 (*elaeagnifolium*).

Sessé, M. 1532 (*elaeagnifolium*); 1538, 1538bis, 5367 (*houstonii*); 5382 (*elaeagnifolium*).

Sherwin, P. 226 (*hindsianum*).

Shevock, J. 502, 12209 (*elaeagnifolium*).

Shreve, F. 6446 (*hindsianum*).

Silva R., L. 184, 187 (*elaeagnifolium*).

Simá, P. 38 (*houstonii*).

Singh, V. 80328 (*elaeagnifolium*).

Siqueiros, M. 515 (*elaeagnifolium*).

Six, I.L. 3409-84 (*elaeagnifolium*).

Skehan, J. 16 (*elaeagnifolium*).

Small, J.K. 4841 (*elaeagnifolium*).

Smith, G.L. 1030 (*elaeagnifolium*).

Sohmer, S.H. 9372 (*houstonii*).

Solbrig, O.T. 140 (*elaeagnifolium*).

Soriano M., A.M. 144 (*elaeagnifolium*).

Soriano, A. 548, 1143 (*elaeagnifolium*).

Soto N. [Soto Nuñez], J.C. 9508 (*elaeagnifolium*).

Sousa Peña, M. 57, 79, 102, 133,146, 175 (*hindsianum*).

Sousa S., M. 4836 (*houstonii*).

Souviron, M.J. 27 (*houstonii*).

Sparrow, C.E.H. 46 (*elaeagnifolium*).

Spegazzini, P.L. 28174, 33629 (*elaeagnifolium*).

Spencer, M.F. 1417 (*elaeagnifolium*).

Spetzman, L. 1366 (*elaeagnifolium*).

Sprankle, J.A. 96 (*elaeagnifolium*).

St Pierre, M. 2601 (*elaeagnifolium*).

Stamatiadou, E. 13340, 15571 (*elaeagnifolium*).

Stanford, L.R. 273, 586, 802 (*elaeagnifolium*).

Starlinger, F. 22-93 (*elaeagnifolium*).

Steere, W.C. 1065 (*houstonii*).

Steibel, P. 4092 (*elaeagnifolium*).

Stephens, S. 10953 (*elaeagnifolium*).

Stevens, G.W. 802, 889 (*elaeagnifolium*).

Steyermark, J.A. 83071 (*elaeagnifolium*).

Stiefkens, L.B. 4 (*elaeagnifolium*).

Stimson, W.R. 1403 (*elaeagnifolium*).

Stofella, ? 328 (*elaeagnifolium*).

Stroh, A.L. 69.63.02 (*elaeagnifolium*).

Strother, J.L. 258 (*elaeagnifolium*).

Stuckert, T.J.V. 1239, 1720, 2153, 2409, 2981, 3347, 3510, 4027, 4157, 4300, 4696, 6152, 6517, 7049, 8103, 8335, 8591, 8592, 8689, 9286, 9337, 10520, 11278, 11590, 12070, 15263, 15264, 15745, 16858, 16968, 19448, 19468, 19933, 23189, 23783 (*elaeagnifolium*).

Suárez Gracida, C.G. 2006-25 (*hindsianum*).

Subils, R. 2909, 3589b, 3699, 3881, 4414, 4463 (*elaeagnifolium*).

Sueltenfuse, C. 193 (*elaeagnifolium*).

Symon, D.E. 14318, 15128 (*elaeagnifolium*).

Takaki, F. 326 (*elaeagnifolium*).

Tapia, J.L. 1533 (*houstonii*).

Taylor, C.M. 1898 (*elaeagnifolium*).

Tejero Diez, D. 6256, 6517 (*elaeagnifolium*).

Téllez V., O. 1576, 3766, 11198 (*houstonii*).

Temple, L.C. 5513, 12151 (*elaeagnifolium*).

Templeton, B. 8644 (*elaeagnifolium*).

Tenorio L., P. 1122 (*elaeagnifolium*); 1306 (*hindsianum*); 4644 (*houstonii*); 9481, 10526, 10643, 10955, 10991, 12824 (*hindsianum*); 13676 (*elaeagnifolium*).

Terribile, M. 808 (*elaeagnifolium*).

Thackery, F.A. 226 (*elaeagnifolium*).

Tharp, B.C. 52-78, 51-261 (*elaeagnifolium*).

Thomas L., E. 13 (*houstonii*).

Thomas, J.H. 111 (*hindsianum*); 7683 (*houstonii*); 8285, 8428 (*hindsianum*); 9978a (*elaeagnifolium*).

Thomas, R.D. 96112 (*elaeagnifolium*).

Thornber, J.J. 482 (*elaeagnifolium*).

Thorne, R.F. 58047 (*hindsianum*).

Tilton, D. 218, 229, 236, 237, 241, 266, 267, 321, 330, 337, 353, 369, 374, 399 (*elaeagnifolium*).

Tirado, N. 2, 51 (*elaeagnifolium*).

Tolstead, W.L. 5859 (*elaeagnifolium*).

Torke, K.J. 381 (*houstonii*).

Torrecillas, E. 208 (*elaeagnifolium*).

Torres C., R. 3141, 3247, 3561, 3939 (*houstonii*); 15684, 17481 (*elaeagnifolium*).

Torres, P. 21 (*houstonii*).

Torriglia, M. 327 (*elaeagnifolium*).

Toumey, J.W. 398 (*elaeagnifolium*).

Tracy, S.M. 7576 (*elaeagnifolium*).

Traverse, A. 802 (*elaeagnifolium*).

Trejo, L. 13, 53 (*houstonii*).

Troncoso, N.S. 6030 (*elaeagnifolium*).

Tun, F. 418, 443 (*houstonii*).

Turland, N.J. 671 (*elaeagnifolium*).

Tweedie, J. 29, 70 (*elaeagnifolium*).

Twisselmann, E.C. 1266, 2248, 4791, 4918, 9088, 10232, 14887, 15599 (*elaeagnifolium*).

Ucán Ek, E. 277, 394, 1249, 1826, 2332, 3021, 3451, 4629, 4635 (*houstonii*).

Uh Cauich, R. 4 (*houstonii*).

Unsicker, J. 134 (*elaeagnifolium*).

Urdampilleta, J.D. 583 (*elaeagnifolium*).

Valdés, J. 55-2, 15-1978 (*elaeagnifolium*).

Valiente Banuet, A. 666 (*hindsianum*).

Valov, D. 2004-218 (*hindsianum*).

Van Devender, T.R. 2005-10 (*houstonii*); 98-29 (*hindsianum*); 99-379 (*elaeagnifolium*).

Vanni, R.O. 2620 (*elaeagnifolium*).

Vara, A. 157 (*houstonii*).

Varela E., L. 96-184, 96-348, 97-01 (*houstonii*).

Varela, F. 713 (*elaeagnifolium*).

Varela, F.J. de 585 (*elaeagnifolium*).

Vargas, C. 68 (*houstonii*).

Vasey, G.R. 351 (*elaeagnifolium*).

Vázquez Yanes, C. 653 (*houstonii*).

Vázquez, J. 2164 (*elaeagnifolium*).

Vega Aviña, R. 267, 1467, 1595, 9884 (*houstonii*).

Ventura A., A. 1361, 1509, 1595 (*elaeagnifolium*).

Ventura A., F. 3385, 10560, 11718 (*houstonii*).

Ventura, E. 6112, 6812, 8022, 8329 (*elaeagnifolium*).

Ventura, M.A. 127 (*houstonii*).

Venturi, S. 63, 1091, 7132, 7678, 9882 (*elaeagnifolium*).

Vilcapoma, G. 520, 540 (*elaeagnifolium*).

Villafañe, A. 937, 1028, 1265 (*elaeagnifolium*).

Villafañe, M. 36, 903, 993, 1234, 1271 (*elaeagnifolium*).

Villalobos-Mejía, ? 254 (*houstonii*).

Villaneuva G., R. 11 (*houstonii*).

Villarreal de Puga, L.M. 11507, 16135 (*houstonii*).

Villegas F., E. 219, 329 (*houstonii*).

Vine, R.S. 81 (*elaeagnifolium*).

Volponi, C.R. 240, 241 (*elaeagnifolium*).

Wagner, R.J. 1559, 1646, 1880 (*elaeagnifolium*).

Ward, D.B. 5742, 5771 (*elaeagnifolium*).

Wawra, H. 673 (*houstonii*).

Way, M.J. WSSB-3 (*elaeagnifolium*); WS-023, MJW-107 (*houstonii*).

Weaver Jr., R.E. 2044 (*elaeagnifolium*).

Webster, G.L. 15557, 17551 (*houstonii*); 19749 (*hindsianum*); 19916 (*houstonii*); 24269, 26022 (*hindsianum*).

Welsh, S.L. 25320 (*elaeagnifolium*).

Werdermann, E. 142 (*elaeagnifolium*).

Whalen, M.D. 13, 16, 182 (*houstonii*).

Wheeler, H.-.M. 5859 (*elaeagnifolium*).

White, D. 96 (*houstonii*).

White, L.D. 6 (*elaeagnifolium*).

White, S.D. 4526 (*elaeagnifolium*).

White, S.S. 183, 1180, 1712, 2052, 2526, 2574 (*elaeagnifolium*).

Whiting, A.F. 905 (*elaeagnifolium*).

Whitson, M. 803, 817 (*elaeagnifolium*).

Wickens, G.E. 828 (*elaeagnifolium*).

Wiggins, I.L. 78 (*hindsianum*); 326, 382 (*houstonii*); 444 (*elaeagnifolium*); 595, 596, 4300, 4366, 6065 (*hindsianum*); 6393, 7333, 7344 (*houstonii*); 7616, 8326, 11305 (*hindsianum*); 11750, 11778, 11911 (*elaeagnifolium*); 13150 (*houstonii*); 14619, 14809A, 15648, 16928, 16972, 17019, 17219, 17290, 17360, 20895 (*hindsianum*).

Wilbur, R.L. 2469 (*houstonii*); 35155, 35189 (*elaeagnifolium*); 35210 (*houstonii*); 36096 (*elaeagnifolium*).

Wilczek, C. 239 (*elaeagnifolium*).

Williams, M.J. 82-26-1, 79-176-1 (*elaeagnifolium*).

Wisura, W. 4843 (*hindsianum*).

Wittman, R.C. 18438 (*elaeagnifolium*).

Wolf, C.B. 2485, 2558, 3086 (*elaeagnifolium*).

Wooton, E.O. 59, 3127 (*elaeagnifolium*).

Worth, C.R. 8815 (*houstonii*); 16215 (*elaeagnifolium*).

Wright, C. 1590 (*elaeagnifolium*).

Wright, W.R. 1289 (*houstonii*).

Wynd, F.L. 76, 780 (*elaeagnifolium*).

Xantus de Vesey, J. 84 (*hindsianum*).

York, C.L. 52-300 (*elaeagnifolium*).

York, H.H. 49 (*elaeagnifolium*).

Zamora C., P. 143, 282, 541, 598, 634 (*houstonii*).

Zamudio, S. 2097, 2212 (*houstonii*); 2305 (*elaeagnifolium*); 2483, 2868, 10249 (*houstonii*).

Zanoni, T. 33168 (*elaeagnifolium*).

Zardini, E.M. 36391 (*elaeagnifolium*).

Zohner, O. 34 (*elaeagnifolium*).

Zola B., M.G. 578, 1584, 2135, 2179 (*houstonii*).

Zuloaga, F.O. 9373 (*elaeagnifolium*).
